# Assembly of Bioactive Superstructures via Metal–Phenolic Complexation for Blood Purification

**DOI:** 10.1002/anie.2081750

**Published:** 2026-08-05

**Authors:** Po Wang, Jingqu Chen, Siying Chen, Binsha Peng, Zhifeng Zhou, Ling Zhang, Rui Xie, Xiaojie Ju, Wei Wang, Dawei Pan, Liangyin Chu, Frank Caruso, Zhuang Liu

**Affiliations:** ^1^ School of Chemical Engineering Sichuan University Chengdu Sichuan P. R. China; ^2^ Department of Chemical Engineering The University of Melbourne Parkville Victoria Australia; ^3^ Department of Nephrology Institute of Kidney Diseases West China Hospital of Sichuan University Chengdu China

**Keywords:** anticoagulant, antimicrobial, bioactive materials, metal–organic frameworks, supramolecular chemistry

## Abstract

Hyperbilirubinemia, which stems from bilirubin accumulation caused by liver failure, can lead to jaundice, multi‐organ damage, and mortality. Hemoperfusion is the most effective detoxification approach, and its efficacy critically depends on the adsorbent system. However, most developed adsorbents exhibit poor hemocompatibility, posing risks of coagulation and bacterial infection. Herein, we report a facile and highly biocompatible strategy to fabricate bioactive superstructures for blood purification, enabling toxin removal. The formation of the bioactive superstructures is mediated by the assembly of polydopamine/Zn^II^ networks on natural polymer templates, followed by surface modification with human serum albumin. The obtained superstructures enable rapid bilirubin clearance owing to the synergistic effects of metal coordination and polyphenol‐mediated noncovalent interactions (hydrogen bonding and hydrophobic and electrostatic interactions). Furthermore, these bioactive superstructures exhibit excellent antibacterial efficacy, achieving > 99.9% killing efficiency of both *Escherichia coli* and *Staphylococcus aureus*, while enhancing anticoagulant performance by 2.6‐fold (compared to the control) through inhibition of platelet adhesion/activation and modulation of the intrinsic coagulation pathway. This work expands our understanding of metal–phenolic‐mediated superstructure assembly. Furthermore, the efficient toxin removal, antibacterial protection, and anticoagulant activity of the developed bioactive superstructures are expected to underpin the rational design of blood‐contacting materials based on metal–phenolic complexation.

## Graphical abstract

Bioactive superstructures are assembled via metal–phenolic‐mediated assembly on agarose templates, followed by modification with human serum albumin. These superstructures demonstrate high bilirubin adsorption efficiency, strong antibacterial activity, and anticoagulant activity, providing a strategy for the rational design of multifunctional blood‐contacting materials.

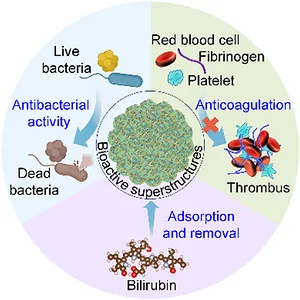

## 1 Introduction

Hyperbilirubinemia results from impaired bilirubin metabolism or excretion, leading to the accumulation of free bilirubin in the bloodstream and subsequent damage to the nervous system, liver, and other organs [1]. In severe cases, this condition can progress to brain injury, hepatic encephalopathy, or death [2]. Hemoperfusion is currently one of the most effective extracorporeal therapies for removing toxins, such as bilirubin [3], with its efficacy largely relying on the selectivity, loading capacity, and binding kinetics of adsorbents [4]. Widely used adsorbents, including activated carbon [5], ion‐exchange resins [6], and nano/porous materials (such as carbon nanotubes [7], mesoporous carbons [8], and metal–organic frameworks [9, 10], exhibit high adsorption capacity and selectivity. However, these adsorbents often trigger inflammatory responses and pose the risk of inducing coagulation and complement activation [3, 11]. Furthermore, bacterial contamination during hemoperfusion presents additional safety and efficacy concerns [12]. Therefore, there is a need to develop hemoperfusion adsorbents that integrate high bilirubin binding and clearance efficiency with antibacterial and anticoagulant functionalities.

Polyphenols, a class of naturally abundant compounds [13], exhibit high biocompatibility [14] and possess anti‐inflammatory properties that may facilitate the mitigation of inflammatory responses during blood contact [15]. In addition, polyphenols provide high‐affinity binding interfaces for hydrophobic toxins such as bilirubin [16, 17, 18]. In particular, metal–phenolic network (MPN)‐mediated assembly [19, 20] offers a high degree of modularity, enabling versatile integration of the antibacterial properties of metal ions (e.g., Zn^II^), anti‐inflammatory properties of phenolic ligands, and anticoagulant properties of specific biomolecules (e.g., human serum albumin (HSA)) within a single framework [21]. More importantly, this assembly approach allows nanoscale building blocks to be hierarchically organized at the microscale, forming hierarchical superstructures [22, 23, 24]. These superstructures increase the accessible surface area and adsorption interfaces [25], thereby increasing the likelihood of enhanced adsorption efficiency of bilirubin. To this end, we hypothesize that MPN‐mediated assembly would serve as a powerful and scalable strategy for designing blood perfusion adsorbents that combine high binding activity, antibacterial properties, and hemocompatibility.

In the present study, zinc (Zn^II^) ammonium and dopamine (DA) are selected as metal–phenolic building blocks to construct polydopamine (PDA)–Zn^II^ networks (denoted as PDA/Zn^II^). Immersion of agarose (Aga), a natural polysaccharide, in the PDA/Zn^II^ solution then leads to the assembly of superstructures (Aga‐PDA/Zn^II^) (Scheme 1a). The formation of the superstructures is predominantly driven by metal coordination and hydrophobic interactions, with additional contributions from hydrogen bonding and electrostatic interactions. The resulting superstructures exhibit a typical hierarchical composite architecture—on the macroscopic level, the Aga template is preserved, microspheres of PDA/Zn^II^ (approximately 1 µm in diameter) are distributed across the surface and the interior of the Aga template, and a nanoporous network structure is formed on the surface of the PDA/Zn^II^ microspheres. Furthermore, the abundant phenolic motifs on the Aga‐PDA/Zn^II^ surface afford secondary incorporation of HSA (a biomolecule with high hemocompatibility and antithrombogenic properties) [26], yielding bioactive superstructures (i.e., **H**SA‐**A**ga‐**P**DA/Zn^II^; simply denoted as HAP/Zn^II^; Scheme 1a). The modularity of the superstructures enables facile integration of antibacterial (conferred by Zn^II^), anti‐inflammatory (derived from phenolic ligands), and anticoagulant (conferred by HSA) properties, together with high bilirubin removal efficiency arising from the hierarchical structure of the adsorbent matrix (Scheme 1b). Notably, these bioactive superstructures provide abundant accessible binding sites and mass‐transfer pathways, thereby facilitating efficient bilirubin capture under complex biological conditions. This work presents a metal–phenolic‐mediated assembly approach for constructing hierarchical bioactive superstructures that combine high adsorption efficiency, strong antibacterial activity, and anticoagulant activity, providing a feasible and promising approach for the development of blood‐contacting materials.

**SCHEME 1:**
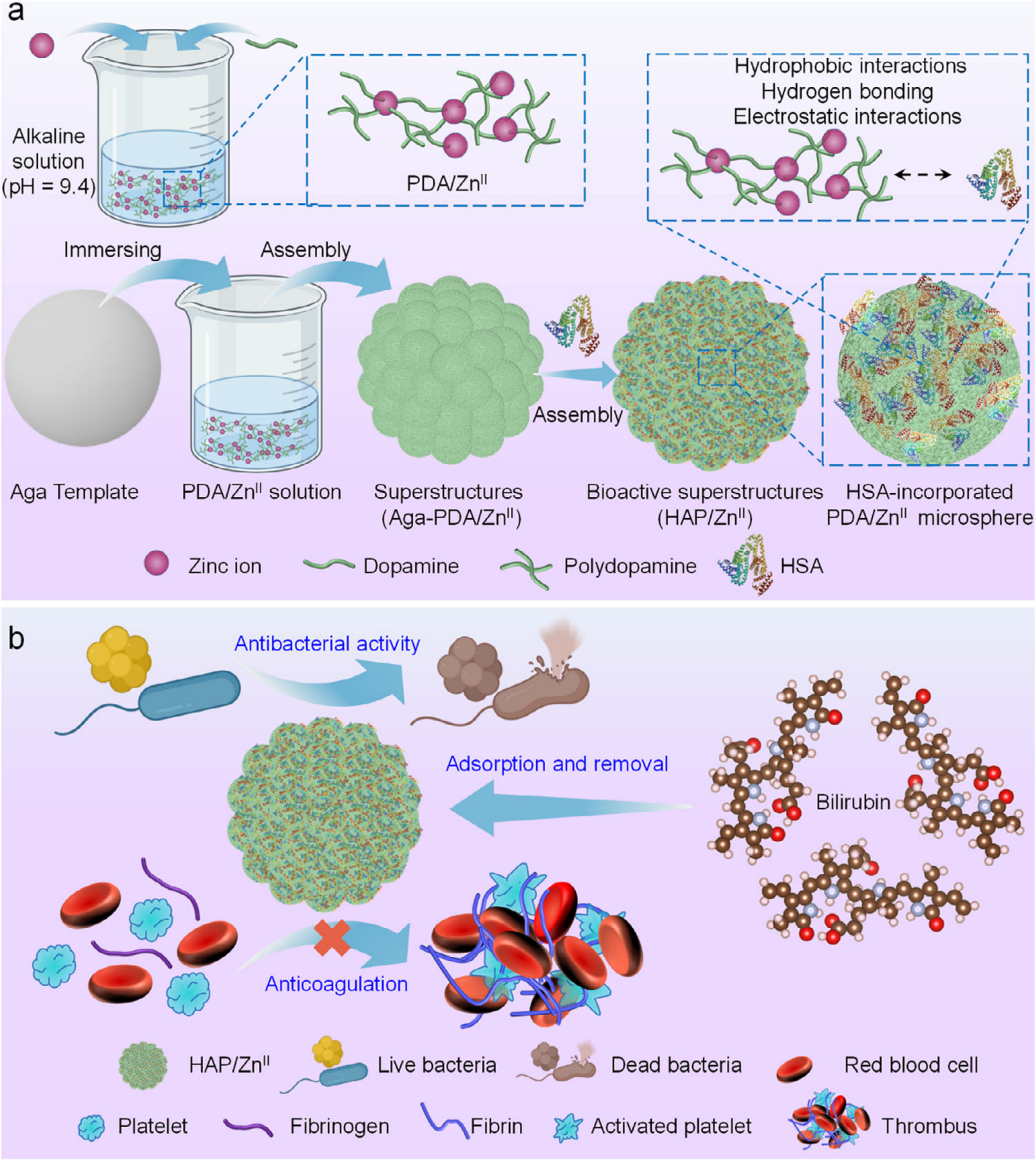
(a) Schematic of the formation of superstructures, Aga‐PDA/Zn^II^, via metal–phenolic‐mediated assembly on an Aga template, followed by modification with HSA to obtain bioactive superstructures, HAP/Zn^II^. (b) Schematic illustrating the antibacterial and anticoagulant properties, as well as the bilirubin removal capability, of the HAP/Zn^II^ bioactive superstructures.

## 2 Results and Discussion

### 2.1 Preparation of Superstructures

For the preparation of Aga‐PDA/Zn^II^ superstructures, DA and Zn^II^ were first mixed in an alkaline solution (pH = 9.4), enabling the oxidative polymerization of DA into PDA and the concurrent formation of PDA/Zn^II^ networks [27, 28]. Subsequent immersion of natural polymer template Aga microspheres (524 ± 36 µm in phosphate‐buffered saline (PBS)) in the PDA/Zn^II^ solution at room temperature resulted in the assembly of Aga‐PDA/Zn^II^ (Scheme 1a). Scanning electron microscopy (SEM) and transmission electron microscopy (TEM) images revealed that Aga‐PDA/Zn^II^ exhibits a hierarchical composite structure, with densely packed PDA/Zn^II^ microspheres (∼1 µm in diameter) uniformly distributed across both the surface and interior regions of the Aga template (Figures 1a–c and S1). Energy‐dispersive x‐ray spectroscopy confirmed the uniform distribution of C, N, and O elements from PDA and Zn from the zinc–ammonia solution throughout the microspheres (Figure 1d). Notably, each individual PDA/Zn^II^ microsphere displayed a nanoscale porous surface network (Figures 1a and S1e), whereas this structure was absent from both the pristine Aga templates and Aga modified solely with PDA (i.e., Aga‐PDA) (Figure S1a–d). The differential intrusion curve of Aga‐PDA/Zn^II^ displayed a pronounced peak in the range of 5–100 nm, indicating the presence of increased open pore volume at the mesoporous scale (Figure 1e). In addition, Aga‐PDA/Zn^II^ exhibited a specific surface area of 139.1 m^2^ g^−1^, which is significantly larger than that of Aga (i.e., 75.5 m^2^ g^−1^). The formation of Aga‐PDA/Zn^II^ superstructures also led to a substantial improvement in porosity and total pore volume compared to the pristine Aga template. Specifically, the porosity increased from 73.3% to 84.4%, while the total pore volume expanded from 2.1 to 4.1 mL g^−1^. Compared to Aga‐PDA/Zn^II^, modification with PDA alone (i.e., Aga‐PDA) showed a less pronounced increase in these parameters (Figure 1f). These results collectively suggest that the networks (formed by Zn^II^ and PDA) may interact with the interparticle pores of the Aga template, leading to the formation of a hierarchical nano‐micro‐macro composite structure [29, 30]. This hierarchical structure is envisioned to afford Aga‐PDA/Zn^II^ with high mass transport efficiency, stability in the bloodstream, and abundant active sites for bacterial and biomolecule binding [29], and the ability to generate localized mechanical stress that may induce bacterial membrane deformation [31]. Collectively, these attributes make Aga‐PDA/Zn^II^ a promising candidate for hemoperfusion applications. Furthermore, thermogravimetric analysis revealed a significantly lower mass loss for Aga‐PDA/Zn^II^ than for Aga or Aga‐PDA, indicating that Zn^II^ was present in these superstructures (Figure S2).

**FIGURE 1:**
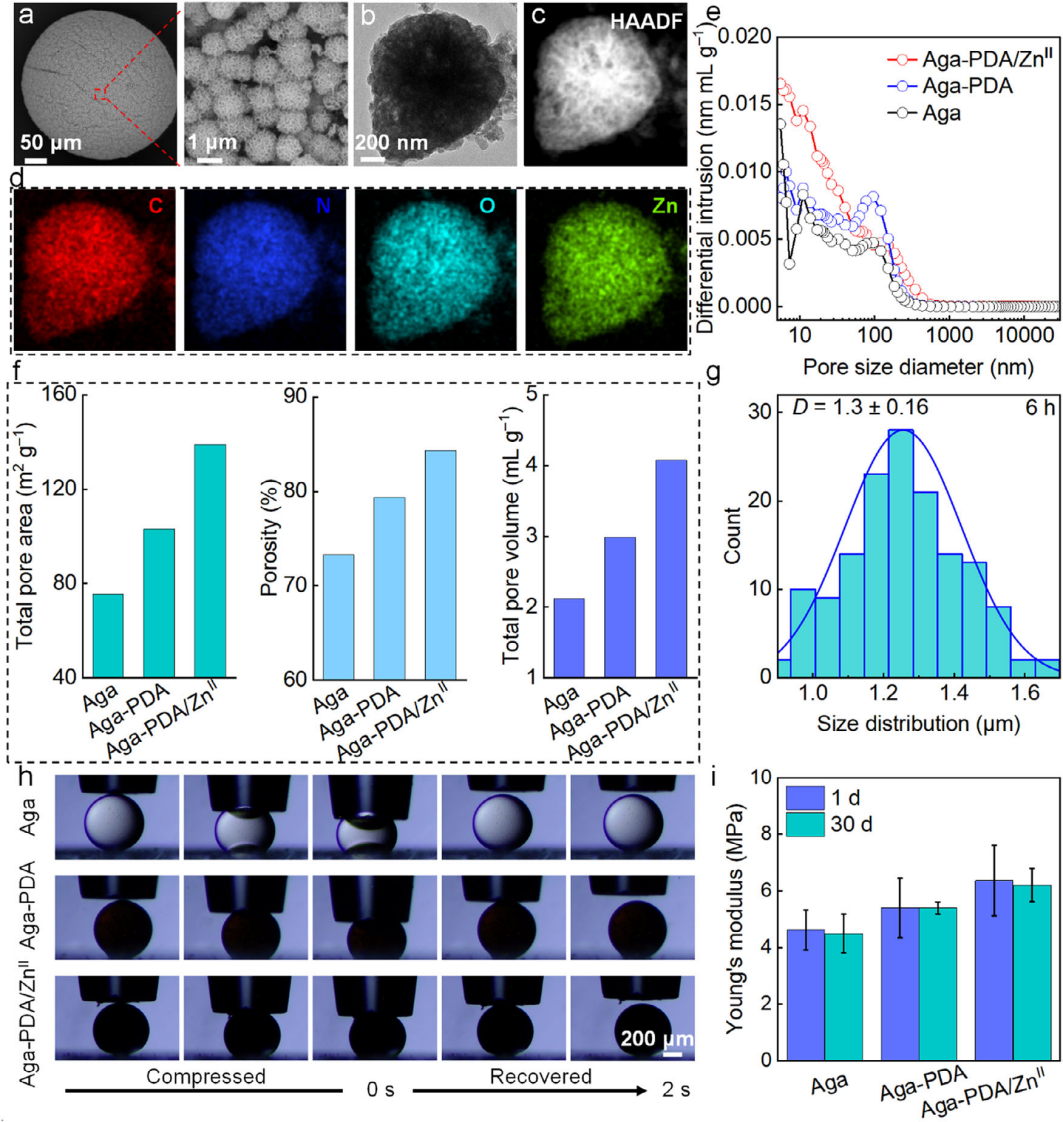
Characterization of Aga‐PDA/Zn^II^ superstructures. (a) SEM images of Aga‐PDA/Zn^II^ superstructures and individual PDA/Zn^II^ microspheres on an Aga template surface. (b–d) TEM image, high‐angle annular dark‐field (HAADF)‐STEM image, and energy‐dispersive x‐ray spectroscopy elemental mapping of a single PDA/Zn^II^ microsphere. (e) Pore size distributions of Aga, Aga‐PDA, and Aga‐PDA/Zn^II^. (f) Total pore area, porosity, and total pore volume of Aga, Aga‐PDA, and Aga‐PDA/Zn^II^. (The absolute error of the mercury intrusion porosimeter for measurement is ±1%.) (g) Size distribution of PDA/Zn^II^ microspheres on the surface of Aga‐PDA/Zn^II^ superstructures (assembly time: 6 h). (h) Compression–recovery behavior of Aga, Aga‐PDA, and Aga‐PDA/Zn^II^ measured on a microparticle strength tester. (i) Young's modulus of Aga, Aga‐PDA, and Aga‐PDA/Zn^II^ before and after immersion in buffer solution for 1 or 30 days.

The effect of assembly time on the size and zinc content of the PDA/Zn^II^ microspheres was subsequently investigated. After 1 h of assembly, the PDA/Zn^II^ microspheres exhibited a size of 0.9 µm and were sparsely distributed on the surface of the Aga template (Figure S3a,e). When the assembly time was extended to 2 h, the microspheres became more densely packed on the Aga surface, with their size slightly increasing to 1.0 µm (Figure S3b,f). At 6 h post‐assembly, the surface of the Aga template was nearly fully covered with PDA/Zn^II^ microspheres, with their size slightly increasing to 1.3 µm (Figures 1g and S3c). Further extension of the assembly time to 12 h exerted a negligible impact on the packing density of microspheres and their size (Figure S3d,g). Quantitative analysis of the zinc content via inductively coupled plasma mass spectrometry (ICP‐MS) after dissolution of the PDA/Zn^II^ microspheres on the Aga template using 10‐fold‐diluted hydrochloric acid revealed a progressive increase in Zn^II^ with assembly time. Specifically, Zn^II^ content was 50.0 mg g^−1^ at 1 h, which gradually increased to 114.8 mg g^−1^ after 6 h, and plateaued thereafter (Figure S4). Accordingly, Aga‐PDA/Zn^II^ superstructures obtained using an assembly time of 6 h were selected for subsequent studies.

The mechanical strength of adsorbents is a key parameter in hemoperfusion applications [3]. Strength tests of Aga, Aga‐PDA, and Aga‐PDA/Zn^II^ were conducted using a microparticle strength tester. All three tested materials recovered their original shape within 2 s after compression (Figure 1h). The Young's modulus was calculated from the force–displacement curves (Figure S5)—the Young's modulus of the Aga‐PDA/Zn^II^ superstructures was significantly higher (6.38 MPa) than that of the pristine Aga (4.63 MPa) and Aga‐PDA (5.40 MPa). This enhancement can be attributed to the strong coordination between Zn^II^ and PDA, which act as additional cross‐linking sites, thereby increasing the cross‐linking density and stiffness of the material. In addition, the compactly packed PDA/Zn^II^ microspheres reduced interfacial slippage, enhanced stress transfer efficiency, and suppressed plastic deformation, resulting in a higher Young's modulus for Aga‐PDA/Zn^II^. Immersion in PBS buffer (pH 7.4) for 30 days negligibly influenced the Young's modulus of Aga‐PDA/Zn^II^, which remained unchanged (Figure 1i). To evaluate the repeated compression resistance of the prepared materials (i.e., Aga, Aga‐PDA, and Aga‐PDA/Zn^II^), the latter were subjected to three consecutive compression–recovery cycles. The Young's modulus of Aga, Aga‐PDA, and Aga‐PDA/Zn^II^ showed negligible variations during the repeated tests, with changes of less than 5% (Figure S6). These results indicate that the hierarchical composite architecture of Aga‐PDA/Zn^II^ significantly enhances the stiffness and strength of the Aga template, while maintaining its intrinsic stability.

### 2.2 Mechanistic Insights Underlying the Formation of Aga‐PDA/Zn^II^ Superstructures

Next, we investigated the mechanism underlying the formation of Aga‐PDA/Zn^II^ superstructures via a range of techniques. Raman spectra of Aga‐PDA and Aga‐PDA/Zn^II^ demonstrated that both amino (─NH) and hydroxyl (─OH) groups from PDA coordinated with Zn^II^ through the formation of Zn─O and Zn─N bonds, as deduced by a reduction in the intensity of the ─NH bending vibration peak at 1519 cm^−1^ and a shift of the ─OH in‐plane bending/C─NH vibration peak from 1399 to 1386 cm^−1^ (Figure 2a). Furthermore, the peak assigned to C─O or C─N stretching vibration at 1347 cm^−1^ shifted to 1334 cm^−1^, also suggesting an increased degree of cross‐linking via Zn^II^‐mediated coordination [32].

**FIGURE 2:**
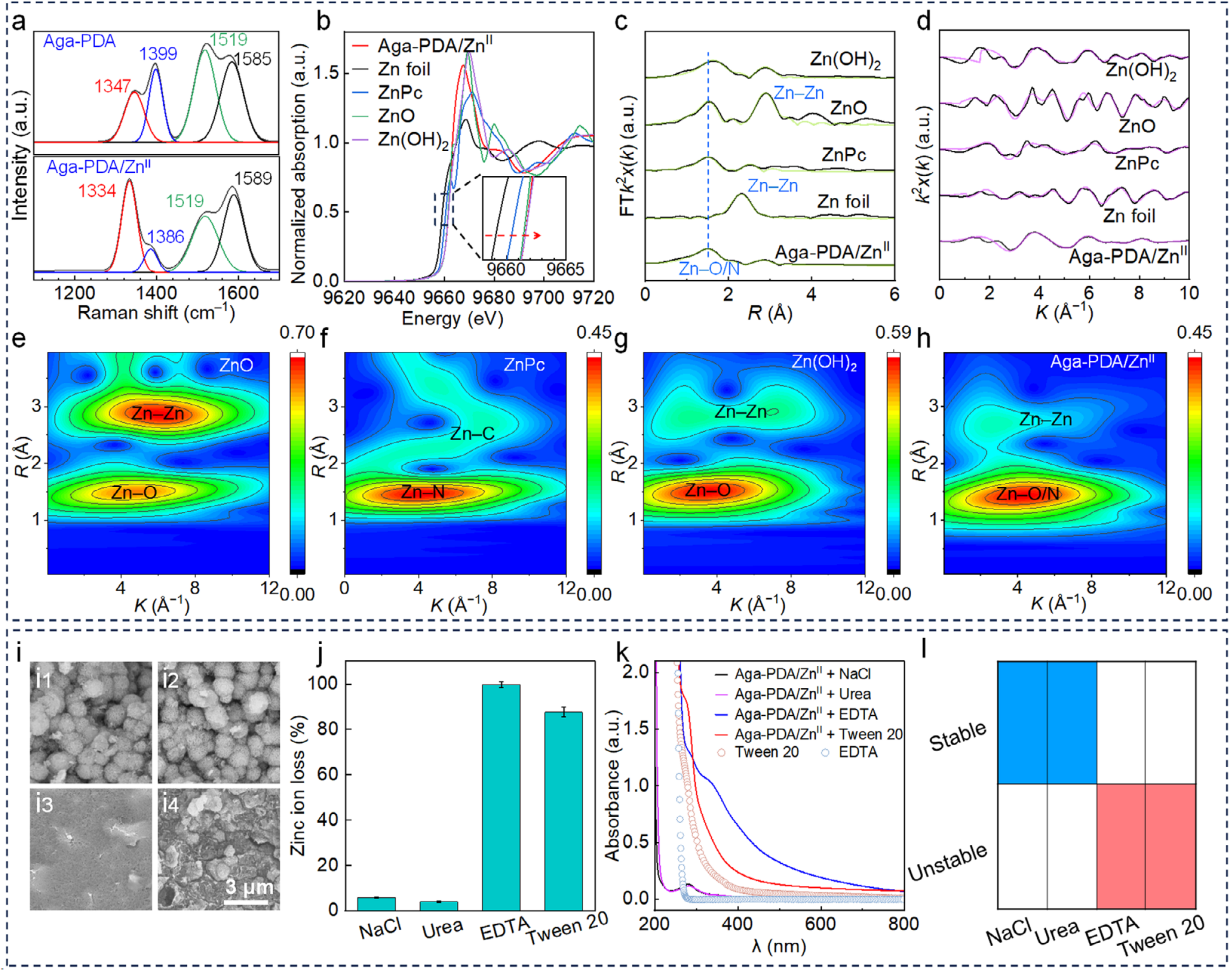
Mechanistic insights into the formation and physicochemical properties of Aga‐PDA/Zn^II^ superstructures. (a) Raman spectra of Aga‐PDA and Aga‐PDA/Zn^II^. (b) XANES spectra of Aga‐PDA/Zn^II^ and reference Zn foil, ZnPc, ZnO, and Zn(OH)_2_. (c,d) FT spectra of Aga‐PDA/Zn^II^ in *R*‐space (c) and *K*‐space (d). (e–h) Wavelet transform analysis of ZnO, ZnPc, Zn(OH)_2_, and Aga‐PDA/Zn^II^. (i) SEM images showing the surface structure of Aga‐PDA/Zn^II^ after incubation in (i1) NaCl, (i2) urea, (i3) EDTA, or (i4) Tween 20 solution. (j) Percentage of zinc ion loss of Aga‐PDA/Zn^II^ after incubation in NaCl, urea, EDTA, or Tween 20 solution. (k) UV–vis spectra of supernatants after incubating Aga‐PDA/Zn^II^ in NaCl, urea, EDTA, or Tween 20 solution. (l) Heatmap illustrating the stability of Aga‐PDA/Zn^II^ after incubation in different solutions.

To gain deeper insights into the local coordination environment of zinc ions in the Aga‐PDA/Zn^II^ superstructures, x‐ray absorption fine structure analysis was performed. The x‐ray absorption near‐edge structure spectra (Figure 2b) showed that the Zn K‐edge in Aga‐PDA/Zn^II^ differed significantly from that of metallic Zn foil, but closely matched those of ZnO and Zn(OH)_2_, indicating that Zn within these superstructures is predominantly in the +2 oxidation state. Therefore, we anticipated that the local coordination environment of Zn^II^ in Aga‐PDA/Zn^II^ can be preliminarily inferred to be similar to that in ZnO or Zn(OH)_2_. The Fourier‐transform extended x‐ray absorption fine structure (EXAFS) spectrum of Aga‐PDA/Zn^II^ exhibited a major peak at approximately 1.5 Å, which aligned with those of zinc porphyrin (ZnPc) and ZnO, further validating the contribution from Zn─N and Zn─O scattering paths. Curve fitting of the EXAFS data in both *R*‐space and *K*‐space demonstrated that the spectral features of Aga‐PDA/Zn^II^ shared similarities with ZnPc, and the overall shape more closely resembled the disordered or short‐range ordered structure of Zn(OH)_2_ rather than crystalline ZnO (Figure 2c,d). This suggests that the Aga‐PDA/Zn^II^ superstructures contain mixed Zn─O and Zn─N coordination, as well as limited disordered or short‐range ordering. These findings were further supported by the wavelet transform analysis shown in Figures 2e–h and S7. Specifically, a pronounced peak was observed for Aga‐PDA/Zn^II^ in the region of *R* ≈ 1.0–2.0 Å and *K* ≈ 1.0–8.0 Å^−1^, indicative of first‐shell scattering from light atoms. These spectral features align with those associated with oxygen‐ and nitrogen‐based coordination motifs, hence confirming the coexistence of Zn─O and Zn─N bonds. In addition, a less dominant second‐shell signal attributed to Zn─Zn scattering was observed in the regions of *R* ≈ 2.5–3.0 Å and *K* ≈ 1.0–9.0 Å^−1^, with significantly lower intensity and sharpness compared to those of pure ZnO. Notably, the Zn─Zn scattering signal distribution observed in Aga‐PDA/Zn^II^ was distinct from that of Zn foil, Zn(OH)_2_, or ZnPc [33]. These observations collectively suggest the presence of limited Zn─Zn aggregation or short‐range order, rather than the formation of a crystalline ZnO phase. EXAFS fitting results showed that the Zn─O/N bond length in Aga‐PDA/Zn^II^ was approximately 1.99 Å, with a total coordination number of around 6, consistent with that of Zn(OH)_2_. However, this bond length is shorter than the typical Zn─OH bond (2.07 Å) in Zn(OH)_2_, but resembles the Zn─O bond length in ZnO (1.96 Å) and the Zn─N bond length in ZnPc (2.00 Å) (Table S1). Therefore, we hypothesize that most Zn centers are coordinated by a combination of oxygen and nitrogen atoms, resulting in a mixed O*_x_* N*ᵧ* coordination environment, where *x* + *y* ≤ 6. This structure is distinct from the typical tetrahedral Zn─O_4_ configuration in ZnO and the fully hydroxyl‐coordinated octahedral Zn─(OH)_6_ structure in Zn(OH)_2_. Instead, Zn^II^ coordinates with deprotonated hydroxyl groups (─O^−^) or adjacent oxygen atoms on the PDA backbone, forming shorter Zn─O bonds with partially covalent character (1.96 Å, similar to ZnO). Zn^II^ also coordinates with nitrogen atoms from amino (─NH_2_) or imino (═NH) groups on PDA. This dual coordinating mechanism enhanced the overall cross‐linking density and structural stability of PDA/Zn^II^.

### 2.3 Physicochemical Properties of Aga‐PDA/Zn^II^ Superstructures

To investigate the primary interactions responsible for governing the stability of Aga‐PDA/Zn^II^ superstructures, Aga‐PDA/Zn^II^ were incubated with four types of bond‐disrupting agents (100 mM NaCl, 100 mM urea, 100 mM ethylenediaminetetraacetic acid (EDTA), or 100 mM Tween 20). The superstructures were characterized before and after incubation by SEM, ICP‐MS, and UV–vis spectroscopy. NaCl serves as an electrostatic competitor by disrupting ionic interactions. Urea acts as a hydrogen bond competitor by forming strong hydrogen bonds with hydrogen‐donating/accepting sites. EDTA, a strong metal chelator, can dissociate metal–ligand coordination bonds. Tween 20 acts as a competitor for hydrophobic interactions by disrupting nonpolar interactions [34]. SEM observations revealed that treatment with urea or NaCl led to negligible changes in the surface morphology of Aga‐PDA/Zn^II^. In contrast, incubation with EDTA or Tween 20 resulted in disassembly of the PDA/Zn^II^ networks and exfoliation of the microspheres (Figure 2i), suggesting that metal coordination and hydrophobic interactions are the dominant interactions in stabilizing the superstructures. These observations were supported by ICP‐MS findings that demonstrated that incubation with EDTA and Tween 20 led to > 99% and approximately 88% Zn loss from these superstructures, respectively, whereas incubation with urea or NaCl induced < 10% Zn loss (Figures 2j and S8). UV–vis spectroscopy analysis of the superstructures following incubation with urea and NaCl revealed a minor absorption peak at ∼280 nm, which is likely attributed to the release of PDA, resulting from the partial disruption of electrostatic interactions and hydrogen bonding. In contrast, the UV–vis spectrum of the EDTA‐incubated solution displayed typical PDA characteristic absorption peaks at 280 and 330 nm along with broad absorption across the entire wavelength range, confirming the disruption of Zn^II–^PDA coordination. Incubation with Tween 20 led to a similar broad absorption, suggesting the disassembly of the PDA/Zn^II^ networks through competitive hydrophobic interactions (Figure 2k). In summary, the Aga‐PDA/ Zn^II^ superstructures remained relatively stable in NaCl and urea solutions, whereas they showed a tendency to disassemble upon exposure to EDTA and Tween 20 solutions (Figure 2l). Therefore, these results collectively suggest that these superstructures are primarily stabilized by coordination and hydrophobic interactions.

### 2.4 Characterization and Molecular Docking of Bioactive Superstructures

The Aga‐PDA/Zn^II^ superstructures were made bioactive simply through surface modification with HSA (Scheme 1a), forming HAP/Zn^II^ bioactive superstructures. As shown in Figure 3a, HSA modification resulted in a shift of the ζ‐potential from −5.4 to −9.0 mV. Furthermore, x‐ray photoelectron spectroscopy analysis showed an increased percentage of N and O on the surface of HAP/Zn^II^ relative to the contents in Aga‐PDA/Zn^II^ (Table S2), which can be attributed to the abundant amino, carboxyl, and hydroxyl groups in HSA molecules. Analysis of the high‐resolution C 1s spectra (Figure 3b,c and Table S3) indicates that the proportion of C─C/C─H bonds decreased from 40.6% to 31.0% upon HSA modification, while the proportions of C─N bonds and C═O bonds respectively increased from 16.4% to 26.7% and from 13.9% to 20.9%. These increases are due to the abundant number of amide groups present in HSA. In contrast, signals of carboxyl groups from the underlying PDA were partially attenuated after HSA modification, decreasing from 11.0% to 3.8%, further indicating coating of HSA on the substrate.

**FIGURE 3:**
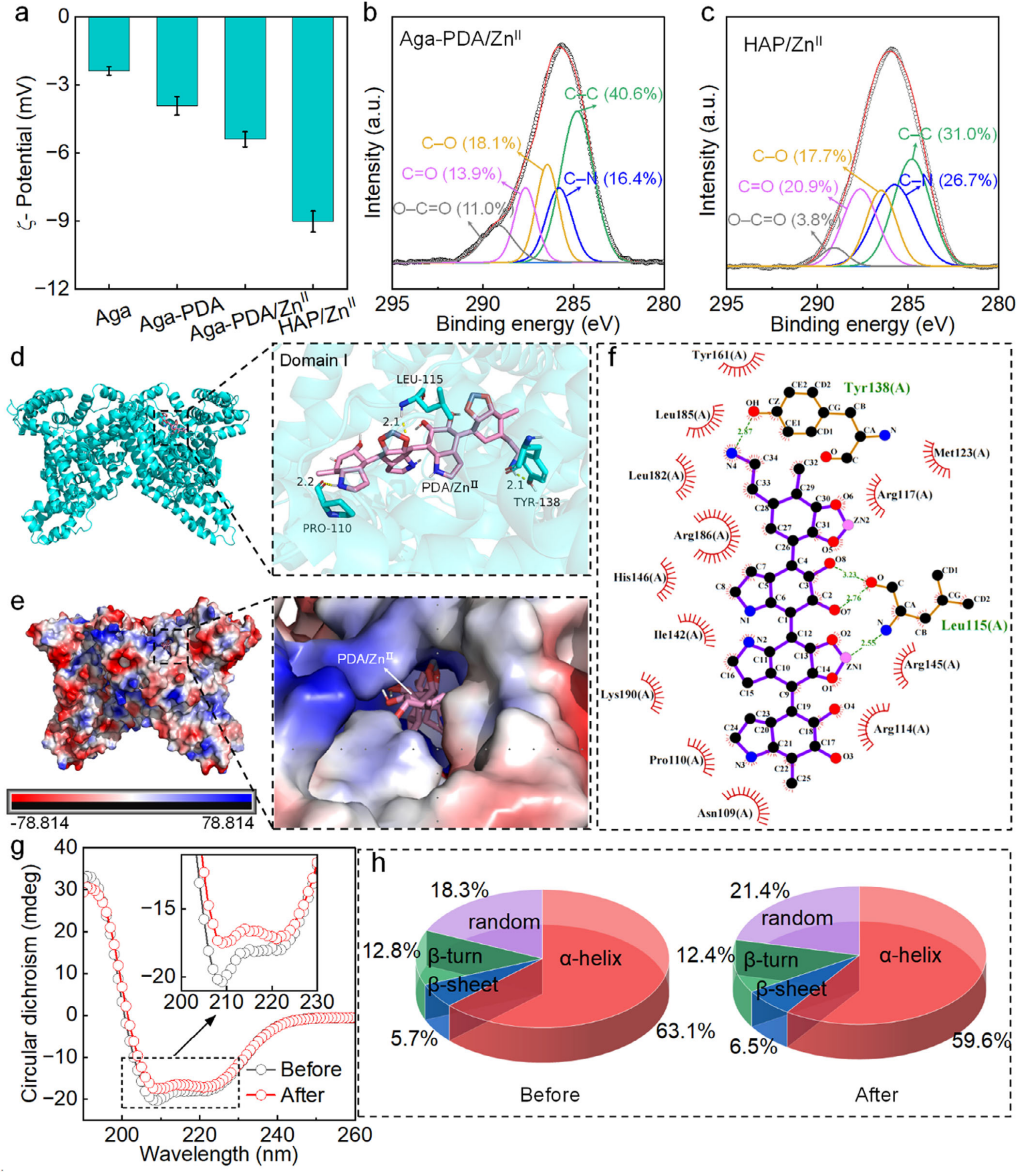
Characterization of Aga‐PDA/Zn^II^ superstructures modified with HSA to form bioactive superstructures (HAP/Zn^II^) and molecular docking results. (a) ξ‐Potential of Aga, Aga‐PDA, Aga‐PDA/Zn^II^, and HAP/Zn^II^. X‐ray photoelectron spectroscopy spectra of (b) Aga‐PDA/Zn^II^ and (c) HAP/Zn^II^. (d) Schematic diagram of the binding interface interactions in the molecular docking complex formed between HSA and the PDA/Zn^II^. (e) Electrostatic potential distribution on the protein surface of the HSA docking complex with the PDA/Zn^II^ molecule; color indicates charge distribution: red regions—negatively charged (acidic residues, e.g., Glu, Asp); blue regions—positively charged (basic residues, e.g., Lys, Arg, His); white regions—neutral or hydrophobic regions. (f) 2D interaction diagram of the molecule–protein docking complex (LigPlot+ analysis); hydrogen bonds (green dashed lines), hydrophobic interactions (red arcs), and adjacent polar residues are shown. (g) CD spectra and (h) secondary structure composition of HSA before and after modification of Aga‐PDA/Zn^II^ to form HAP/Zn^II^.

We then investigated the loading capacity of HSA using fluorescein isothiocyanate‐labeled HSA. The Aga‐PDA/ Zn^II^ superstructures exhibited an HSA adsorption capacity of 227.7 mg g^−1^, which is approximately 8.0‐fold and 3.5‐fold higher than those of Aga (28.8 mg g^−1^) and Aga‐PDA (65.5 mg g^−1^), respectively (Figure S9). The improved loading capacity is primarily attributed to the nanoporous network on the surface of the PDA/Zn^II^ microspheres, which provides numerous binding sites for HSA.

To further elucidate the mechanism underlying the interactions between HSA and PDA/Zn^II^, molecular docking was performed using the minimal structural unit of PDA/Zn^II^ as the ligand and HSA as the receptor. The binding energy between PDA/Zn^II^ and HSA was −9.9 kcal mol^−1^ (Figure S10 and Table S4), suggesting a high tendency for complex formation under physiological conditions. Interface analysis revealed that PDA/Zn^II^ primarily bind to a region adjacent to Domain I of HSA (Figure 3d). Hydrogen bonding between PDA/Zn^II^ and amino acid residues, such as PRO‐110, LEU‐115, and TYR‐138, enhanced the affinity and stabilized the overall conformation of the PDA/Zn^II^ and HSA complex (Figure 3d). As shown by the electrostatic potential surface of the HSA and PDA/Zn^II^ complex (Figure 3e), the negatively charged PDA/Zn^II^ preferentially interacts with positively charged regions (blue) within the HSA binding pocket through electrostatic attraction, while additional stabilization arises from interactions with neutral or hydrophobic areas (white) via hydrophobic interactions. The interactions between PDA/Zn^II^ and HSA residues were further visualized in the 2D interaction diagram in Figure 3f. Specifically, PDA/Zn^II^ is embedded into a mixed hydrophobic/polar pocket composed of residues Asn109, Pro110, Lys190, Leu115, Met123, Tyr138/Tyr161, Ile142, His146, Arg186/Arg117/Arg114/Arg145, and Leu182/Leu185, spanning approximately residues 109–190. The major interactions include hydrogen bonds (green dashed lines), hydrophobic contacts (red semicircles), and proximity effects among polar residues. Notably, the distance between Zn and the nitrogen atom of Leu115 is 2.55 Å, which is shorter than typical van der Waals contact distances but longer than that of a typical Zn─N coordination bond (2.0–2.2 Å) [35]. This suggests that the local structural rearrangement is driven by electrostatic attraction of Zn^II^ toward nearby negatively charged or polar groups, instigating the approach of Leu115 N without effectively forming a coordination bond. Furthermore, Zn^II^ did not exhibit short‐range coordination with common donor residues in HSA (such as His, Asp, or Glu) [36, 37], which may assist with preserving the secondary structure of HSA and maintaining its biological activity.

The secondary structural changes of HSA play a crucial role in its anticoagulant activity and biocompatibility. Maintaining a specific proportion of secondary structures, particularly the α‐helix, is essential for preserving its biological function. Circular dichroism (CD) spectroscopy was employed to analyze variations in the secondary structure of HSA before and after modification of Aga‐PDA/Zn^II^ (Figure 3g). The intensity of the negative peaks at 208 and 222 nm in the far‐UV CD spectrum of HSA decreased slightly after modification, indicating a minor reduction in α‐helix content. Quantitative analysis of the secondary structure revealed that, prior to modification, HSA contained 63.1% α‐helix, 5.7% β‐sheet, 12.8% β‐turn, and 18.3% random coil. After modification, the α‐helix content remained high at 59.6%, whereas the contents of the β‐sheet and random coil structures slightly increased to 6.5% and 21.4%, respectively (Figure 3h). These results collectively suggest that HSA largely retains a high level of biological activity following immobilization, which is envisioned to enhance both the biocompatibility and anticoagulant performance of the superstructures. Notably, HAP/Zn^II^ remained stable after 1 day incubation under simulated clinically relevant conditions (i.e., 37°C with shaking at 150 rpm; Figure S11), indicating its potential applicability under dynamic fluidic conditions associated with hemoperfusion.

### 2.5 Bilirubin Adsorption and Removal Performance

To simulate the physiological pH of human blood, bilirubin solutions were prepared in PBS (pH = 7.4). The time‐dependent adsorption behaviors of Aga, Aga‐PDA, Aga‐PDA/Zn^II^, and HAP/Zn^II^ toward bilirubin were first investigated. Both Aga‐PDA/Zn^II^ and HAP/Zn^II^ exhibited rapid bilirubin adsorption and reached equilibrium within 60 min, with an adsorption capacity of 212 mg g^−1^ (Figure 4a). In contrast, Aga and Aga‐PDA showed significantly slower adsorption rates and capacities (24.0 and 65.9 mg g^−1^, respectively). Bilirubin removal efficiency of Aga‐PDA/Zn^II^ and HAP/Zn^II^ exceeded 85.0%, whereas those of Aga and Aga‐PDA were limited to 9.6% and 26.3%, respectively (Figures 4b and S12). The high bilirubin adsorption capacity and removal efficiency of Aga‐PDA/Zn^II^ and HAP/Zn^II^ are likely attributed to the abundant accessible adsorption sites provided by their hierarchical nanonetwork.

**FIGURE 4:**
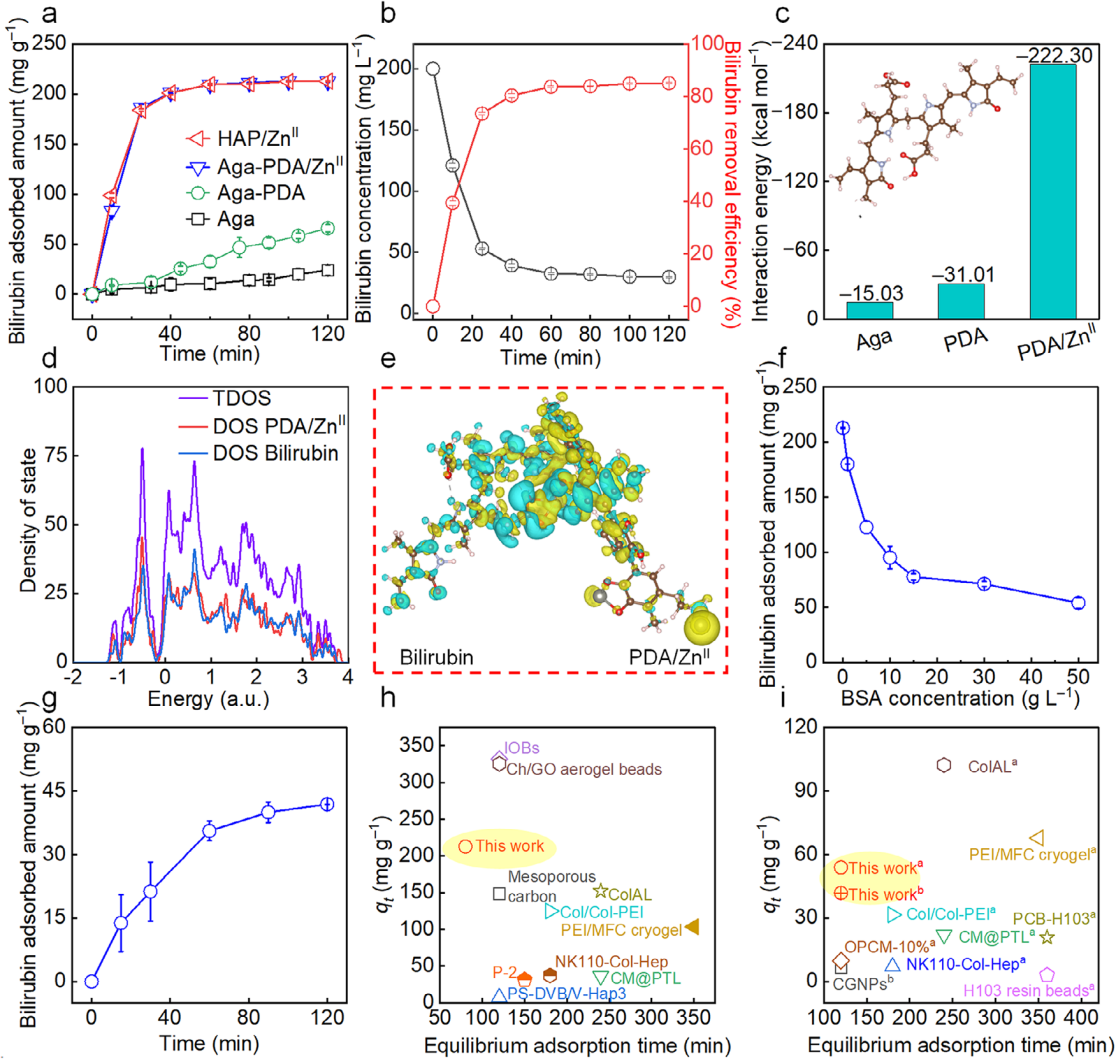
Bilirubin removal performance of the bioactive superstructures. (a) Bilirubin adsorption capacities of Aga, Aga‐PDA, Aga‐PDA/Zn^II^, and HAP/Zn^II^ as a function of contact time. (b) Residual bilirubin concentration and bilirubin removal efficiency of HAP/Zn^II^ bioactive superstructures versus contact time. (c) Binding energies of interactions between bilirubin and Aga, PDA, or PDA/Zn^II^ calculated by density functional theory. Inset shows the molecular structure of bilirubin. (d) DOS diagrams of the interactions between bilirubin and PDA/Zn^II^. (e) Charge density difference map of the interactions between bilirubin and PDA/Zn^II^; yellow and blue regions indicate electron density accumulation and depletion, respectively (isosurface value = 0.0015). (f) Bilirubin adsorption capacity of HAP/Zn^II^ in the presence of BSA at varying concentrations. (g) Bilirubin adsorption capacity of HAP/Zn^II^ in simulated plasma as a function of contact time (initial concentration of bilirubin = 200 mg L^−1^, dosage of HAP/Zn^II^ = 0.8 g L^−1^). (h) Comparison of the bilirubin adsorption capacities of HAP/Zn^II^ and other reported bilirubin adsorbents at a given equilibrium time. (i) Comparison of the bilirubin adsorption capacities of HAP/Zn^II^ and other reported adsorbents at equilibrium time and under different conditions: a—albumin‐containing condition; b—simulated plasma condition (50 g L^−1^ BSA and 9 g L^−1^ NaCl). Details of the reported adsorbents in (h) and (i) are provided in Table S5. In (a,b,f,g), data are presented as mean ± standard deviation (SD) (*n* ≥ 3).

To elucidate the adsorption mechanism, density functional theory calculations were performed to evaluate the interaction energies between bilirubin and Aga, PDA, or PDA/Zn^II^ (Figures 4c and S13). The interaction energy between bilirubin and Aga was only −15.03 kcal mol^−1^, consistent with its limited bilirubin adsorption capacity. In contrast, bilirubin binding to PDA exhibited a stronger interaction energy (−31.01 kcal mol^−1^), which was further enhanced upon Zn^II^ coordination, reaching −222.30 kcal mol^−1^ for PDA/Zn^II^ (Figure 4c). These results indicate that Zn^II^ coordination significantly strengthens bilirubin binding and stabilizes bilirubin within the PDA/Zn^II^ network. Likewise, the electronic density of states (DOS) peaks of PDA/Zn^II^ and bilirubin displayed significant overlap in the energy range of –2–4 eV (Figure 4d), suggesting orbital hybridization between the two components and strong interactions. Differential charge density mapping showed pronounced electron accumulation around Zn^II^ and its coordination sphere, accompanied by electron depletion around the carboxyl and carbonyl oxygen atoms of bilirubin (Figure 4e), indicating electron transfer from bilirubin to Zn^II^ and the formation of coordination bonding. Moreover, an independent gradient model based on Hirshfeld partition analysis revealed the coexistence of blue and green isosurfaces (Figure S14), indicating the combined contributions of multiple noncovalent interactions, including hydrogen bonding, *π*–*π* stacking, and van der Waals forces in stabilizing the complex. These results collectively suggest complementary contributions of Aga, PDA, and Zn^II^ in bilirubin adsorption. Aga may serve as a stable scaffold for the PDA/Zn^II^ network, while facilitating mass transfer during adsorption [38, 39]. PDA is likely to interact with bilirubin through multiple supramolecular interactions, including hydrogen bonding, hydrophobic interactions, and *π*–*π* stacking. Zn^II^ provides additional coordination sites for the carboxyl and carbonyl groups of bilirubin, thereby enhancing the overall binding affinity. The synergistic interplay of these interactions establishes a robust bilirubin‐binding network, resulting in efficient bilirubin‐capture capability of the Aga‐PDA/Zn^II^.

The strong bilirubin–PDA/Zn^II^ interaction allows HAP/Zn^II^ to maintain efficient adsorption under competitive conditions. In a mixture of bilirubin and bovine serum albumin (BSA), the removal rate of bilirubin gradually decreased with increasing BSA concentrations due to bilirubin–BSA binding; however, even at a BSA concentration of 50 g L^−1^, HAP/Zn^II^ maintained a high bilirubin adsorption capacity (53.9 mg g^−1^ within 120 min; Figure 4f). Under simulated plasma conditions, HAP/Zn^II^ enabled a bilirubin adsorption capacity of 41.9 mg g^−1^ within 120 min (Figure 4g). Furthermore, HAP/Zn^II^ and Aga‐PDA/Zn^II^ showed comparable bilirubin adsorption capacities (Figure S15), suggesting that bilirubin adsorption is primarily governed by PDA/Zn^II^‐mediated interactions. Notably, HAP/Zn^II^ outperformed most reported bilirubin adsorbents in terms of both adsorption rate and capacity in both pure bilirubin solution and protein‐rich media (Figure 4h,i and Table S5).

### 2.6 Antibacterial Activity of HAP/Zn^II^

For their application in hemoperfusion, it is essential that adsorbents display antibacterial activity [40]. To evaluate the antibacterial capability of the prepared superstructures, *Escherichia coli* (*E. coli*) and *Staphylococcus aureus* (*S. aureus*), which are typical Gram‐negative and Gram‐positive bacteria, respectively, were cocultured with Aga, Aga‐PDA, Aga‐PDA/Zn^II^, or HAP/Zn^II^ in Luria–Bertani media under dynamic conditions (i.e., 37°C with agitation at 150 rpm) that mimic the temperature and flow conditions employed during hemoperfusion. During incubation, the optical density (OD) of the bacterial suspensions at 600 nm was monitored using a microplate reader. For both *E. coli* and *S. aureus*, the bare (bacterial suspension without samples), Aga, and Aga‐PDA groups exhibited a significant increase in OD over 24 h, indicating bacterial proliferation (Figure 5a,b). In contrast, the OD values of suspensions cocultured with Aga‐PDA/Zn^II^ or HAP/Zn^II^ showed no apparent changes over time, suggesting that the adsorbents comprising PDA/Zn^II^ effectively inhibit the growth of both bacterial species. To further confirm bacterial inactivation, *E. coli* and *S. aureus* suspensions exposed to Aga, Aga‐PDA, Aga‐PDA/ Zn^II^, or HAP/ Zn^II^ were plated on agar for colony formation. As shown in Figures 5d and S16, dense bacterial colonies were observed in the Aga and Aga‐PDA groups, whereas almost no colonies were visible in the Aga‐PDA/Zn^II^ and HAP/Zn^II^ groups, highlighting the effective inhibition of bacterial proliferation. Furthermore, the bacterial killing rates of the Aga group were only 14.7% for *E. coli* and 24.1% for *S. aureus*, indicating minimal antibacterial activity (Figure 5c). Aga‐PDA displayed a slightly higher killing rate of *E. coli* and *S. aureus* of 29.8% and 27.4%, respectively. Notably, Aga‐PDA/ Zn^II^ and HAP/Zn^II^ displayed significantly improved killing rates of >99.9% for both *E. coli* and *S. aureus*, further showcasing the broad‐spectrum antibacterial properties of these superstructures.

**FIGURE 5:**
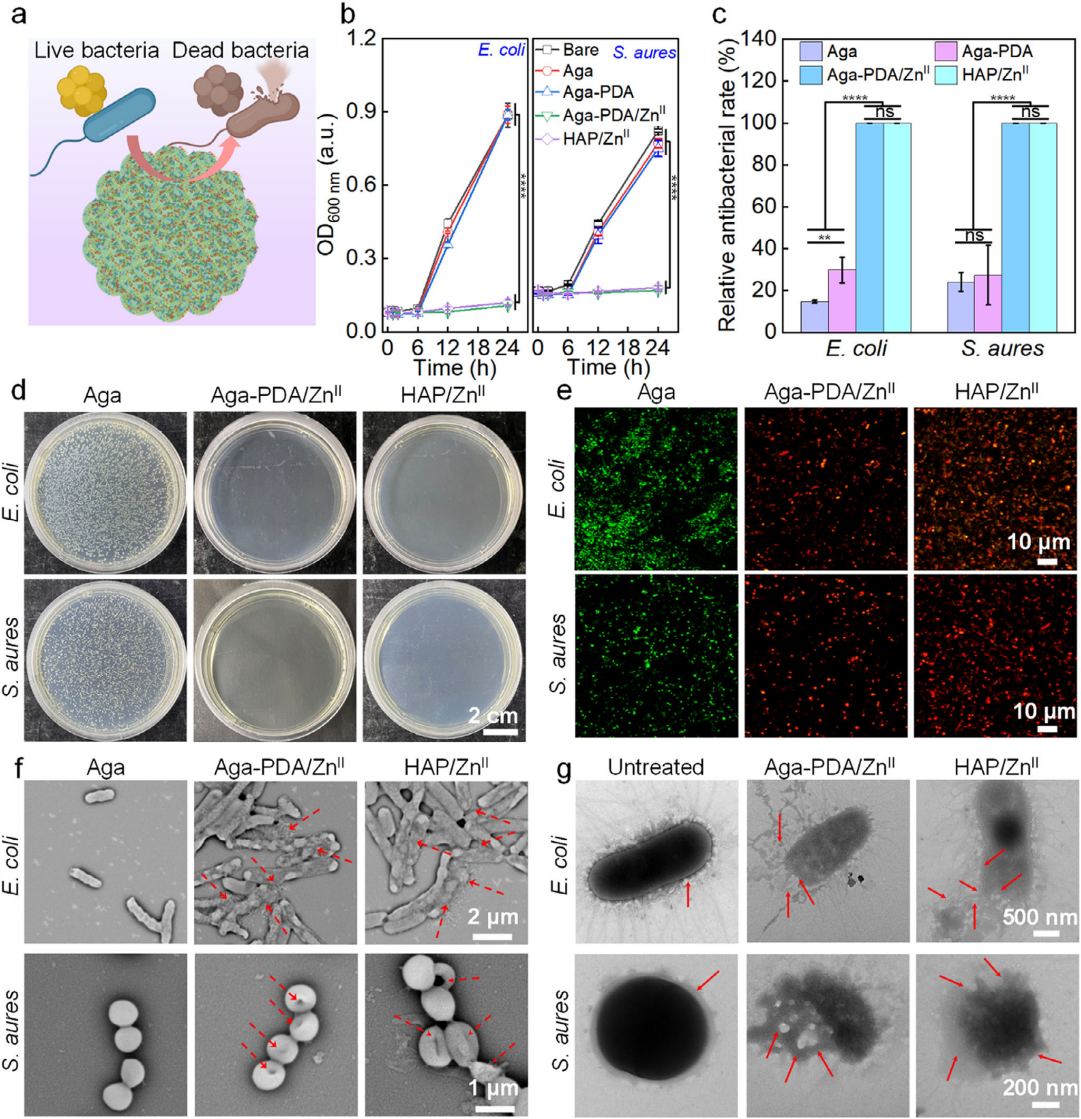
Antibacterial performance of the bioactive superstructures. (a) Schematic diagram of the antibacterial activity of HAP/Zn^II^ bioactive superstructures. (b) Growth curves of *E. coli* and *S. aureus* after co‐culture for 24 h with Aga, Aga‐PDA, Aga‐PDA/Zn^II^, or HAP/Zn^II^. (c) Relative antibacterial rates of Aga, Aga‐PDA, Aga‐PDA/Zn^II^, and HAP/Zn^II^ against *E. coli* and *S. aureus*. (d) Optical images showing the growth of *E. coli* and *S. aureus* on agar plates after co‐culture with Aga, Aga‐PDA/Zn^II^, or HAP/Zn^II^. (e) Fluorescence microscopy images of live/dead stained *E. coli* and *S. aureus* after contact‐killing with Aga, Aga‐PDA/Zn^II^, or HAP/Zn^II^. (f) SEM images of *E. coli* and *S. aureus* fixed on slides after sterilization via contact with Aga, Aga‐PDA/Zn^II^, or HAP/Zn^II^. (g) TEM images of *E. coli* and *S. aureus* before and after contact‐killing with Aga‐PDA/Zn^II^ or HAP/Zn^II^. In (b) and (c), data are presented as mean ± SD (*n* ≥ 3). One‐way analysis of variance: ns, not significantly different, **p < 0.0021, ****p < 0.0001.

The antibacterial activity of these materials was further evaluated through a viability assay. Live bacteria emit green fluorescence, whereas dead bacteria emit red fluorescence. As observed from Figures 5e and S17, the untreated (control) group displayed intense green emission with negligible red signal, suggesting high bacterial viability. In contrast, pronounced red fluorescence was observed upon bacterial exposure to Aga‐PDA/Zn^II^ and HAP/Zn^II^, indicating rupture of bacterial membranes [27, 28]. To further investigate the mechanism underlying the antibacterial performance of PDA/Zn^II^, bacterial morphology after contact with Aga, Aga‐PDA, Aga‐PDA/Zn^II^, and HAP/Zn^II^ was examined via the glass coverslip adhesion method (Figures 5f and S18). In the untreated group (untreated bacterial suspension), *E. coli* displayed a typical rod shape, while *S. aureus* displayed smooth spherical structures. After treatment with Aga or Aga‐PDA, most bacteria retained their overall morphology. In contrast, surface depressions and membrane disruption were observed in most *E. coli* cells, accompanied by evident structural deformation in *S. aureus* cells subjected to treatment with Aga‐PDA/Zn^II^ or HAP/Zn^II^, highlighting their antibacterial activity (Figure 5f). TEM further revealed structural and organelle damage in bacteria treated with Aga‐PDA/Zn^II^ or HAP/Zn^II^ (Figures 5g and S19). In the untreated group, both *E. coli* and *S. aureus* displayed intact and well‐defined cell walls and membranes. However, treatment with Aga‐PDA/Zn^II^ or HAP/Zn^II^ resulted in severe cell wall disruption and substantial cytoplasmic leakage in nearly all bacterial cells, indicating structural destruction of bacteria. In addition, we evaluated the Zn^II^ release profiles of both Aga‐PDA/Zn^II^ and HAP/Zn^II^, as the release of Zn^II^ may also contribute to antibacterial effects. As shown in Figure S20, both Aga‐PDA/Zn^II^ and HAP/Zn^II^ exhibited sustained Zn^II^ release over 15 days with less than 2% Zn loss after the 1st day. These results collectively suggest that the antibacterial activity arises from the combined effects of the localized Zn^II^ and contact‐killing mediated by the hierarchical composite architecture of these superstructures. Notably, the antibacterial performance of Aga‐PDA/Zn^II^ and HAP/Zn^II^ under dynamic physiologically relevant culture conditions (Figure S21) was comparable to that achieved using Luria–Bertani media (Figure 5), further highlighting the potential of these materials for blood‐contacting and hemoperfusion‐related applications.

### 2.7 Biocompatibility and Anticoagulant Properties of HAP/Zn^II^

The biocompatibility of HAP/Zn^II^ was evaluated in terms of hemolysis rate and cytotoxicity. The HAP/Zn^II^ bioactive superstructures displayed a significantly low hemolysis rate of only 0.16%, which is considerably below the commonly accepted 5% threshold for hemolytic materials and within the nonhemolytic range (< 2%) [41, 42], indicating that HAP/Zn^II^ induced a negligible burst of red blood cells (Figure 6a). To further assess cytocompatibility, L929 mouse fibroblasts were treated with Aga, Aga‐PDA, Aga‐PDA/Zn^II^, or HAP/Zn^II^ at concentrations of 250 and 500 µg mL^−1^ for 24, 48, and 72 h. Cell viability remained over 80% across all samples at 250 µg mL^−1^ (Figure S22). When the concentration increased to 500 µg mL^−1^, the cell viability of Aga‐PDA/Zn^II^ reduced to ∼70% at 24 h, and further declined to < 5% at 48 and 72 h. This pronounced cytotoxicity is likely attributed to the sustained release of Zn^II^ from Aga‐PDA/Zn^II^. In contrast, cells treated with HAP/Zn^II^ retained over 80% cell viability over the 72 h incubation period studied (Figure 6b), likely attributed to the significantly lower Zn^II^ release from HAP/Zn^II^ (0.38 mg g^−1^) than from Aga‐PDA/Zn^II^ (2.27 mg g^−1^) at 72 h (Figure S20a,b). We anticipate that the additional coordination between HSA and PDA/Zn^II^ may further stabilize these superstructures, thereby inhibiting the release of Zn^II^ to the cell media. These results collectively demonstrate the biocompatibility of HAP/Zn^II^.

**FIGURE 6:**
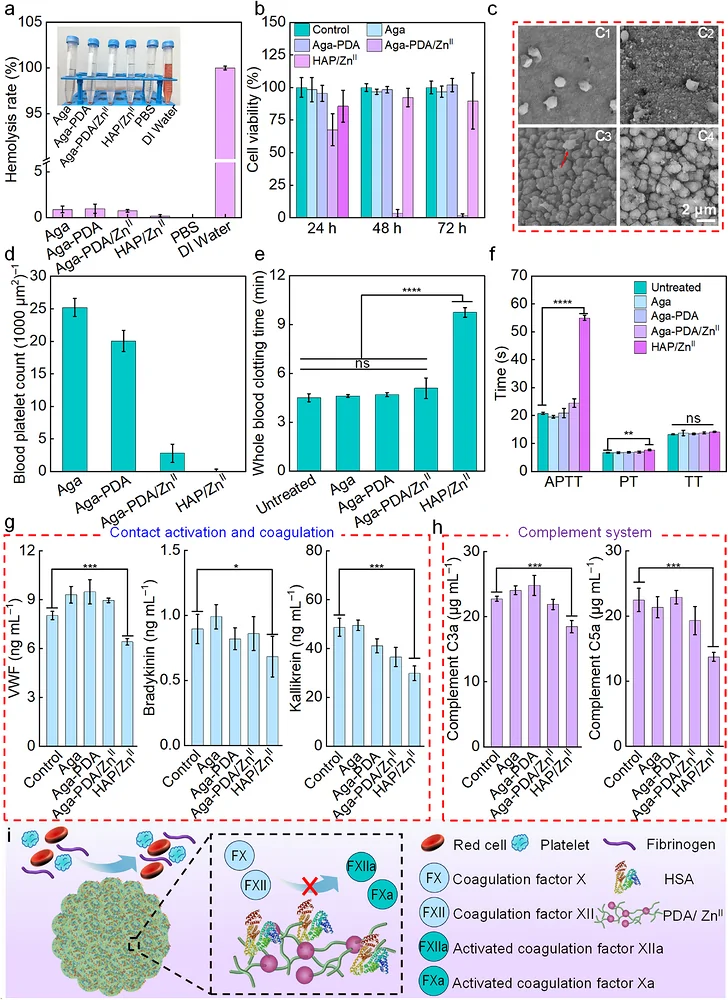
Biocompatibility and anticoagulant properties of the bioactive superstructures. (a) Hemolysis rate of Aga, Aga‐PDA, Aga‐PDA/Zn^II^, and HAP/Zn^II^; inset shows a photograph of the red blood cell suspensions after coincubation and centrifugation. Intact cells pelletized with a clear supernatant, whereas a red‐colored supernatant indicates hemolysis (i.e., burst of red blood cells). DI water: deionized water. (b) Viability of L929 cells after incubation with Aga, Aga‐PDA, Aga‐PDA/Zn^II^, or HAP/Zn^II^ for 24, 48, and 72 h (sample concentration: 500 µg mL^−1^). (c) SEM images showing platelet adhesion on the surfaces of (c1) Aga, (c2) Aga‐PDA, (c3) Aga‐PDA/Zn^II^, and (c4) HAP/Zn^II^. (d) Corresponding platelet count obtained from the SEM images. (e) Whole blood coagulation time after incubation with Aga, Aga‐PDA, Aga‐PDA/Zn^II^, or HAP/Zn^II^. Untreated: whole blood without samples. (f) Activated partial thromboplastin time (APTT), prothrombin time (PT), and thrombin time (TT) values of Aga, Aga‐PDA, Aga‐PDA/Zn^II^, and HAP/Zn^II^. Untreated: platelet‐poor plasma without samples. (g) Levels of contact activation‐related factors bradykinin, kallikrein, and coagulation‐related factor VWF. Control: plasma without samples. (h) Levels of complement system‐related factors (C3a and C5a) in plasma after incubation with Aga, Aga‐PDA, Aga‐PDA/Zn^II^, or HAP/Zn^II^. Control: plasma without samples. (i) Schematic of the anticoagulant mechanism of the bioactive superstructures. In (a, b, d–f, g, h), data are presented as mean ± SD (*n* ≥ 3); one‐way analysis of variance: ns, not significantly different, *p < 0.0332, **p < 0.0021, ***p < 0.0002, and ****p < 0.0001.

Next, we incubated Aga, Aga‐PDA, Aga‐ PDA/Zn^II^, and HAP/Zn^II^ with whole blood followed by complete blood count analyses. Apart from the glass bead positive control, which induced a significant reduction in white and red blood cell counts compared with the saline control, all tested materials caused a negligible change in red and white blood cell numbers (Figure S23a,b), further suggesting the hemocompatibility of these materials. Platelet count, a key indicator of coagulation function, also provides insight into platelet activation and consumption [43]. Incubation of blood with Aga or Aga‐PDA resulted in a reduction in platelet count, whereas platelet count following incubation with HAP/Zn^II^ remained comparable to the control (i.e., blood incubated with saline) (Figure S23c). SEM observations further confirmed these results. A large number of adhered and partially activated platelets were visible on the surfaces of Aga and Aga‐PDA, with approximately 25 and 20 platelets per 1000 µm^2^, respectively. In contrast, platelet adhesion on Aga‐PDA/Zn^II^ was substantially reduced, with only about 3 platelets per 1000 µm^2^, and was negligible on HAP/Zn^II^ (Figure 6c,d). The presence of PDA/Zn^II^ on the surface of both Aga‐PDA/Zn^II^ and HAP/Zn^II^ might enhance surface hydrophilicity and reduce fibrinogen adsorption [27, 44], thereby reducing platelet adhesion. Furthermore, HSA on the surface of HAP/Zn^II^ might provide an additional reduction in fibrinogen adsorption and subsequent platelet adhesion and activation [21]. These findings indicate that HAP/Zn^II^ exhibits excellent anti‐platelet adhesion performance, which likely contributes to its enhanced anticoagulant capability.

The anticoagulant properties of HAP/Zn^II^ were further evaluated using whole‐blood clotting assays. The clotting times of the Aga, Aga‐PDA, and Aga‐PDA/Zn^II^ groups were 4.6, 4.7, and 5.1 min, respectively, showing no significant improvement compared with the untreated control at 4.5 min. We anticipate that, despite reduced fibrinogen adsorption afforded by PDA/Zn^II^, residual fibrinogen and other thrombogenic plasma proteins may still be adsorbed onto the surface of Aga‐PDA/Zn^II^, leading to the activation of the coagulation cascade. In contrast, HAP/Zn^II^ prolonged the clotting time from 4.5 to 9.8 min (Figure 6e). Furthermore, Aga‐PDA/Zn^II^ demonstrated an activated partial thromboplastin time of 20.8 s, which was comparable to those of untreated (platelet‐poor plasma without microspheres), Aga, and Aga‐PDA, indicating limited anticoagulant activity. In contrast, HAP/Zn^II^ extended the activated partial thromboplastin time from 20.8 to 55.0 s and increased the prothrombin time from 6.7 to 7.6 s, while causing negligible changes in thrombin time (Figure 6f). These results collectively indicate that HAP/Zn^II^ enhances anticoagulant performance, primarily by inhibiting platelet adhesion/activation and modulating the intrinsic coagulation pathway with minimal effects on the extrinsic pathway and fibrin formation [45]. To obtain further mechanistic insight into the anticoagulant properties of HAP/Zn^II^, we then evaluated the secretion levels of contact activation‐related factors (i.e., bradykinin and kallikrein), coagulation‐related factor (i.e., von Willebrand factor), and complement activation‐related factors (i.e., C3a and C5a) following treatment with HAP/Zn^II^. Figures 6g,h and S24 show the secretion levels of von Willebrand factor (VWF), bradykinin, kallikrein, complement components C3a and C5a, and interleukin‐6 (IL‐6) and IL‐8 in rabbit plasma after incubation with different materials. Elevated VWF expression is typically associated with thrombus formation, as VWF acts as the main carrier of factor VIII (FVIII), which serves as a cofactor for FIXa to accelerate the conversion of FX to FXa and promote thrombin generation [46]. Compared with the neat plasma (control group), the VWF expression in the HAP/Zn^II^ group decreased by 20.1%, suggesting that HAP/Zn^II^ suppressed coagulation by inhibiting FX activation (Figure 6g). During the intrinsic coagulation process, FXII is converted to its active form FXIIa, which activates prekallikrein into kallikrein. Kallikrein then cleaves high‐molecular‐weight kininogen to release bradykinin, and through positive feedback, further amplifies FXII activation [47, 48]. Compared with neat plasma, bradykinin and kallikrein levels in the HAP/Zn^II^ group decreased from 0.90 to 0.68 ng mL^−1^ and from 48.71 to 29.85 ng mL^−1^, respectively (Figure 6g), suggesting that HAP/Zn^II^ effectively inhibits the conversion of FXII to FXIIa, thereby suppressing initiation of the intrinsic coagulation cascade. The complement system is typically activated when blood comes into contact with foreign surfaces, which are often linked to innate immune and pro‐inflammatory responses [49]. Incubation with Aga, Aga‐PDA, or Aga‐PDA/Zn^II^ triggered negligible differences in C3a and C5a levels compared with neat plasma, whereas incubation with HAP/Zn^II^ resulted in decreases of 18.9% and 39.0% in C3a and C5a, respectively, indicating the anti‐inflammatory properties of HAP/Zn^II^ (Figure 6h). This anti‐inflammatory function is likely mediated by HSA on the HAP/Zn^II^ surface, which impedes the adsorption of complement fragments C3a and C5a [49, 50], while PDA further contributes to maintaining stable IL‐6 and IL‐8 levels (Figure S24), thereby minimizing the inflammatory response induced by the materials. Overall, the bioactive superstructures (i.e., HAP/Zn^II^) obtained by HSA modification exhibited excellent biocompatibility and anticoagulant performance. As illustrated in Figures 6g and S25, the anticoagulant mechanism of HAP/Zn^II^ involves the inhibition of platelet adhesion and activation by the surface‐immobilized HSA, while simultaneously reducing the adsorption and activation of coagulation factors FX and FXII into FXa and FXIIa. This process ultimately suppresses fibrin formation in the final stage of coagulation, thereby preventing thrombus formation by red blood cells, activated platelets, and fibrin, and achieving a potent anticoagulant effect.

## 3 Conclusion

We have reported the metal–phenolic‐mediated assembly of Aga‐PDA/Zn^II^ superstructures, which display high Young's modulus and mechanical strength. Post‐modification with HSA produced bioactive superstructures (HAP/Zn^II^), wherein the PDA/Zn^II^ network in HAP/Zn^II^ can bind bilirubin through multiple supramolecular interactions, thereby enabling efficient bilirubin removal. Notably, both Aga‐PDA/Zn^II^ and HAP/Zn^II^ exhibited broad‐spectrum antibacterial properties, likely through the localized Zn^II^ release and contact‐killing. Moreover, HAP/Zn^II^ displayed high biocompatibility, inhibition of platelet adhesion and activation, and potent anticoagulant performance. In summary, owing to the tunable superstructure assembly strategy, the use of readily available building blocks, the simple fabrication process, and the integrated advantages demonstrated in toxin adsorption and removal, antibacterial activity, and anticoagulation, these bioactive superstructures are expected to be amenable to further functionalization and scale‐up. We envisage that the combined advantages exhibited by these bioactive superstructures, along with future validation using preclinical models (in vivo hemoperfusion and extracorporeal circulation), will extend their application potential to a wide range of blood purification systems and support their clinical translation.

## Author Contributions

**Po Wang**: conceptualization, data curation, formal analysis, methodology, investigation, validation, writing – original draft, writing – review and editing. **Jingqu Chen**: conceptualization, formal analysis, supervision, validation, writing – review and editing. **Siying Chen**: data curation, formal analysis, methodology, investigation, validation, writing – review and editing. **Binsha Peng**: data curation, formal analysis, methodology, investigation, validation, writing – review and editing. **Zhifeng Zhou**: data curation, formal analysis, methodology, investigation, validation, writing – review and editing. **Ling Zhang**: conceptualization, resources, supervision, validation, writing – review and editing. **Rui Xie**: data curation, formal analysis, methodology, investigation, validation, writing – review and editing. **Xiaojie Ju**: data curation, formal analysis, methodology, investigation, validation, writing – review and editing. **Wei Wang**: data curation, formal analysis, methodology, investigation, validation, writing – review and editing. **Dawei Pan**: data curation, formal analysis, methodology, investigation, validation, writing – review and editing. **Liangyin Chu**: data curation, funding acquisition, project administration, supervision, validation, writing – review and editing. **Frank Caruso**: conceptualization, funding acquisition, project administration, resources, supervision, validation, writing – review and editing. **Zhuang Liu**: conceptualization, funding acquisition, project administration, resources, validation, supervision, writing – review and editing.

## Conflicts of Interest

The authors declare no conflict of interest.

## Supporting information




**Supporting File**: anie73955‐sup‐0001‐SuppMat.pdf.

## Data Availability

The data that support the findings of this study are available from the corresponding author upon reasonable request.
