# Supplementary material for: Assembly of Bioactive Superstructures via Metal–Phenolic Complexation for Blood Purification
Source: Angew Chem Int Ed Engl. 2026 Aug 5;65(40):e2081750. doi: 10.1002/anie.2081750 (PMC13618263; doi:10.1002/anie.2081750)
Supplement: Supplementary file 1 [file ANIE-65-e2081750-s001.pdf]

Supporting Information

Assembly of Bioactive Superstructures via Metal–Phenolic Complexation for Blood Purification

Po Wang,[a],[b] Jingqu Chen,[b] Siying Chen,[a] Binsha Peng,[a] Zhifeng Zhou,[c] Ling Zhang,*[c] Rui Xie,[a] Xiaojie Ju,[a] Wei Wang,[a] Dawei Pan,[a] Liangyin Chu,[a] Frank Caruso,*[b] and Zhuang Liu*[a]

[a] P. Wang, S. Chen, B. Peng, Prof. R. Xie, Prof. X. Ju, Prof. W. Wang, Prof. D. Pan, Prof. L. Chu, Prof. Z. Liu
School of Chemical Engineering
Sichuan University
Chengdu, Sichuan 610065, P. R. China
E-mail: liuz@scu.edu.cn

[b] P. Wang, Dr. J. Chen, Prof. F. Caruso
Department of Chemical Engineering
The University of Melbourne
Parkville, Victoria 3010, Australia
E-mail: fcaruso@unimelb.edu.au

[c] Z. Zhou, Prof. L. Zhang
Department of Nephrology
Institute of Kidney Diseases, West China Hospital of Sichuan University
Chengdu 610041, China
E-mail: zhangling_crrt@163.com

Experimental Section

Materials. Dopamine hydrochloride (DA) was purchased from Sigma-Aldrich (USA). LIVE/DEAD BacLight bacterial viability kit (L7012) was obtained from Invitrogen (USA). Zinc chloride and ammonium hydroxide were purchased from Aladdin (China). *Staphylococcus aureus* (*S. aureus*, American Type Culture Collection 25923) and *Escherichia coli* (*E. coli*, American Type Culture Collection 25922) were purchased from Solarbio (China). The mouse fibroblast cell line L929 was purchased from Procell Life Science & Technology Co., Ltd (China). Ultrapure water was obtained from a Milli-Q system (Millipore, USA). Enzyme-linked immunosorbent assay (ELISA) kits were purchased from Jiangsu Meimian Industrial Co., Ltd. (China). Agarose (Aga) microspheres were provided by Jiangsu Guanhuai Medical Technology Co., Ltd. (China). Human serum albumin (HSA), fluorescein isothiocyanate (FITC)-labeled HSA (FITC-HSA), and bovine serum albumin (BSA) were purchased from Solarbio (China). Phosphate-buffered saline (PBS, pH 7.4) was purchased from Gibco (China). Unless otherwise specified, all other chemical reagents were obtained from Aladdin (China).

Preparation of Aga-PDA/Zn^{II} Superstructures. $ZnCl_2$ (13.63 g) was added to Milli-Q water (50 mL) to obtain a 2 mol L^{-1} Zn^{II} solution. Then, 25% ammonia solution (39 mL) was gradually added to the Zn^{II} solution (50 mL) until the initially formed white flocculent precipitate just disappeared, yielding a zinc–ammonia complex. Based on zinc ion conservation, the concentration of the zinc–ammonia complex was calculated to be 1.124 mol L^{-1}. Next, Milli-Q water (6.55 mL) was added to absolute ethanol (3 mL), followed by an aliquot (0.45 mL) of the 1.124 mol L^{-1} zinc–ammonia complex solution, resulting in a 0.05 mol L^{-1} zinc–ammonia ethanol solution (30%). DA was introduced to achieve a final concentration of 3 mg mL^{-1}. Then, an aliquot (5 mL) of the above solution was introduced into a six-well plate containing Aga template microspheres (0.5 mL per well), and the mixture was subjected to reciprocal shaking in a thermostatic water bath at 25 °C and 120 rpm for 1–12 h for coating to proceed. After the reaction, the microspheres were thoroughly washed several times with Milli-Q water to obtain Aga-PDA/Zn^{II}, which were dispersed in Milli-Q water or PBS for subsequent use. To investigate the effect of reaction time, the reaction time was varied (1, 2, 6, and 12 h), while keeping all other conditions constant.

Preparation of Aga-PDA. A 3 mg mL^{-1} DA solution in Tris-HCl (pH 8.5) served as the control for the preparation of Aga-PDA. An aliquot (5 mL) of the DA solution was introduced into a six-well plate containing Aga template (0.5 mL per well), and the mixture was subjected to reciprocal shaking in a thermostatic water bath at 25 °C and 120 rpm for 6 h. After the reaction, the resulting product was thoroughly washed several times with Milli-Q water to obtain purified Aga-PDA, which was dispersed in Milli-Q water or PBS for subsequent use.

Protein Adsorption Analysis. For the analysis, a known amount (1 mg) of Aga, Aga-PDA, or Aga-PDA/Zn^{II} was placed in 5 mL centrifuge tubes, followed by the addition of FITC-HSA solution (2 mL, 200 μg mL^{-1}). The tubes were sealed and incubated in a thermostatic shaker at 37 °C and 135 rpm for 24 h to allow for adsorption to proceed. After incubation, the supernatant was collected, and the residual absorbance of the solution was measured using a fluorescence spectrophotometer.

For construction of the calibration curve, a 200 μg mL^{-1} FITC-HSA stock solution was prepared and subsequently diluted stepwise to obtain solutions with concentrations of 100, 50, 25, 12.5, 6.25, 3.125, and 1.5625 μg mL^{-1}. The fluorescence intensity of each solution was then measured using a fluorescence spectrophotometer.

Preparation of Bioactive Superstructures. Bioactive superstructures were prepared through the incorporation of HSA to Aga-PDA/ Zn^{II}, yielding **H**SA-**A**ga-**P**DA/Zn^{II} (denoted as HAP/ Zn^{II}). In a typical experiment, 200 μg mL^{-1} HSA solution was prepared using Tris-HCl buffer (pH 8.0). Dried Aga-PDA/Zn^{II} superstructures (20 mg) were added to a 50 mL centrifuge tube and soaked in Tris-HCl buffer (pH 8.0) for 12 h. After removing the buffer solution, the HSA solution (40 mL, 200 μg mL^{-1}) was added to the tube, and incubated at 37 °C and 135 rpm for 24 h. After the incubation period, the supernatant was removed, and the superstructures were gently washed three times with Tris-HCl buffer to remove excess HSA, yielding bioactive superstructures. The obtained HAP/Zn^{II} superstructures were dispersed in Tris-HCl buffer (pH 8.0) and stored at 4 °C for further use.

Characterization. The morphologies of the dried Aga, Aga-PDA, Aga-PDA/Zn^{II}, and HAP/Zn^{II} were characterized by scanning electron microscopy (SEM; Phenom G2 Pro, Phenom World, Netherlands). Structural analysis and energy-dispersive X-ray spectroscopy mapping of the individual PDA/Zn^{II} microspheres were performed by transmission electron microscopy (Tecnai G2 F20 S-TWIN, FEI, USA). The mechanical strength of the Aga, Aga-PDA, Aga-PDA/Zn^{II}, and HAP/Zn^{II} was determined using a microparticle strength tester (MF-WSFI, Njlead, China). The porosity, total pore volume, and total pore area of these materials were analyzed on a fully automated mercury porosimeter (AutoPore V, Micromeritics, USA); the absolute error of the mercury intrusion porosimeter for measurements is ± 1%. Thermogravimetric analysis (TG209F1, NETZSCH, Germany) was performed to assess the thermal stability of Aga, Aga-PDA, and Aga-PDA/Zn^{II}. The zinc ion content and stability of these materials were characterized by inductively coupled plasma mass spectrometry (ICP-MS, 7900, Agilent, USA). Bilirubin adsorption and superstructure stability experiments were conducted on a UV–vis spectrophotometer (UV-2700, Shimadzu, Japan). The chemical structures of Aga, Aga-PDA, Aga-PDA/Zn^{II}, and HAP/Zn^{II} were further characterized using a tip-enhanced Raman spectrometer (inVia, Renishaw, UK). FITC-HSA adsorption on the superstructures was assessed using a fluorescence spectrophotometer (RF-5301PC, Shimadzu, Japan). The ζ-potentials of Aga, Aga-PDA, Aga-PDA/Zn^{II}, and HAP/Zn^{II} were measured after dispersing the materials in PBS (pH 7.4) via ultrasonication, using dynamic light scattering (Zetasizer Nano-ZS, Malvern Instruments, UK). The surface elemental composition of the dried Aga-PDA/Zn^{II} and HAP/Zn^{II} was analyzed by X-ray photoelectron spectroscopy (XSAM 800, Kratos, UK). The secondary structure of HSA before and after reaction with the Aga-PDA/Zn^{II} was characterized by circular dichroism spectroscopy (Chirascan Plus, Applied Photophysics, UK), and the data were analyzed using CDNN software.

Mechanistic Investigation and Stability Assay of Aga-PDA/Zn^{II}. A known amount (3 mg) of Aga-PDA/Zn^{II} was dispersed separately in 100 mM NaCl, urea, ethylenediaminetetraacetic acid (EDTA), or Tween 20 solution (3 mL). The mixtures were incubated on a shaker at 25 °C and 170 rpm for 6 h. Then, an aliquot (1 mL) of the supernatant was collected and filtered through a 0.22 μm membrane filter for UV–vis spectroscopy analysis. In addition, a further aliquot (1

mL) of the supernatant was diluted and digested with concentrated nitric acid for ICP measurements to determine the zinc ion content. The zinc ion loss (%) was calculated using the following equation:

$$\text{Zinc ion loss} = (m_0 - m_s)/m_0 \times 100\%$$

where m_0 is the zinc ion content in the original Aga-PDA/Zn^{II} and m_s is the amount of zinc ions released from Aga-PDA/ Zn^{II} after immersion in different solutions.

Extended X-Ray Absorption Fine Structure. Extended X-ray absorption fine structure (EXAFS) measurements were performed at the 5S1 X-ray absorption beamline of Aichi Synchrotron Radiation Center. This beamline adopts a double-bounce channel-cut Si (111) monochromator for mono-beam X-ray absorption spectroscopy. The end-station is equipped with three ionization chambers and a seven-element SDD detector after the sample position for transmission and fluorescence mode X-ray absorption spectroscopy. The photon flux on the sample ranges from 3×10^{10} to 4×10^{10} photons s^{-1} for X-ray energy from 5–9 keV in the low energy mode. In the normal energy mode, the photon flux on the sample ranges from 1×10^{11} to 2.2×10^{10} photons s^{-1} for X-ray energy from 7–18 keV. In the high energy mode, the photon flux on the sample ranges from 2.3×10^{10} to 5×10^{9} photons s^{-1} for X-ray energy from 17–22 keV. The EXAFS data were processed in Athena (version 0.9.26) for background, pre-edge line, and post-edge line calibrations. Fourier-transform fitting was carried out in Artemis (version 0.9.26). A k^2 weighting, a k-range of 3–12 $Å^{-1}$, and an R range of 1–3 Å were used for fitting of the Zn foil, whereas a k-range of 3–11 $Å^{-1}$, and an R range of 1–3 Å were used for fitting of the samples. The four parameters—coordination number (CN), bond length (R), Debye–Waller factor, and E_0 shift (ΔE_0)—were fitted without any parameters fixed, σ^2 was set. For Wavelet transform analysis, $\chi(k)$ exported from Athena was imported into the Hama Fortran code. The parameters are listed as follow: R range, 1–4 Å; k range, 0–12 $Å^{-1}$ for samples; k weight, 2; and Morlet function with $\kappa = 10$. $\sigma = 1$ was used as the mother wavelet to provide the overall distribution.

Molecular Docking. Molecular docking was performed between the PDA/Zn^{II} molecular structural unit and HSA.[1] The docking procedure was carried out using AutoDock Vina

(version 1.2.3),[2-3] which employs an iterated local search algorithm to perform repeated local searches and iterations to identify the optimal binding conformations. The conformation with the lowest binding energy was selected as the final docking result. Specifically, AutoDockTools (version 1.5.7) was first used to preprocess the ligand and protein structures, define appropriate docking sites, and generate configuration files for AutoDock Vina docking. Subsequently, AutoDock Vina conducted iterative local searches to obtain the most stable docking conformation. After docking, PyMOL (version 2.3) was used for 3D visualization and analysis of the resulting complexes,[4] while LigPlot+ was employed to identify and illustrate 2D interaction patterns.[5] PyMOL identifies and visualizes hydrogen bonds and conformations in 3D space based on geometric algorithms, whereas LigPlot+ generates 2D interaction diagrams using built-in geometric and statistical rules. Together, these tools provide complementary perspectives for analyzing molecular interactions

Bilirubin Adsorption. To prepare a homogeneous bilirubin solution, an appropriate amount of bilirubin was first dissolved in a small volume of dimethyl sulfoxide and 0.1 M Na_2CO_3 solution, and then diluted with pH 7.4 PBS buffer to the desired concentration. A known amount (40 mg) of Aga, Aga-PDA, Aga-PDA/Zn^{II}, or HAP/Zn^{II} was added to bilirubin solution (50 mL, 200 μg mL^{-1}). The mixtures were incubated in a thermostatic shaker at 37 °C and 135 rpm under dark conditions. At predetermined time intervals, supernatant samples were collected and filtered through a 0.45 μm filter. The absorbance of the filtrate was measured at 438 nm via UV–vis spectrophotometry until adsorption equilibrium was reached. The concentration of bilirubin was calculated using a standard calibration curve. Each group was tested in triplicate. A standard calibration curve was established as follows: a 200 μg mL^{-1} bilirubin stock solution was prepared and serially diluted to concentrations of 100, 50, 25, 12.5, 6.25, and 3.125 μg mL^{-1}. The absorbance of each solution was then measured at 438 nm via UV–vis spectrophotometry.

The bilirubin adsorption capacity, q (mg g^{-1}), and removal efficiency, R (%), of the tested material/adsorbent were calculated, as follows:

$$q = \frac{(C_0 - C_t)V}{W}$$

$$R = \frac{(C_0 - C_t)}{C_0} \times 100\%$$

where C_0 and C_t (mg L^{-1}) are the initial and equilibrium bilirubin concentrations in the solution, V (L) is the solution volume, and W (g) is the mass of the adsorbent.

Effect of Albumin on Bilirubin Adsorption by HAP/Zn^{II}. A known amount (8 mg) of HAP/Zn^{II} was added to a bilirubin solution (10 mL, 200 μg mL^{-1}) containing BSA at various concentrations (0–50 g L^{-1}). After the adsorption equilibrium was reached, the solution was filtered through a 0.45 μm syringe filter. The residual bilirubin concentration was determined by UV–vis spectrophotometry, and the adsorption capacity and removal efficiency were calculated.

Simulated Plasma Adsorption. A simulated hyperbilirubinemia plasma solution was prepared by dissolving 200 mg L^{-1} bilirubin, 50 g L^{-1} BSA, and 9 g L^{-1} NaCl in PBS buffer (pH 7.4). A know amount (40 mg) of HAP/Zn^{II} was added to an aliquot (50 mL) of the simulated plasma solution and incubated in a thermostatic shaker at 37 °C and 135 rpm under dark conditions. At predetermined time intervals, samples of the supernatant were taken, filtered through a 0.45 μm filter, and analyzed by UV–vis spectrophotometry to determine the concentration of bilirubin. The adsorption capacity of bilirubin at different time points was then calculated.

Computational Analysis. To understand the intrinsic driving forces underlying bilirubin removal by Aga, Aga-PDA, Aga-PDA/Zn^{II}, or HAP/Zn^{II}, quantum chemical calculations were performed to determine the interaction energies between bilirubin and the repeating units of Aga, PDA, and PDA/Zn^{II}. All calculations were carried out using density functional theory using the Gaussian 16 software package. The structures of the studied molecules were fully optimized at the PBE0-D3BJ/def2-SVP level of theory. The vibrational frequencies of the optimized structures were carried out at the same level. The structures were characterized as a local energy minimum on the potential energy surface by verifying that all the vibrational frequencies were real. Geometry optimization of the complexes was performed with the semi-empirical xtb program. The GFN2 parameter set was chosen for all the calculations. The

interaction energy (ΔE) of the optimized complexes was calculated at the PBE0-D3BJ/def2-SVP level of theory. ΔE is defined as the difference between the energy of the complex (i.e., E(AB)) and the sum of energies of the monomers (i.e., E(A) + E(B)), as follows: $\Delta E = E(AB) - (E(A) + E(B))$. The Independent Gradient Model based on Hirshfeld partition of molecular density (IGMH) analysis,[6] electron density difference mapping of the complex and monomers, analysis of the total density of states and partial density of states of the complexes were performed using the Multiwfn software.[7] The Visual Molecular Dynamics program was used to plot the color-filled iso-surface graphs to visualize the molecular electrostatic potential, the electron density difference maps, and the IGMH isosurfaces.[8]

Antibacterial Activity: Bacterial Growth Curves. To evaluate bacterial growth, a known amount (5 mg) of Aga, Aga-PDA, Aga-PDA/Zn^{II}, or HAP/Zn^{II} was introduced into 48-well plates. Then, an aliquot (500 μL) of a bacterial suspension of *E. coli* or *S. aureus* diluted to 1.0×10^3 CFU mL^{-1} was added to each well. The plates were incubated at 37 °C in a temperature-controlled shaker with agitation at 150 rpm. At specific time intervals (0, 1, 2, 6, 12, and 24 h), the optical density (OD) at 600 nm was measured using a microplate reader (Varioskan LUX, Thermo Fisher Scientific). The same procedure was adopted for the control group but with no added samples.

Antibacterial Activity: Relative Bactericidal Rate. A known amount (5 mg) of Aga, Aga-PDA, Aga-PDA/Zn^{II}, or HAP/Zn^{II} was introduced into 48-well plates. Then, an aliquot (500 μL) of a bacterial suspension (*E. coli* or *S. aureus*, 1.0×10^3 CFU mL^{-1}) was added to each well. The plates were incubated overnight at 37 °C with agitation at 150 rpm in a shaking incubator. After incubation, an aliquot (100 μL) of the bacterial suspension from each well was spread on agar plates, and each sample was tested in triplicate. The agar plates were inverted and incubated at 37 °C for 24 h. Bacterial mortality (%) was calculated based on colony counts using the following equation:

$$\text{Relative bactericidal rate} = (n_c - n_s)/n_c \times 100\%$$

where n_c is the number of colonies in the control group and n_s is the number of colonies in the

experimental group. The control group had no added samples.

Live/Dead Staining and Morphological Characterization. Live/dead staining was performed to assess bacterial viability after contact with the tested materials. Circular glass coverslips were placed in 48-well plates, and an aliquot (500 μL) of a bacterial suspension (1.0×10^4 CFU mL^{-1}) was added to each well. The plates were incubated overnight at 37 °C on a shaker. Subsequently, a known amount (5 mg) of Aga, Aga-PDA, Aga-PDA/Zn^{II}, or HAP/Zn^{II} was added (control wells were untreated), followed by further incubation for 12 h at 37 °C. After incubation, the samples were gently rinsed with PBS and stained in the dark for 20 min with the LIVE/DEAD staining solution (150 μL, prepared by mixing 1.5 μL SYTO 9 and 1.5 μL propidium iodide (PI) in 1 mL PBS). SYTO 9 stains both live and dead cells, producing green fluorescence, while PI selectively penetrates membrane-compromised (dead) cells and, upon binding nucleic acids, overrides the SYTO 9 signal. The staining solution was then removed, and the coverslips were observed via confocal laser scanning microscopy (STELLARIS 5, Leica). Images were collected and analyzed using ImageJ software to quantify the fluorescence intensities of live (green emission) and dead (red emission) bacteria. The bacterial survival and death rates (%) were calculated as follows:

$$\text{Bacterial survival rate} = I_{\text{live}}/(I_{\text{live}} + I_{\text{dead}}) \times 100\%$$

$$\text{Bacterial death rate} = I_{\text{dead}}/(\text{I}_{\text{live}} + I_{\text{dead}}) \times 100\%$$

where I_{live} and I_{dead} represent the fluorescence intensities of live and dead bacteria, respectively.

The morphologies of *E. coli* and *S. aureus* treated with Aga, Aga-PDA, Aga-PDA/Zn^{II}, or HAP/Zn^{II} were further examined using the coverslip method. Glass coverslips were placed in 48-well plates, and a bacterial suspension (500 μL, 1.0×10^4 CFU mL^{-1}) was added to each well and incubated overnight at 37 °C. Then, a known amount (5 mg) of Aga, Aga-PDA, Aga-PDA/Zn^{II}, or HAP/Zn^{II} was added (the control group was untreated), and the cultures were further incubated for 12 h at 37 °C on a shaker. After incubation, the samples were fixed overnight in 2.5% glutaraldehyde solution (500 μL), followed by dehydration in 25%, 50%, 75%, and 100% ethanol/water solutions for 10 min each. The dried samples were examined

using SEM. For transmission electron microscopy analysis (JEM-1400FLASH, JEOL, Japan), portions of glutaraldehyde-fixed samples from the control, Aga-PDA/Zn^{II}, and HAP/Zn^{II} groups were further post-fixed with 1% osmium tetroxide. The samples were then dehydrated sequentially with 30%, 50%, 70%, 80%, 90%, 95%, and 100% (three times) acetone solutions. The dehydrated specimens were embedded in Epon-812 resin, sectioned into ultrathin slices (60–90 nm) using an ultramicrotome (UC7rt), and mounted on copper grids. The sections were stained with uranyl acetate for 10–15 min and lead citrate for 1–2 min.

Zinc Ion Release Assay. Aga-PDA/Zn^{II} (3 mg) or HAP/Zn^{II} (3 mg) was dispersed in Milli-Q water (10 mL) and incubated at 37 °C with shaking at 150 rpm. At predetermined time points, the supernatant was collected for ICP-MS analysis to determine the zinc ion concentration. In addition, the surface morphology of HAP/Zn^{II} after 1 day of incubation was characterized by SEM.

Antibacterial Activity under Blood-Containing Conditions. A known amount (5 mg) of Aga-PDA/Zn^{II} or HAP/Zn^{II} was placed into individual wells of a 48-well plate and pre-incubated with PBS for at least 1 h. Subsequently, an aliquot (500 μL) of a 50% whole blood or 50% plasma suspension containing *S. aureus* (1.0×10^3 CFU mL^{-1}) was added. The 50% whole blood or 50% plasma suspension was prepared by mixing equal volumes of whole blood or plasma with PBS (1:1, v/v). The plate was incubated at 37 °C with shaking at 150 rpm for 5 h. After incubation, the supernatants were diluted 10 fold, and an aliquot (100 μL) of the diluted bacteria-containing whole blood or plasma suspension was spread onto agar plates (three replicates per group). The agar plates were incubated upside down at 37 °C for 24 h, after which the bacterial killing rate was calculated based on the number of colony counts.

Hemolysis Rate Analysis. Physiological saline (10 mL) was introduced into centrifuge tubes, followed by Aga, Aga-PDA, Aga-PDA/Zn^{II}, or HAP/Zn^{II} (5 mg). The group with only physiological saline (10 mL) served as the negative control, while the group with deionized water (10 mL) served as the positive control. Each group was prepared in triplicate. All tubes were incubated in a 37 °C water bath for 30 min. Separately, citrated rabbit whole blood (New

Zealand White rabbit) (4 mL) was diluted with physiological saline (5 mL). Then, an aliquot (0.2 mL) of the diluted blood was added to each pre-incubated tube containing Aga, Aga-PDA, Aga-PDA/Zn^{II}, or HAP/Zn^{II}, mixed gently, and incubated at 37 °C for an additional 1 h. After incubation, the suspension was transferred to centrifuge tubes and centrifuged at 3000 rpm for 5 min. Subsequently, the supernatant (150 μL) was carefully collected and transferred to a 96-well plate. The OD was measured at 540 nm using a microplate reader. The hemolysis rate was calculated, as follows:

$$\text{Hemolysis rate} = (OD_t - OD_{nc})/(OD_{pc} - OD_{nc}) \times 100\%$$

where OD_t is the OD of the test sample, OD_{nc} is the OD of the negative control, and OD_{pc} is the OD of the positive control.

Cytotoxicity. The cytotoxicity of Aga, Aga-PDA, Aga-PDA/Zn^{II}, and HAP/Zn^{II} was evaluated by measuring the viability of L929 mouse fibroblast cells. Briefly, an aliquot (100 μL) of complete culture medium containing 5000 cells was added to each well of a 96-well plate. Aga, Aga-PDA, Aga-PDA/Zn^{II}, or HAP/Zn^{II} was then added at concentrations of 250 and 500 μg mL^{-1}, whereas wells without sample treatment served as the control group. The cells were incubated at 37 °C under 5% CO_2 for 24, 48, and 72 h. After incubation, the culture medium was removed and replaced with fresh medium (90 μL) and Cell Counting-Kit-8 reagent (10 μL), followed by further incubation for 2 h. The OD at 450 nm was measured using a microplate reader (Multiskan FC, Thermo Fisher Scientific). Cell viability (%) was calculated using the following formula:

$$\text{Cell viability} = (OD_{sample} - OD_{blank})/(OD_{control} - OD_{blank}) \times 100\%$$

where OD_{sample}, $OD_{control}$, and OD_{blank} represent the OD of the sample group, control group, and blank group, respectively. The blank group contained only complete culture medium. To ensure accuracy, each test condition was set up in five replicates, and the OD values were averaged.

Complete Blood Count. A known amount (5 mg) of Aga, Aga-PDA, Aga-PDA/Zn^{II}, or HAP/Zn^{II} was pre-soaked in PBS buffer for at least 2 h. Fresh rabbit whole blood was treated with 4 U mL^{-1} heparin sodium and 10 mmol L^{-1} $CaCl_2$, and gently mixed to ensure uniform

distribution. Then, the prepared whole blood (1 mL) was added to each PBS-pre-soaked sample. A glass bead group was used as the positive control, and a group with only rabbit whole blood was used as the blank control. Each group was prepared in triplicate. The tubes were gently inverted to mix the samples thoroughly with the blood and then incubated in a 37 °C water bath at 60 rpm for 1 h. After incubation, 5 mmol L^{-1} EDTA was added to stop the reaction, and the samples were analyzed using a blood cell analyzer (BC-2800Vet, Mindray, China).

Platelet Adhesion Assay. Aga, Aga-PDA, Aga-PDA/Zn^{II}, or HAP/Zn^{II} (1 mg) was immersed in isotonic PBS (pH 7.4) for at least 2 h. Platelet-rich plasma was obtained from rabbit whole blood anticoagulated with sodium citrate by centrifugation at 200*g* for 10 min. The microspheres were then incubated in platelet-rich plasma (300 μL) at 37 ± 1 °C for 90 min. After incubation, the samples were removed from the platelet-rich plasma and washed three times with PBS. The washed samples were fixed with 2.5% glutaraldehyde at room temperature (25 ± 2 °C) for 4 h. Subsequently, dehydration was carried out using graded ethanol solutions (25, 50, 75, and 100 wt%, 10 min each). The dehydrated samples were then dried for further analysis.

Whole Blood Clotting Time. A known amount (5 mg) of Aga, Aga-PDA, Aga-PDA/Zn^{II}, or HAP/Zn^{II} was incubated in PBS buffer for at least 2 h. The samples were then transferred into 3 mL centrifuge tubes. Subsequently, citrated rabbit whole blood (500 μL) was added to each tube and gently mixed with the microspheres. The tubes were incubated in a 37 °C water bath for 10 min. After incubation, of 0.1 M $CaCl_2$ (50 μL) solution was added to each tube and mixed gently. The tubes were inverted every 15 s, and the clotting time was carefully recorded. Each group was prepared in triplicate. A group without any microspheres was used as the blank control.

Anticoagulant Activity Studies. To evaluate the in vitro anticoagulant activity of the microspheres, fresh venous blood was centrifuged at 3000 rpm for 15 min to obtain platelet-poor plasma. Activated partial thromboplastin time, prothrombin time, and thrombin time were measured according to the instructions provided with the assay kits to analyze the anticoagulant

mechanism. All tests were performed using a semi-automated coagulation analyzer (PUN-2048A, Prolong, China).

ELISA Kit Assay. Bradykinin, kallikrein, von Willebrand factor, complement components 3a and 5a, interleukin-6, and interleukin-8 levels were measured using ELISA kits (Jiangsu Enzyme Rabbit Industrial Co., Ltd.) after incubation of Aga, Aga-PDA, Aga-PDA/Zn^{II}, or HAP/Zn^{II} with plasma. The experimental procedures were carried out according to the instructions provided in the kit manuals.

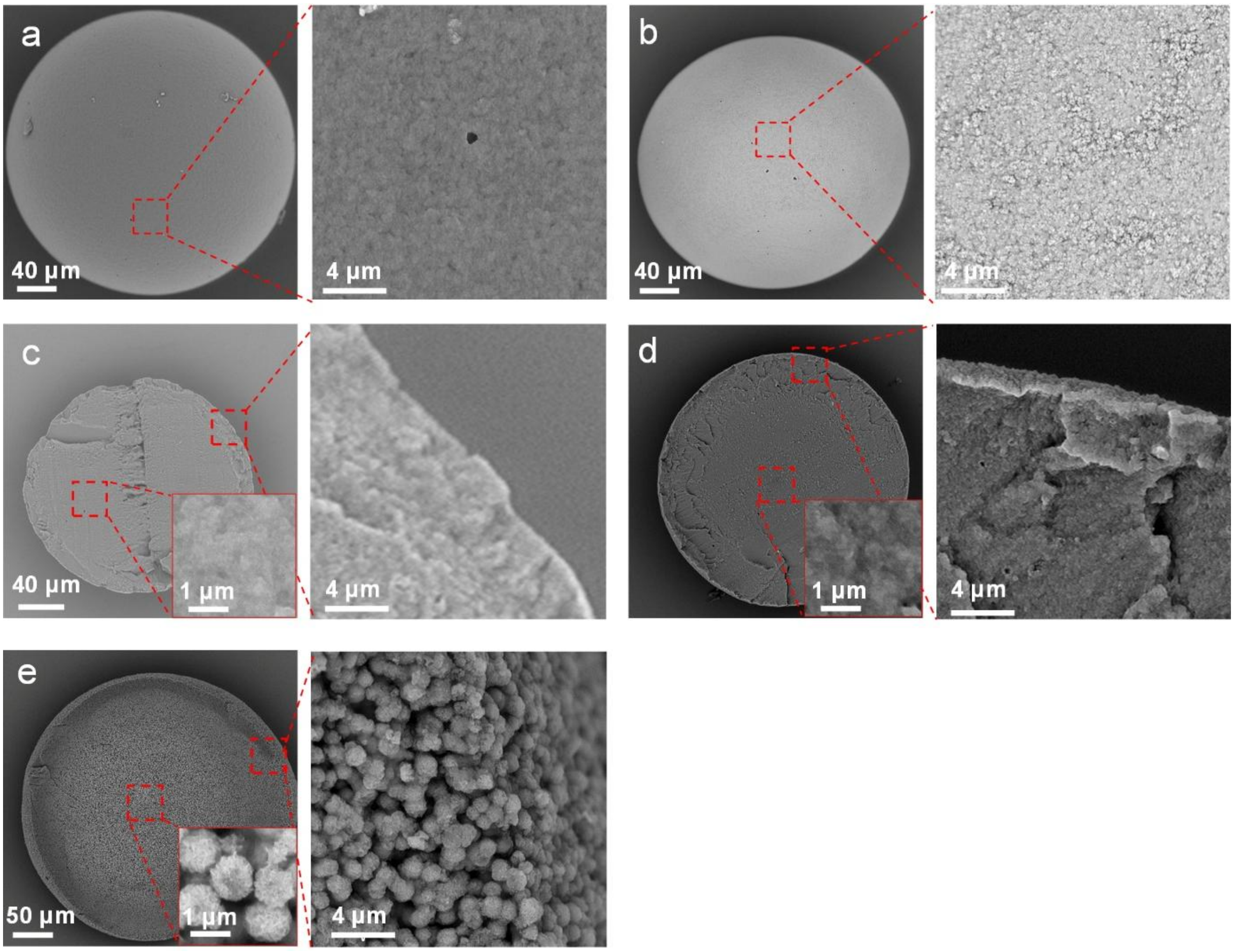

Figure S1. SEM images showing the (a,b) surface morphology and (c,d) cross-sectional morphology of Aga (a,c) and Aga-PDA (b,d). (e) SEM images of the cross-sectional morphology of Aga-PDA/Zn^{II}.

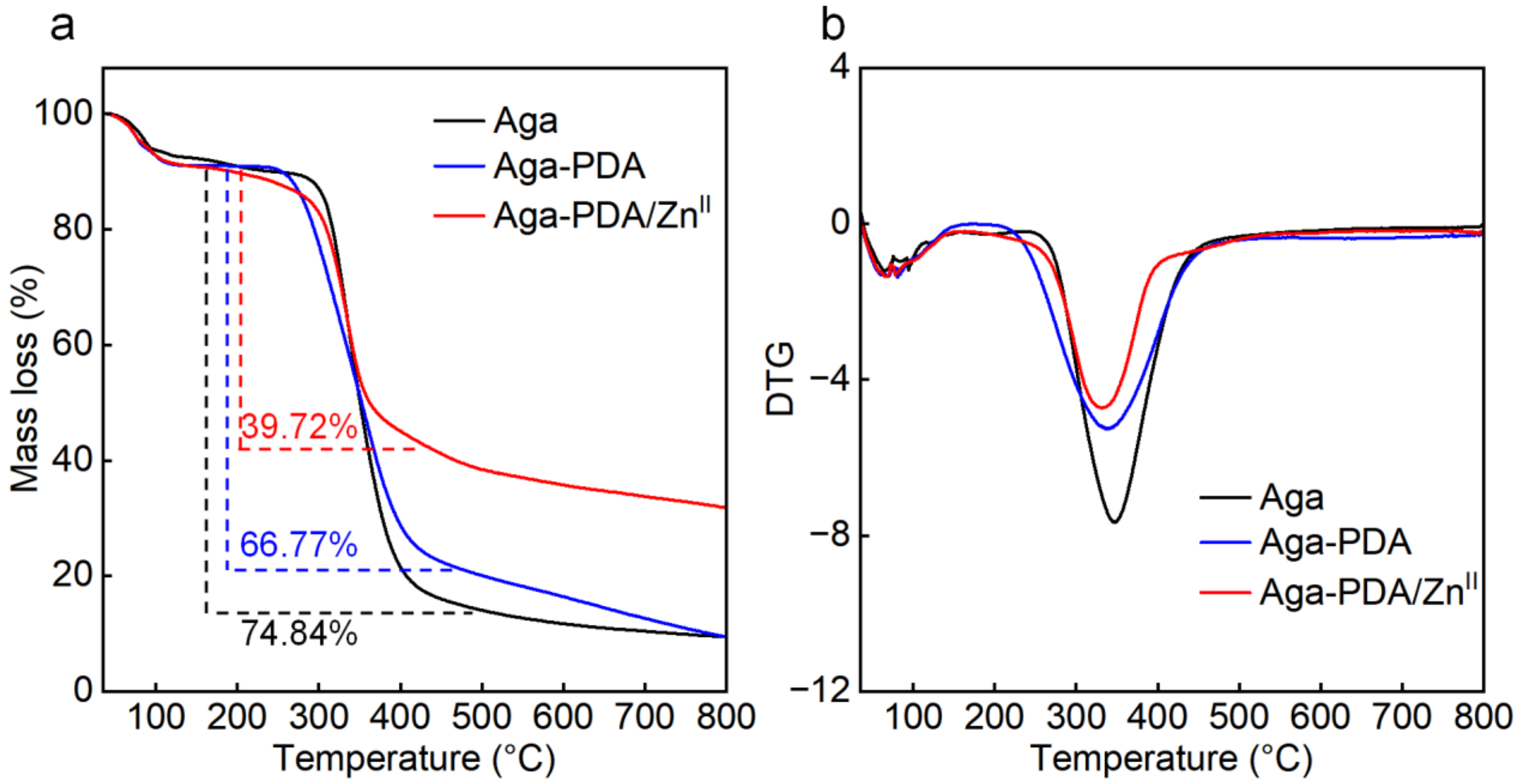

Figure S2. (a) Thermogravimetric curves and (b) differential thermogravimetric (DTG) curves of Aga, Aga-PDA, and Aga-PDA/Zn^{II}.

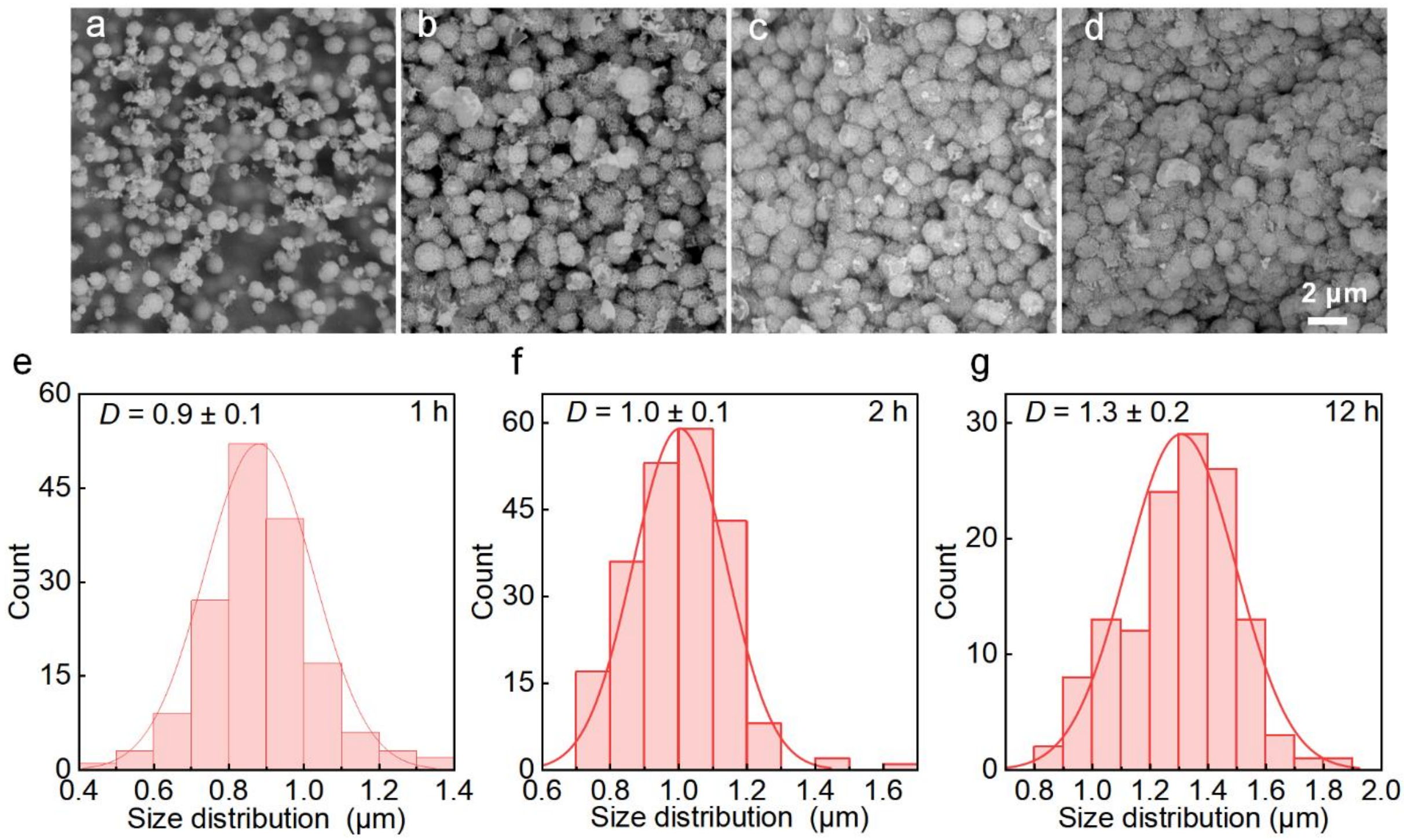

Figure S3. (a–d) SEM images showing the growth of PDA/Zn^{II} microspheres on the surface of the Aga-PDA/Zn^{II} superstructures prepared using reaction times of 1, 2, 6, and 12 h, respectively. (e–g) Size distributions of PDA/Zn^{II} microspheres on the Aga-PDA/Zn^{II} superstructures prepared using reaction times of 1 h (e), 2 h (f), and 12 h (g).

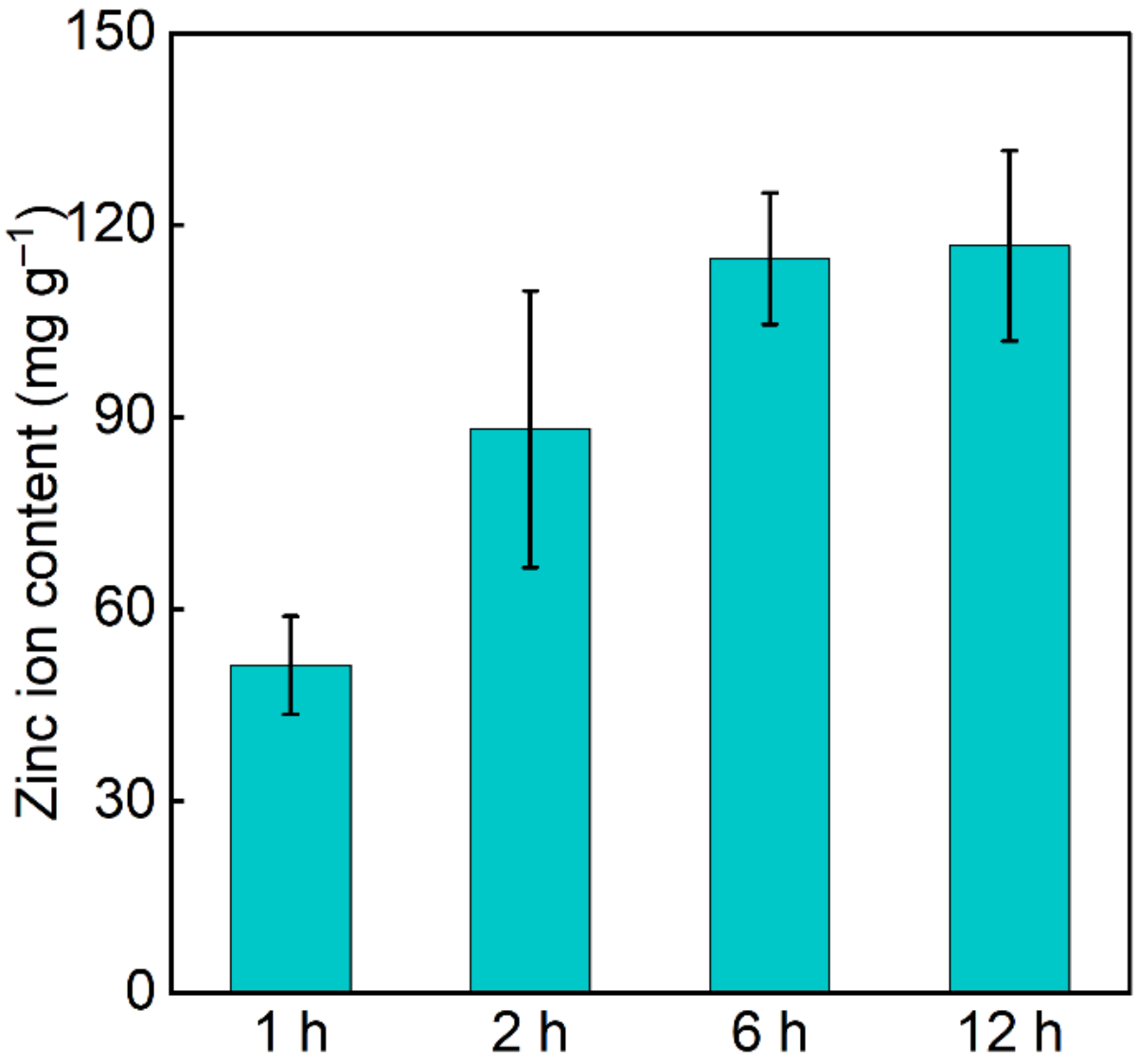

Figure S4. Zinc ion content of Aga-PDA/Zn^{II} prepared using different reaction times. Data are presented as mean ± standard deviation ($n \geq 3$).

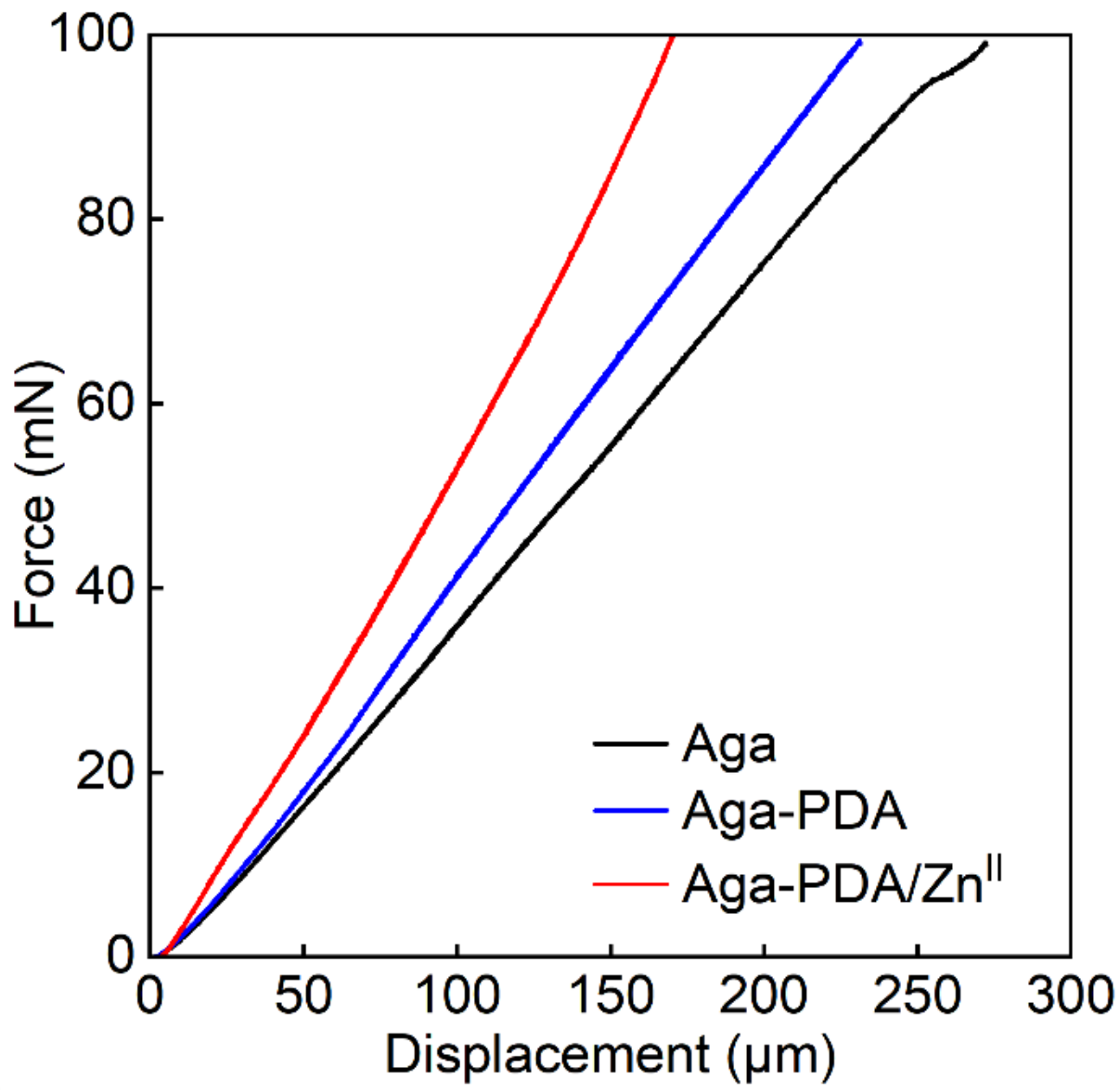

Figure S5. Force–displacement curves of Aga, Aga-PDA, and Aga-PDA/Zn^{II} measured using a microparticle strength tester.

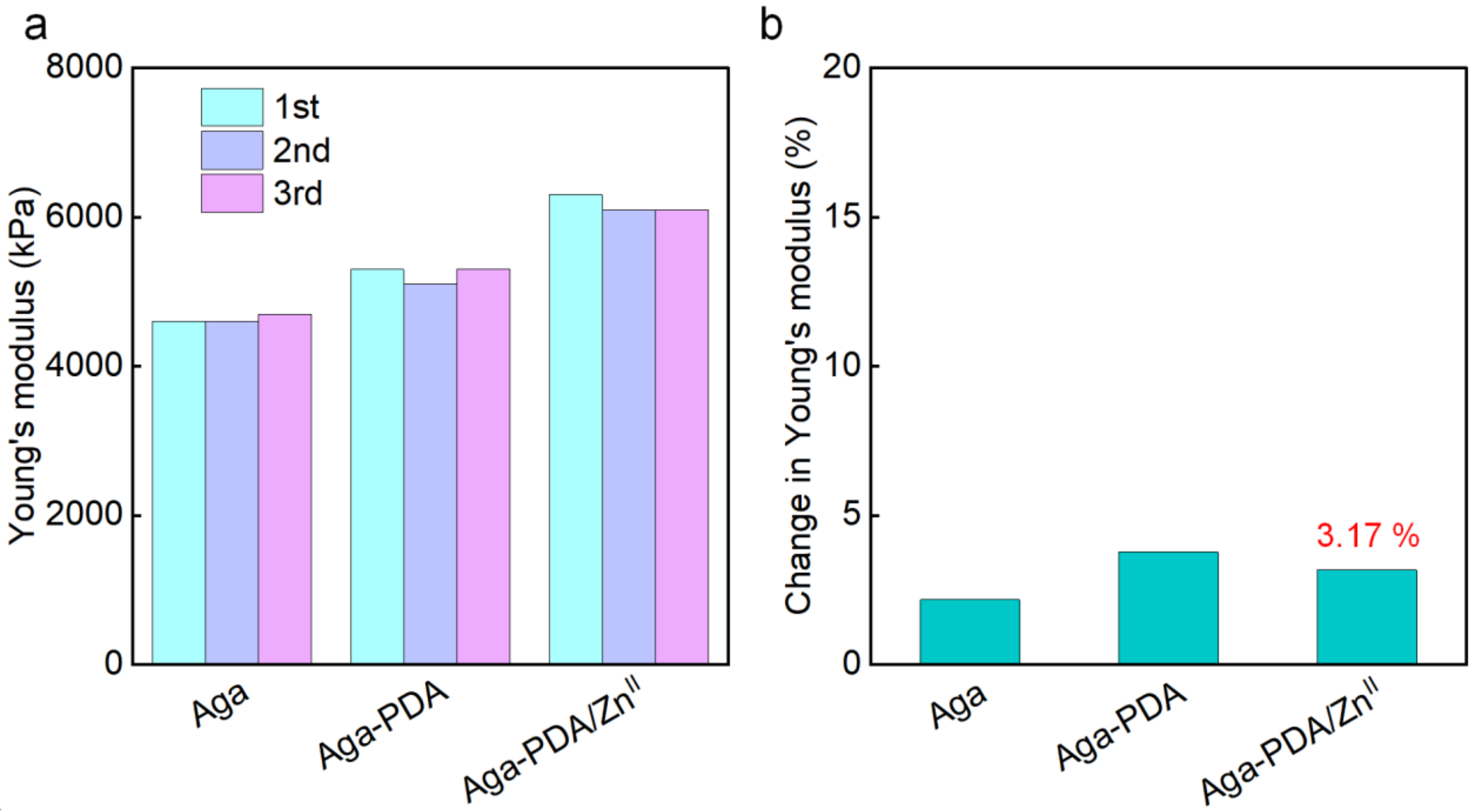

Figure S6. (a) Young's modulus values of Aga, Aga-PDA, and Aga-PDA/Zn^{II} after three repeated compression cycles. (b) Variations in the Young's modulus of Aga, Aga-PDA, and Aga-PDA/Zn^{II} after three repeated compressions.

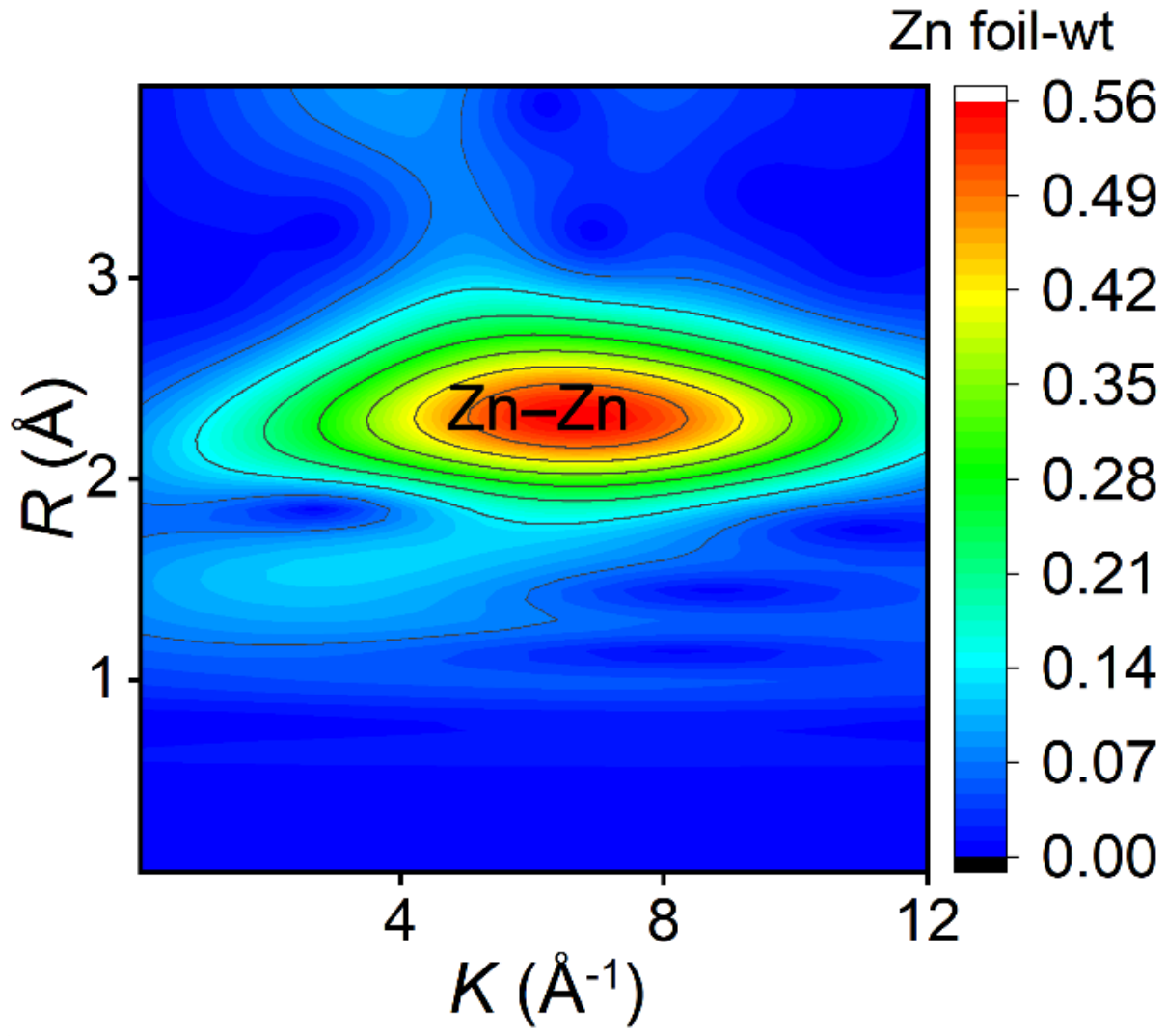

Figure S7. Wavelet transform analysis of Zn foil.

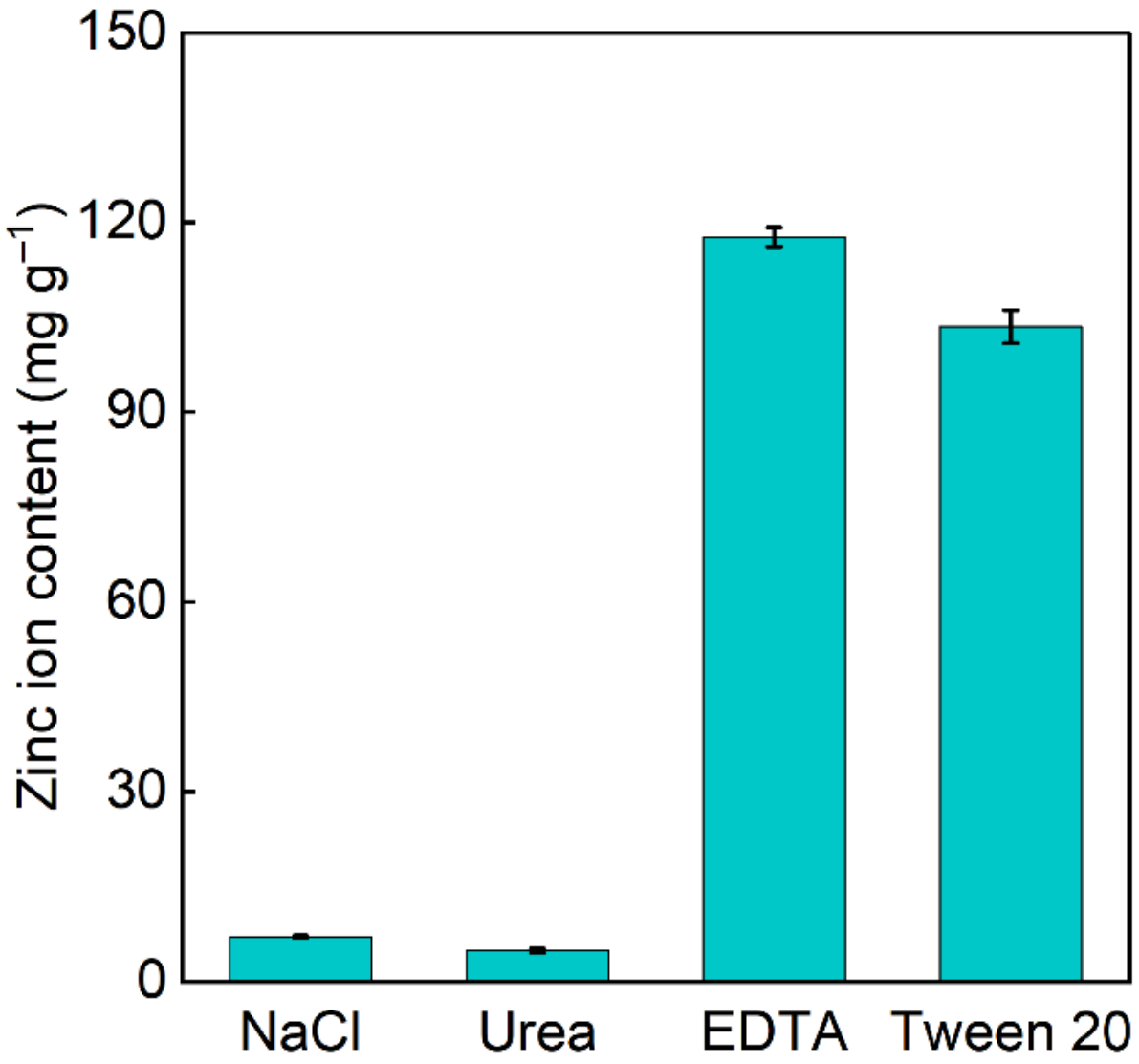

Figure S8. Zinc ion loss of Aga-PDA/Zn^{II} after immersion in NaCl, urea, EDTA, or Tween 20 solution. Data are presented as mean ± standard deviation ($n \geq 3$).

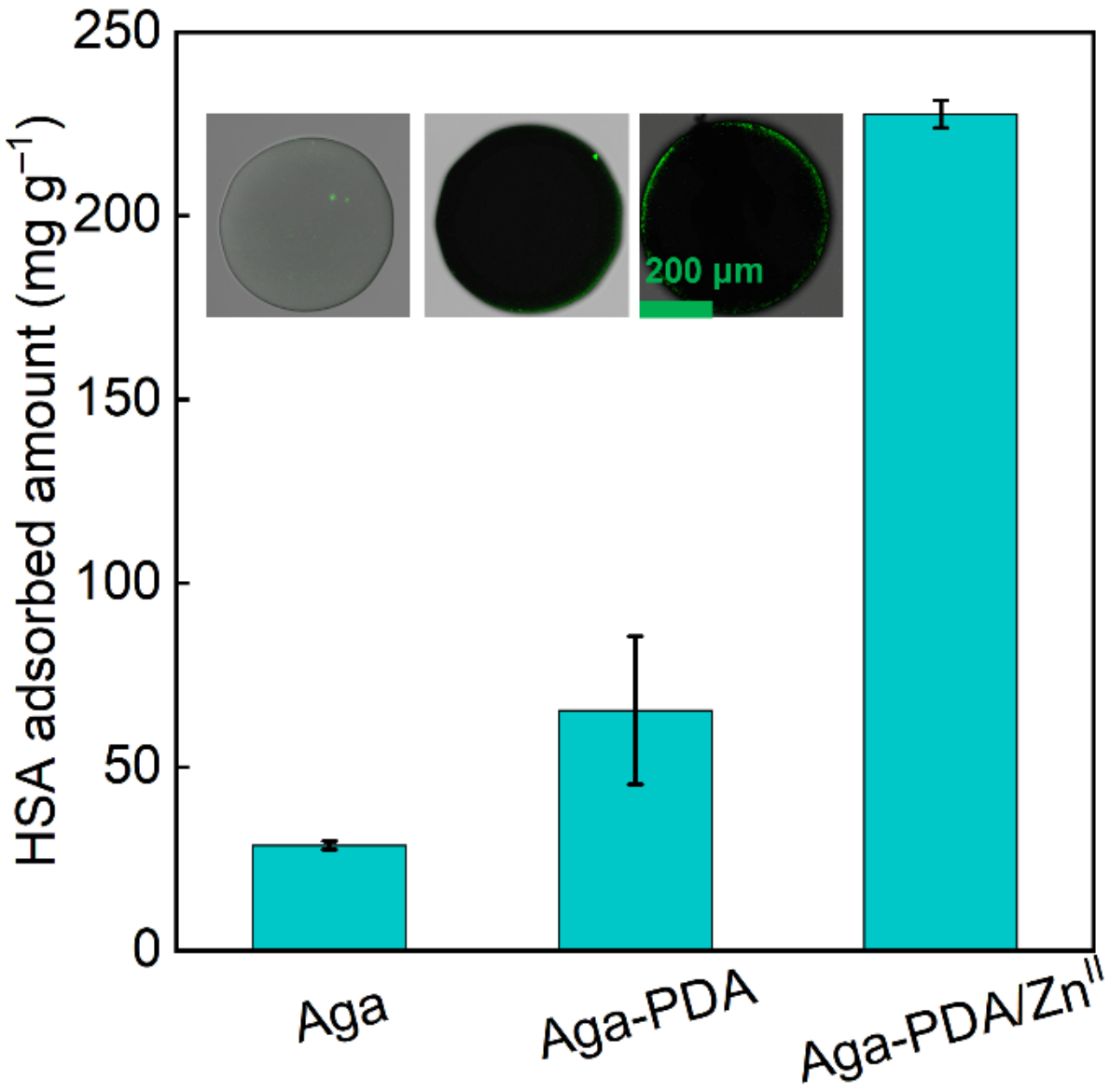

Figure S9. HSA loading amounts on the surfaces of Aga, Aga-PDA, and Aga-PDA/Zn^{II} after co-incubation with HSA. The insets show fluorescence images of Aga, Aga-PDA, and Aga-PDA/Zn^{II} (from left to right), where almost no fluorescence is observed on Aga, weak fluorescence is seen on Aga-PDA, and strong fluorescence is present on Aga-PDA/Zn^{II}. Data are presented as mean ± standard deviation ($n \geq 3$).

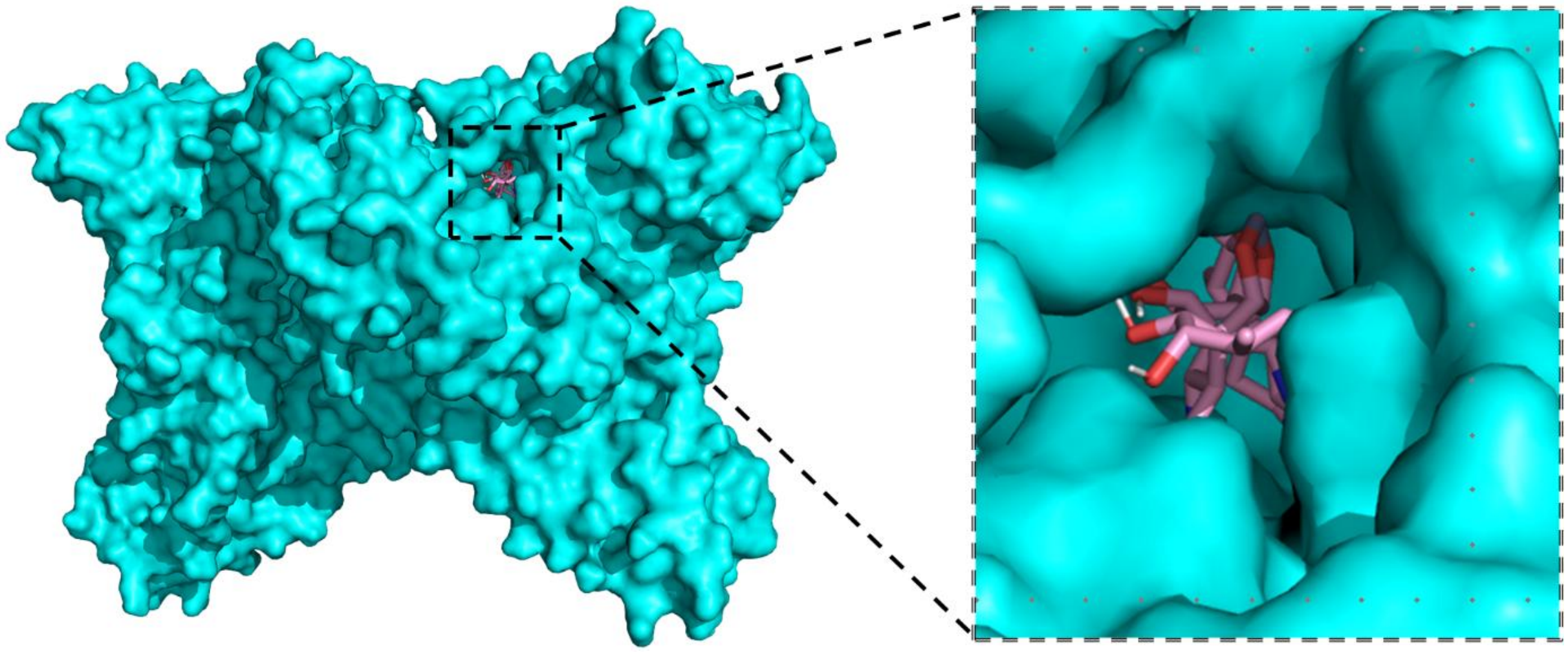

Figure S10. Schematic diagram of the surface structure of the PDA/Zn^{II}–HSA docking complex (binding pocket analysis).

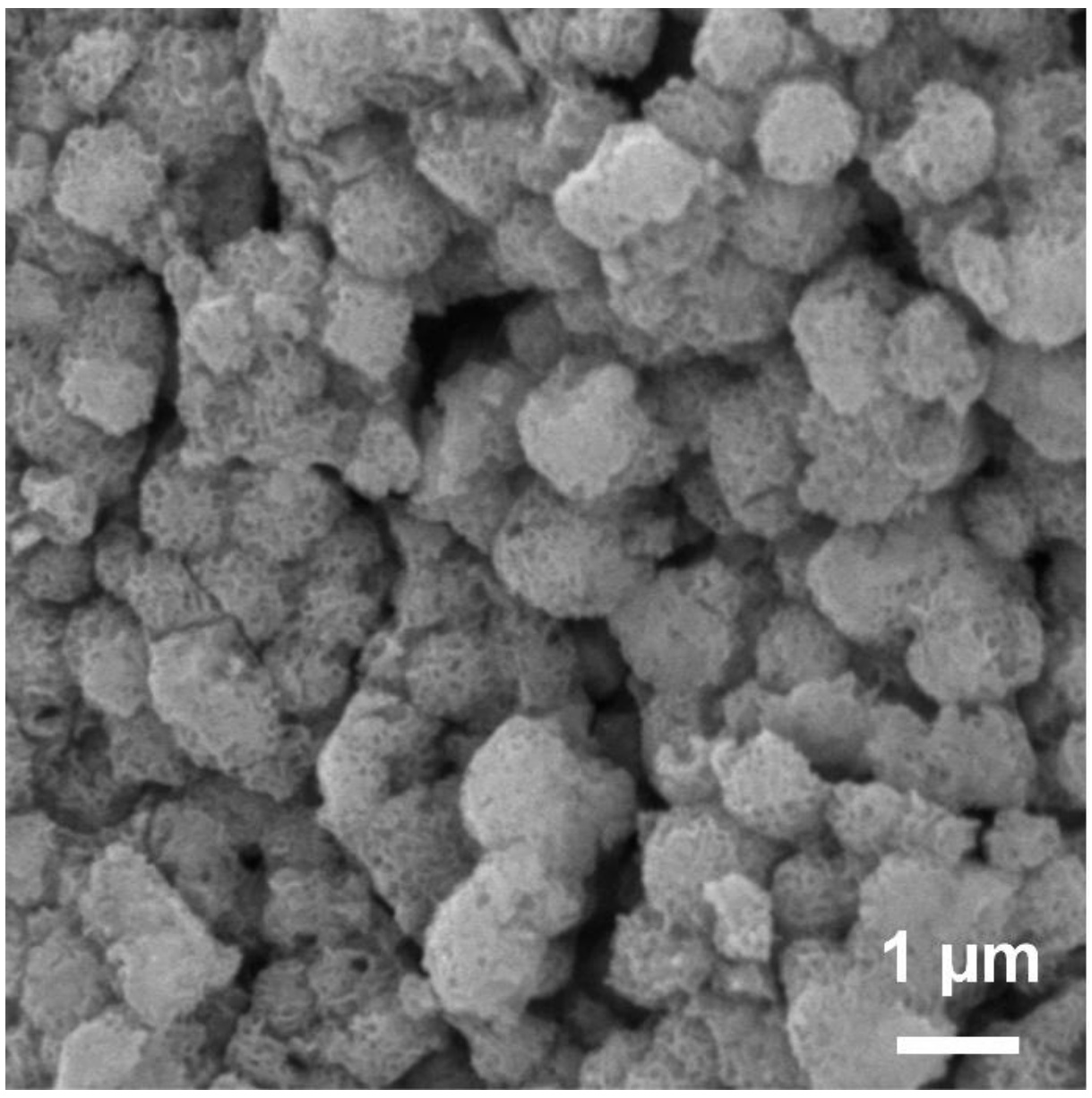

Figure S11. SEM image of HAP/Zn^{II} after incubation in deionized water for 1 day at 37 °C under agitation at 150 rpm.

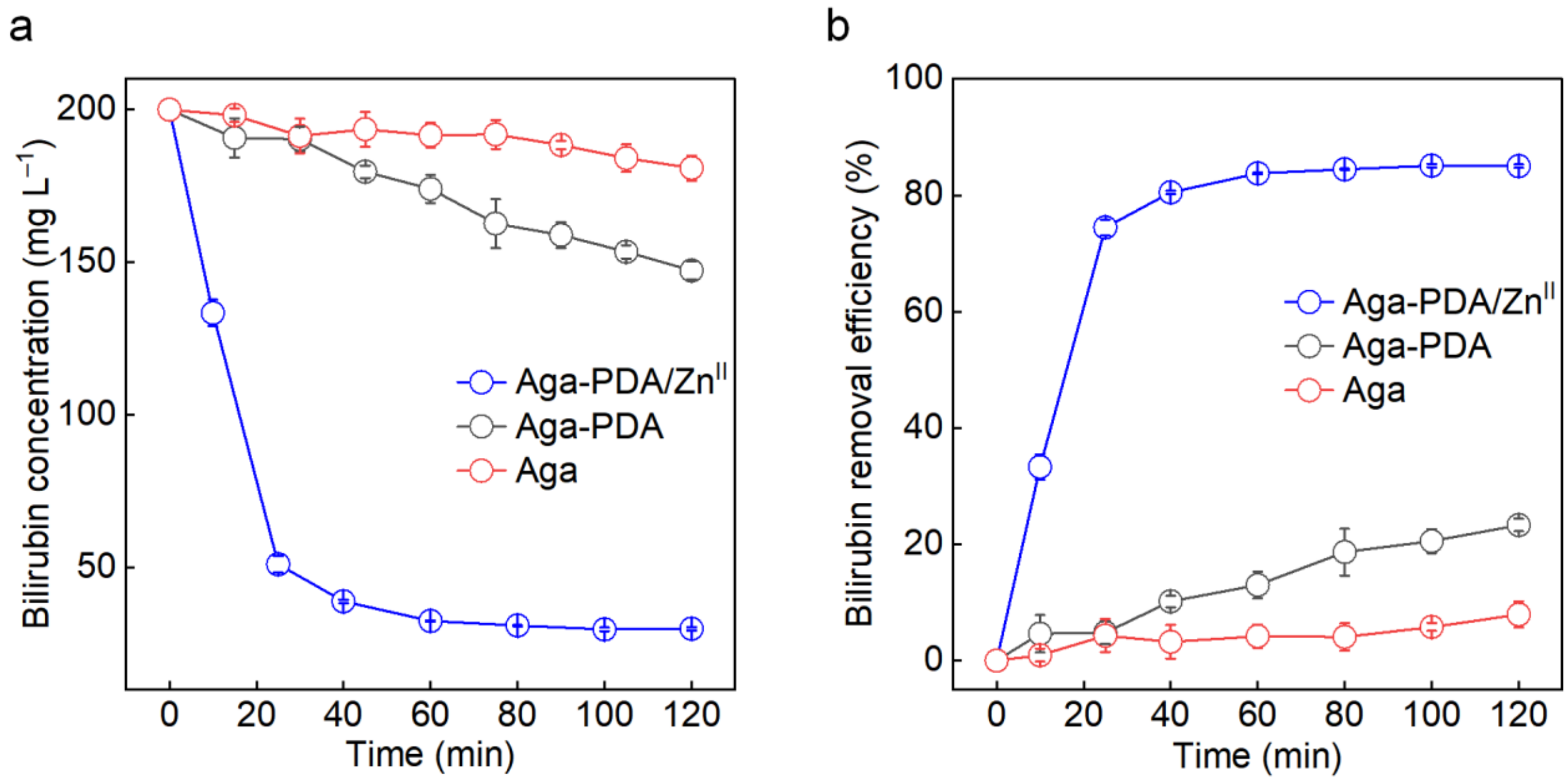

Figure S12. (a) Residual bilirubin concentration and (b) removal efficiency of bilirubin by Aga, Aga-PDA, or Aga-PDA/Zn^{II} as a function of contact time. Data are presented as mean ± standard deviation ($n \geq 3$).

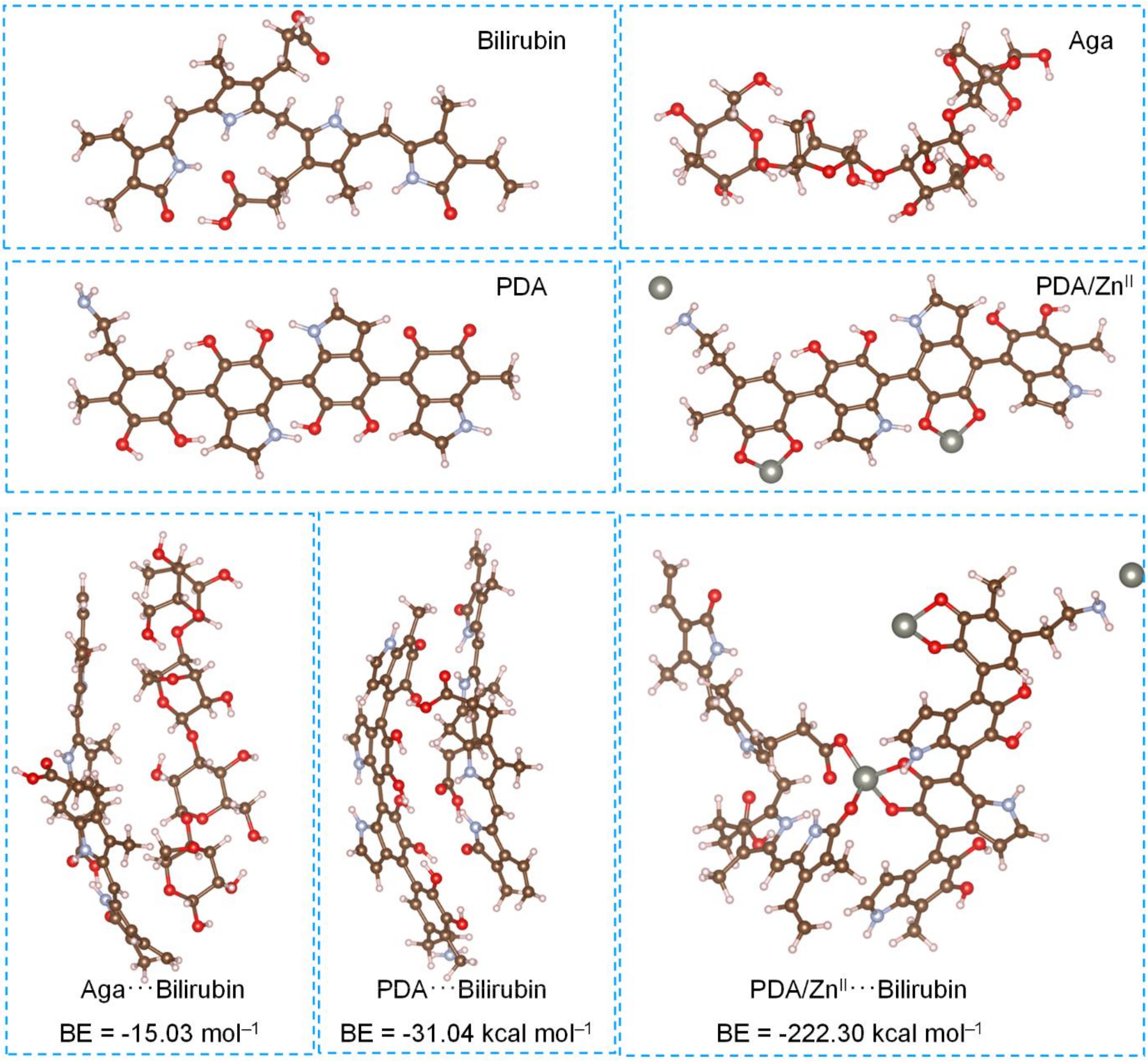

Figure S13. Optimized molecular structures of bilirubin, Aga, PDA, and PDA/Zn^{II}, as well as their adsorption complexes. The adsorption complexes of Aga–bilirubin, PDA–bilirubin, and PDA/Zn^{II}–bilirubin were optimized using density functional theory, and their corresponding binding energies (BEs) were calculated.

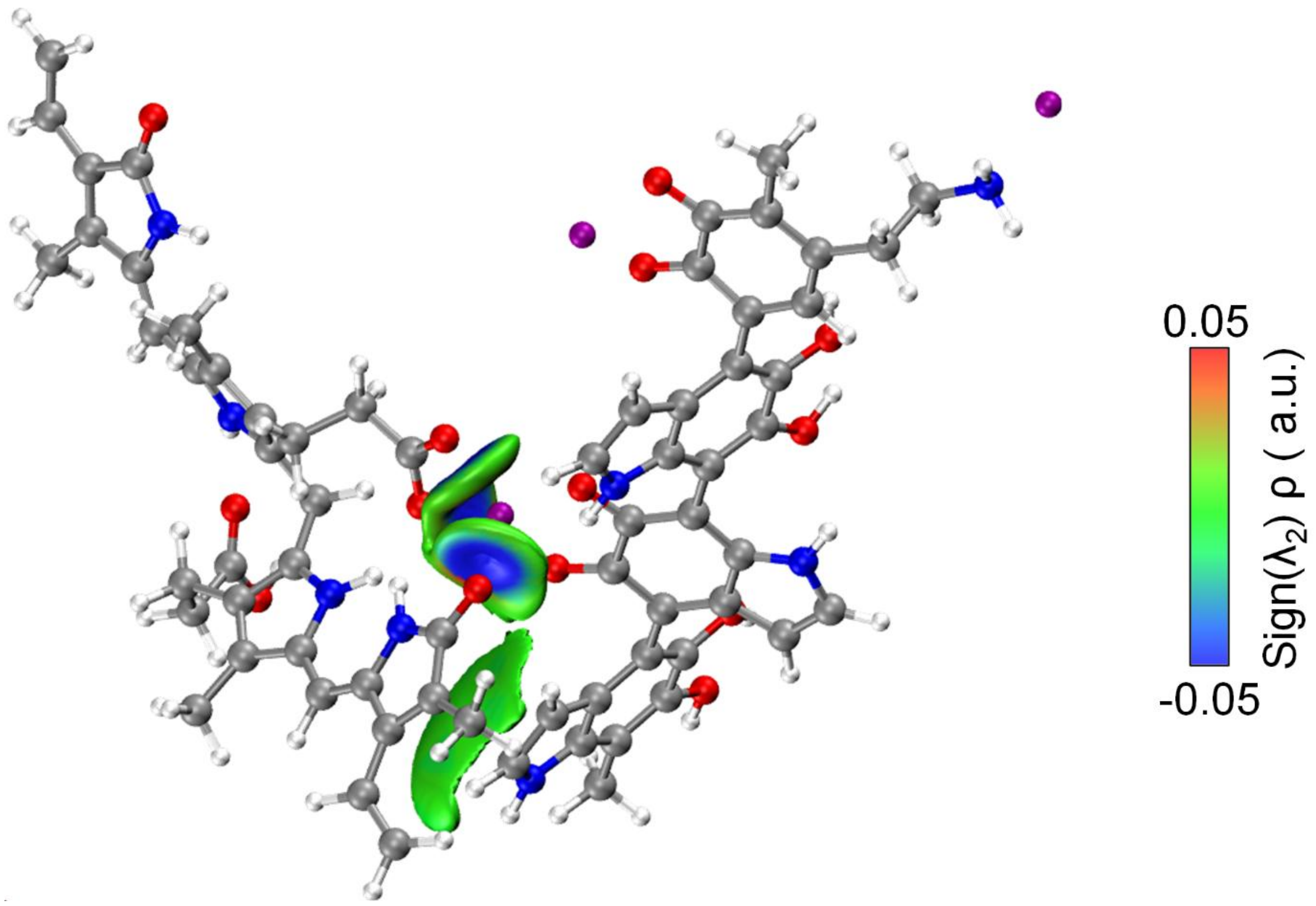

Figure S14. IGMH analysis of the bilirubin–PDA/Zn^{II} system. The blue regions indicate strong interactions, such as coordination interactions and hydrogen bonding. The green regions represent weak interactions, including π–π stacking, van der Waals forces, and general hydrophobic interactions. The red regions correspond to repulsive interactions, typically arising from steric hindrance or unfavorable close atom contacts.

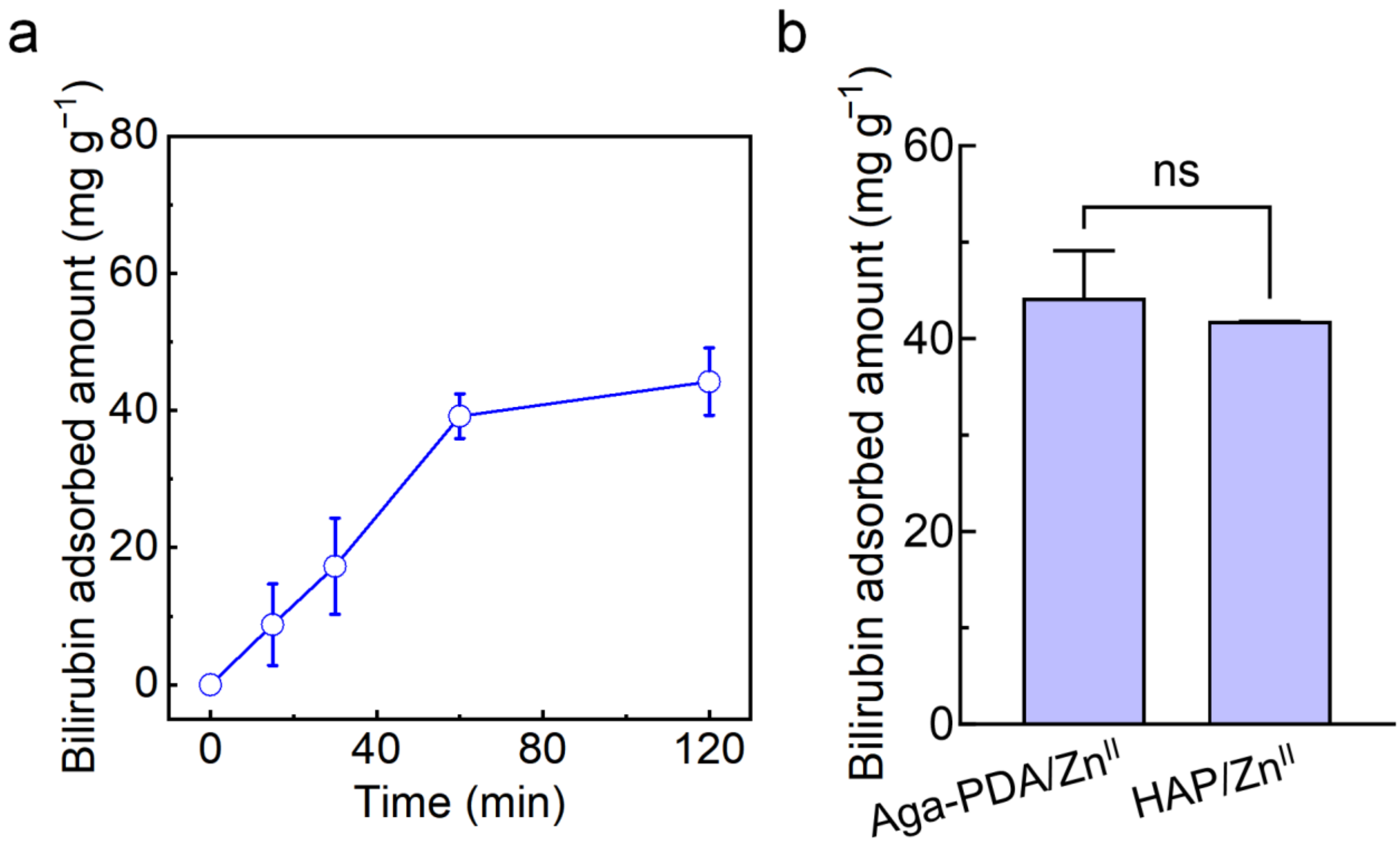

Figure S15. (a) Bilirubin adsorption capacity of Aga-PDA/Zn^{II} in simulated plasma as a function of contact time (initial concentration of bilirubin = 200 mg L^{-1}, dosage of Aga-PDA/Zn^{II} = 0.8 g L^{-1}). (b) Bilirubin adsorption capacity of Aga-PDA/Zn^{II} and HAP/Zn^{II} at 120 min. Data are presented as mean ± standard deviation ($n \geq 3$). Unpaired *t*-tests: ns, not significantly different.

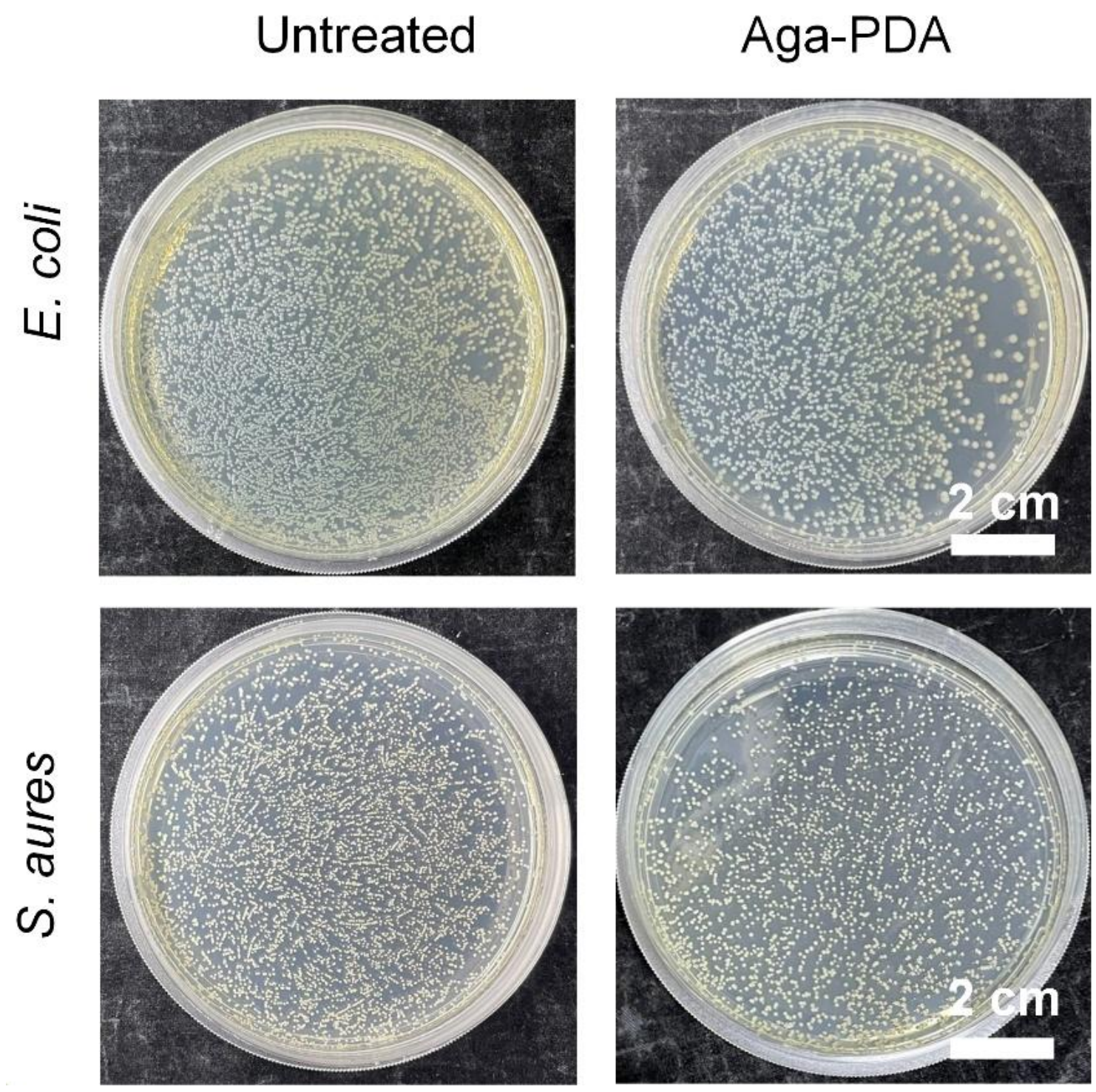

Figure S16. Optical images showing the growth of *E. coli* and *S. aureus* on agar plates after 24 h of co-culture with Aga-PDA or untreated groups.

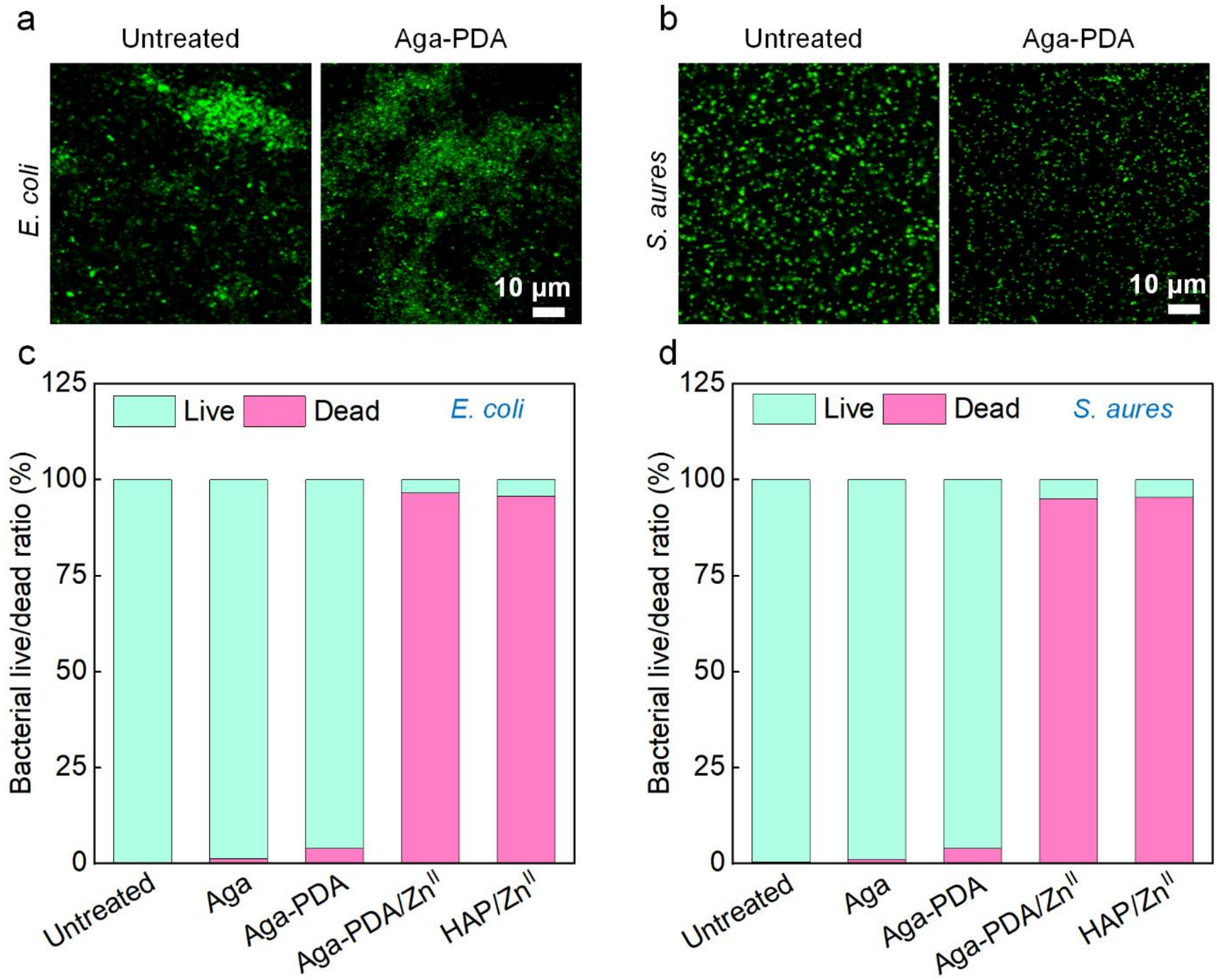

Figure S17. Live/dead fluorescence images of (a) *E. coli* and (b) *S. aureus* after contact-killing by Aga-PDA compared with untreated groups. (c,d) Quantitative bacterial live/dead ratios of (c) *E. coli* and (d) *S. aureus* from fluorescence images.

The bacterial mortality rates in the Aga treatment group were 1.3% for *E. coli* and 1.0% for *S. aureus*. After treatment with Aga-PDA, the mortality rates only increased slightly, reaching 3.9% for both *E. coli* and *S. aureus*. In contrast, treatment with Aga-PDA/Zn^{II} significantly increased bacterial mortality, reaching 96.5% for *E. coli* and 95.1% for *S. aureus*. After treatment with HAP/Zn^{II}, the mortality rates of *E. coli* and *S. aureus* were 95.8% and 95.4%, respectively, showing no significant difference compared with Aga-PDA/Zn^{II}. These results further confirm that HAP/Zn^{II} retains the PDA/Zn^{II} network and exhibits broad-spectrum antibacterial activity against both *E. coli* and *S. aureus*.

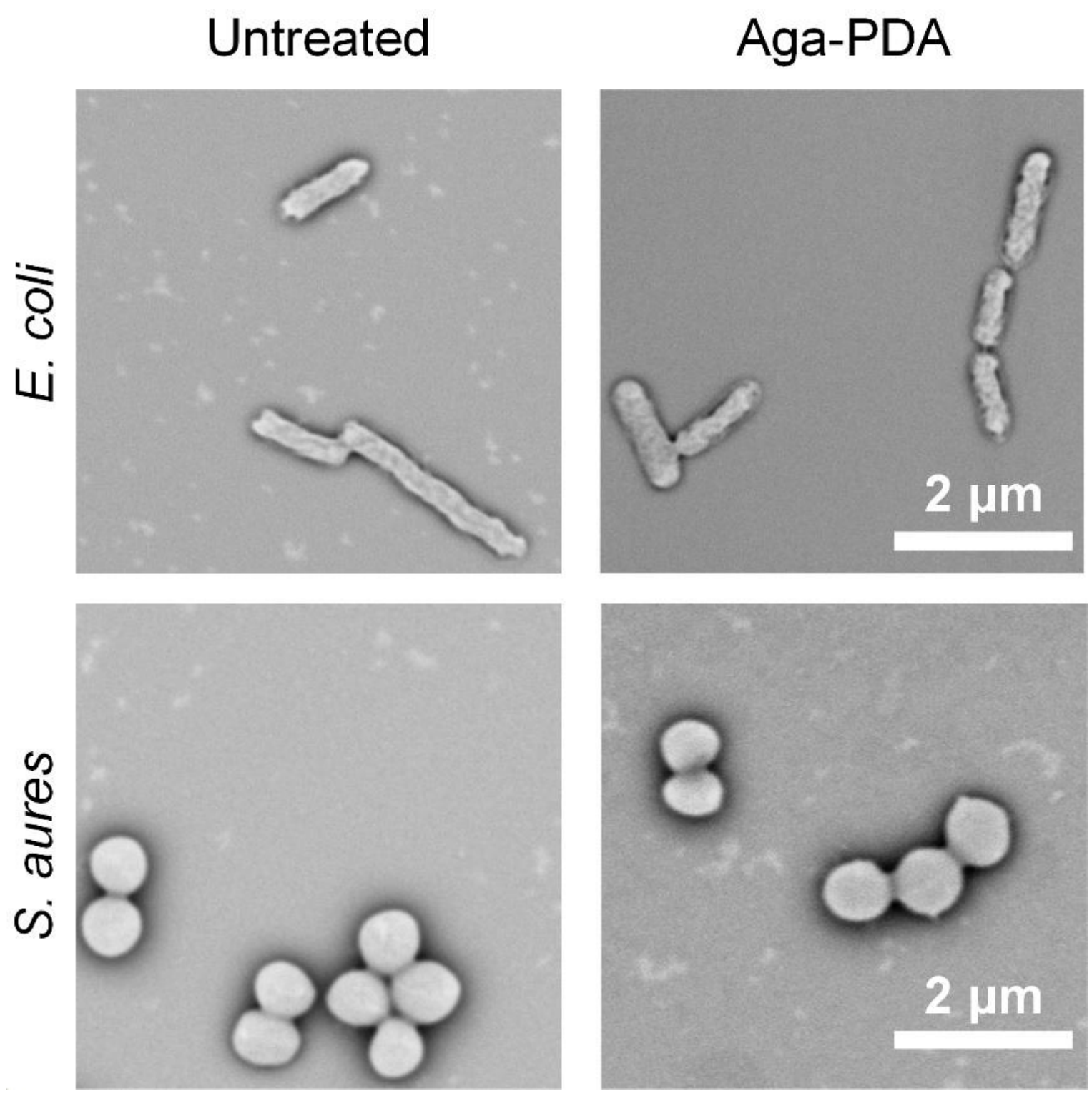

Figure S18. SEM images of *E. coli* and *S. aureus* before and after contact sterilization by Aga-PDA.

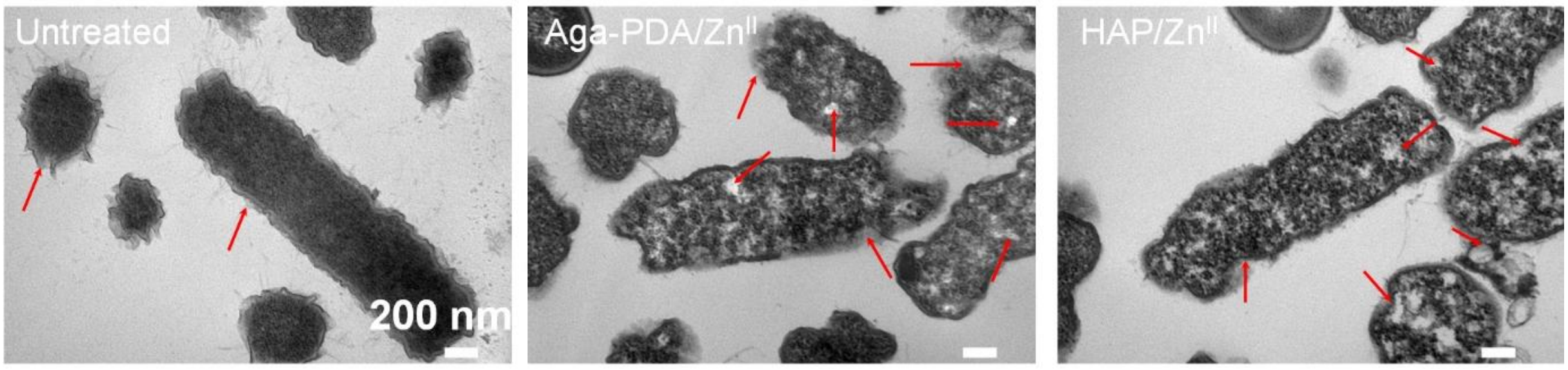

Figure S19. TEM images showing the morphological changes of *E. coli* and *S. aureus* in a mixed bacterial suspension before and after treatment with Aga-PDA/Zn^{II} or HAP/Zn^{II}.

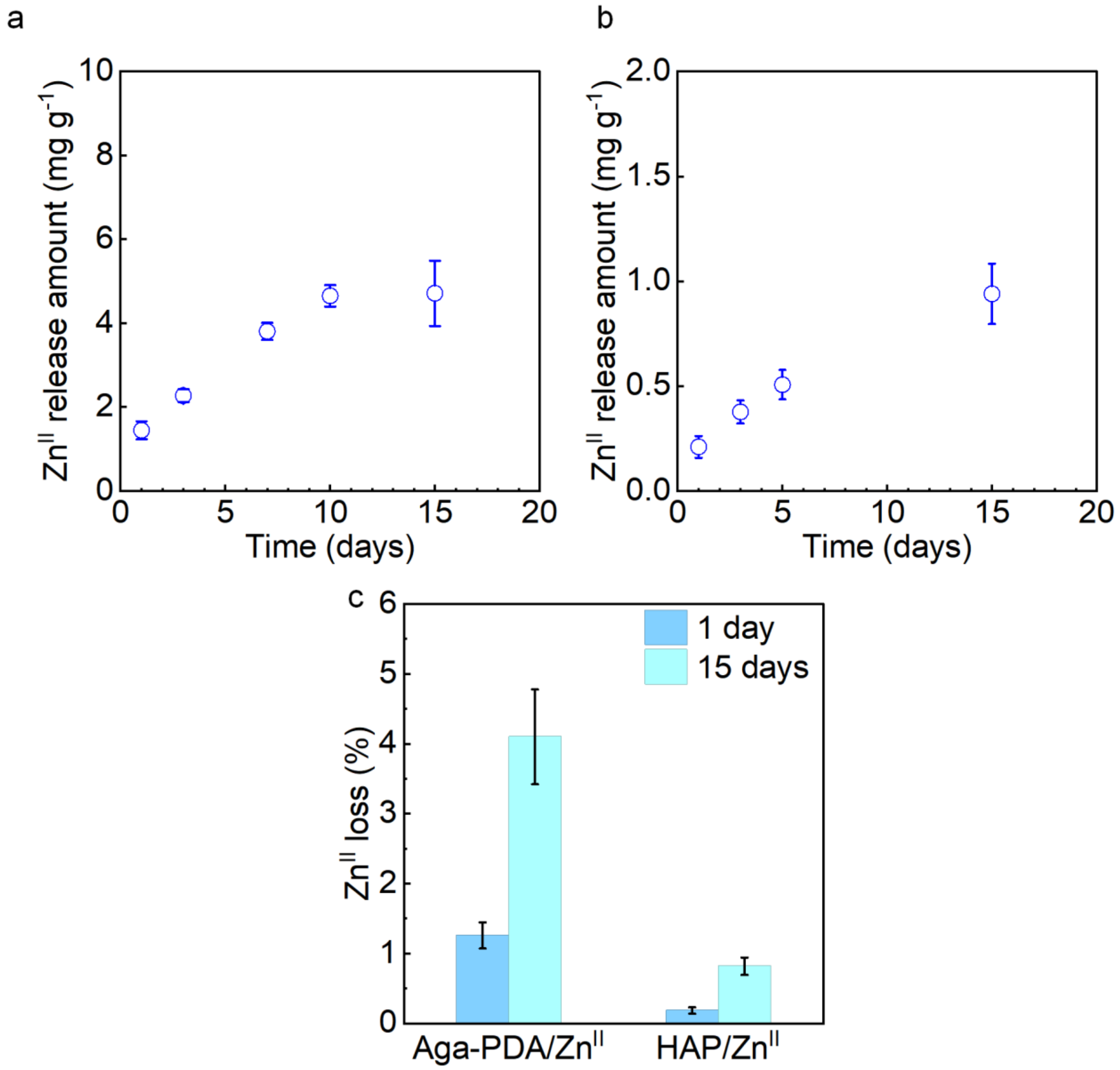

Figure S20. Cumulative Zn^{II} release profiles from (a) Aga-PDA/Zn^{II} and (b) HAP/Zn^{II} as a function of time. (c) Percentage of Zn^{II} loss of Aga-PDA/Zn^{II} or HAP/Zn^{II} after 1 day and 15 days, calculated by comparing the amount of released zinc ions with the total zinc content in the PDA/Zn^{II} framework of Aga-PDA/Zn^{II} or HAP/Zn^{II}. Data are presented as mean ± standard deviation ($n \geq 3$).

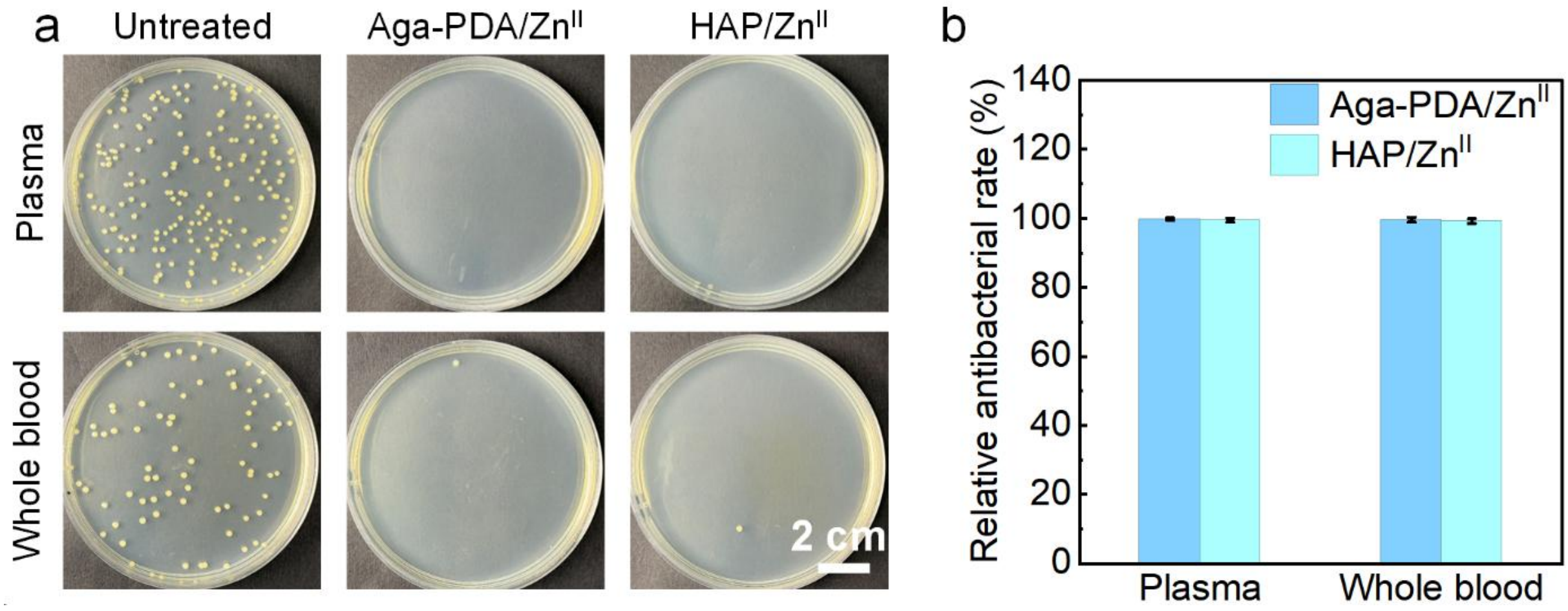

Figure S21. (a) Representative photographs of bacterial colonies on agar plates after co-incubation of *S. aureus* with Aga-PDA/Zn^{II} or HAP/Zn^{II} in 50% plasma or 50% whole blood for 5 h. (b) Relative antibacterial activity of Aga-PDA/Zn^{II} and HAP/Zn^{II} against *S. aureus* under the corresponding conditions. In (b), data are presented as mean ± standard deviation ($n \geq 3$).

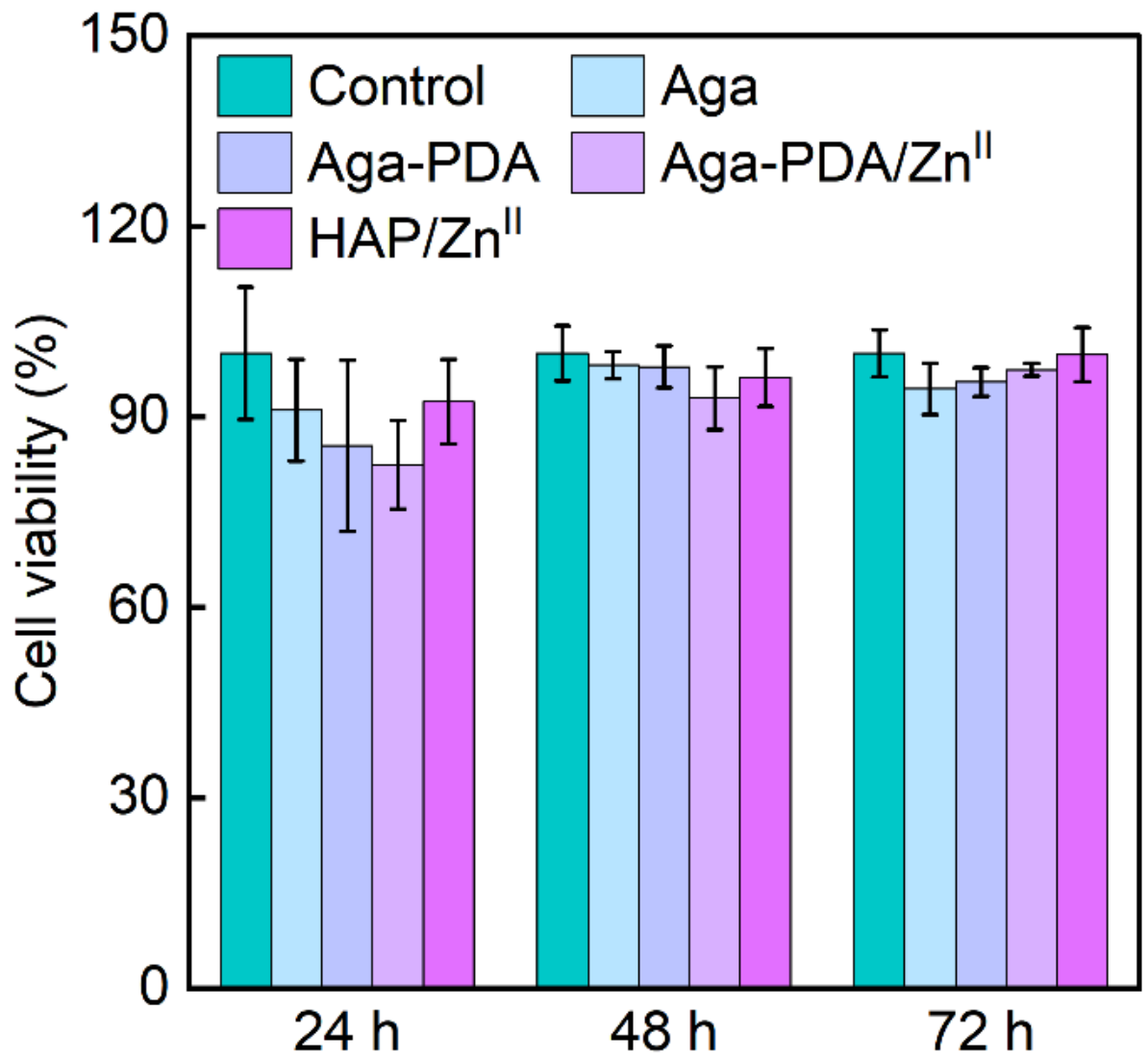

Figure S22. Cell viability of L929 cells after incubation with Aga, Aga-PDA, Aga-PDA/Zn^{II}, and HAP/Zn^{II} for 24, 48, and 72 h. Control, untreated cells; concentration of samples, 250 μg mL^{-1}. Data are presented as mean ± standard deviation ($n \geq 3$).

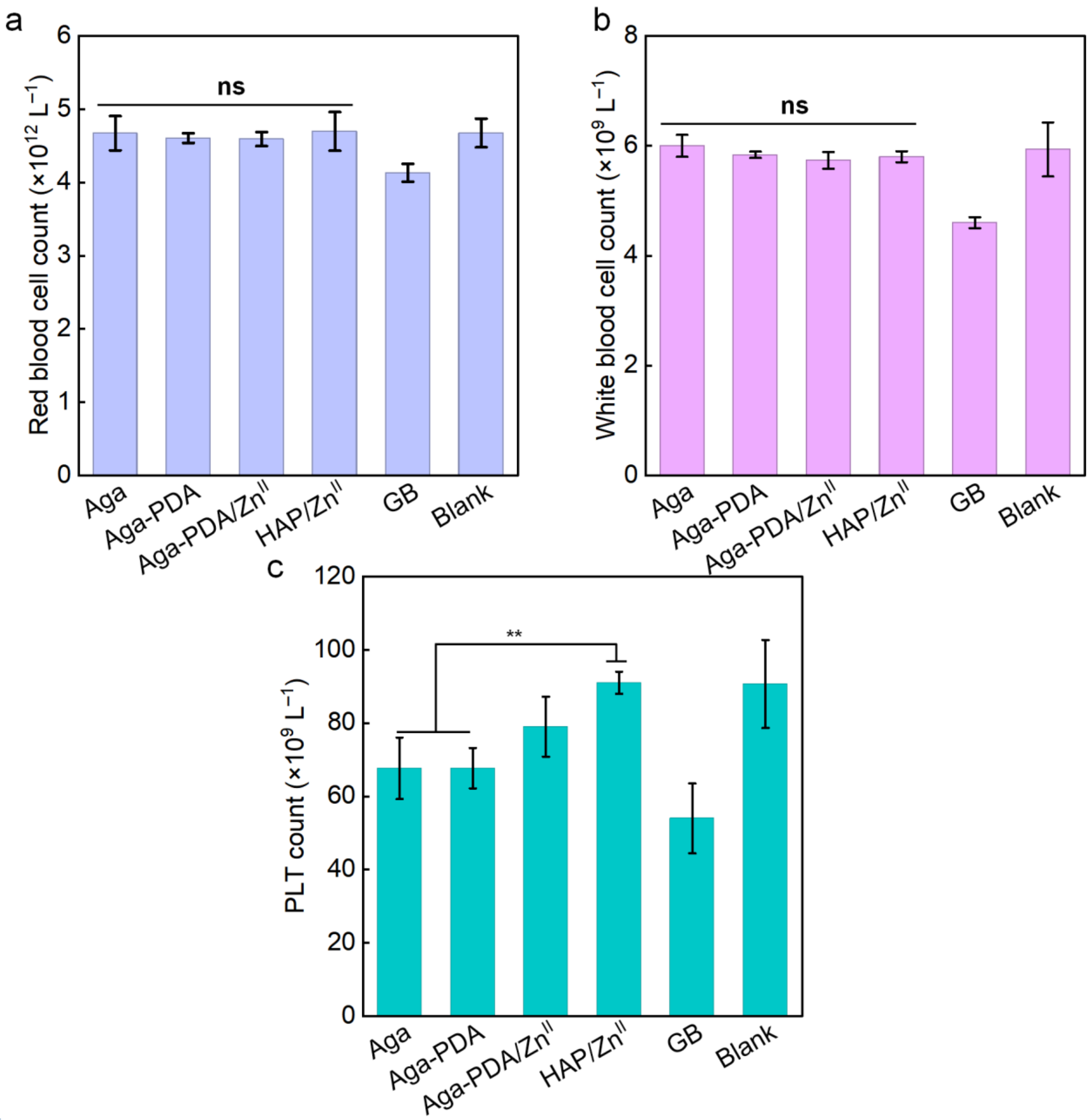

Figure S23. (a) Red blood cell, (b) white blood cell, and (c) platelet (PLT) counts after co-incubation of Aga, Aga-PDA, Aga-PDA/Zn^{II}, HAP/Zn^{II}, or glass beads (GB) with whole blood, as analyzed using a hematology analyzer. Blank, whole blood without samples; data are presented as mean ± standard deviation ($n \geq 3$); one-way analysis of variance: ns, not significantly different, $^{**}P < 0.0021$.

The red blood cell counts for the Aga, Aga-PDA, Aga-PDA/Zn^{II}, HAP/Zn^{II}, GB, and blank control groups were 4.67, 4.60, 4.59, 4.70, 4.13, and 4.67 × 10^{12} L^{-1}, respectively. The corresponding white blood cell counts were 6.00, 5.83, 5.73, 5.80, 4.60, and 5.93 × 10^9 L^{-1}. PLT count is commonly used to evaluate coagulation function. In the GB positive control group, the PLT count decreased significantly to 54.0 × 10^9 L^{-1} relative to the saline negative control group (90.7 × 10^9 L^{-1}). Similarly, the Aga, Aga-PDA, and Aga-PDA/Zn^{II} groups showed

reduced PLT counts (67.7, 67.6, and 79.0 × 10^9 L^{-1}, respectively) relative to the control. In contrast, the PLT count of the HAP/Zn^{II} group remained high at 91.0 × 10^9 L^{-1}, indicating that HSA modification effectively reduces PLT adhesion and activation, thereby further inhibiting coagulation.

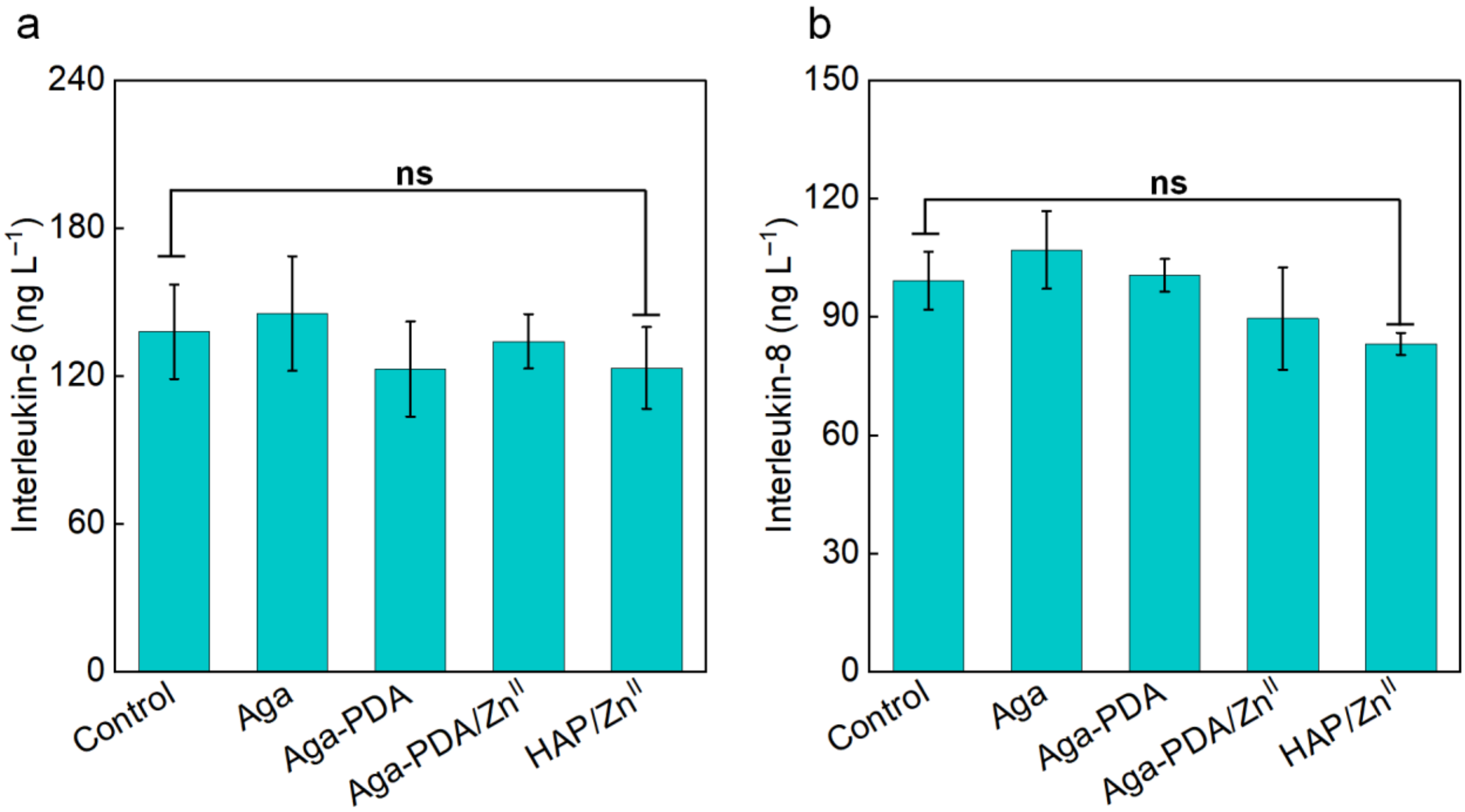

Figure S24. Levels of immune-related cytokines (a) interleukin-6 and (b) interleukin-8 in plasma after co-incubation with Aga, Aga-PDA, Aga-PDA/Zn^{II}, or HAP/Zn^{II}. Control, neat plasma; data are presented as mean ± standard deviation ($n \geq 3$); one-way analysis of variance: ns, not significantly different.

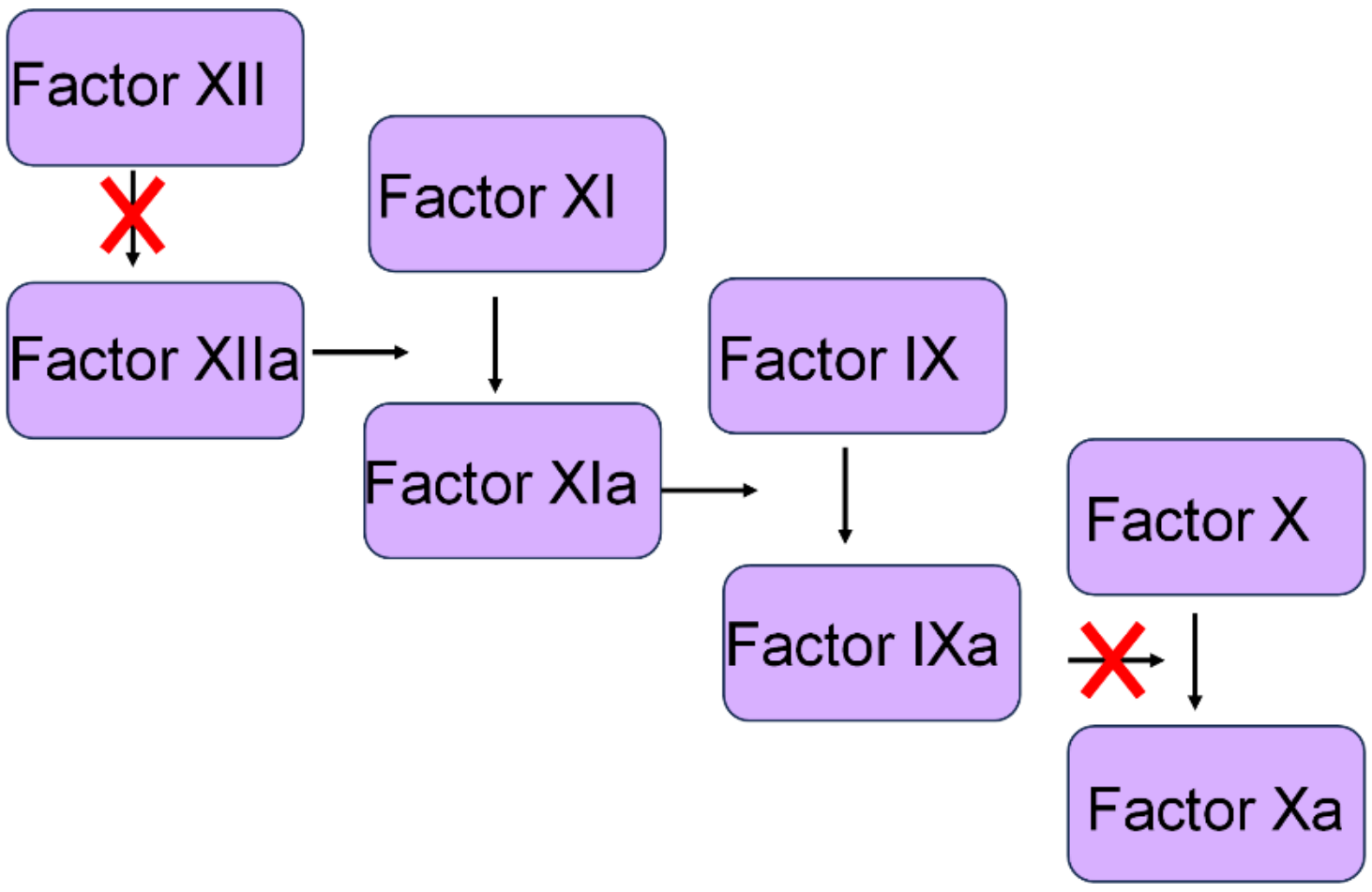

Figure S25. Schematic illustration of the anticoagulation mechanism of HAP/Zn^{II} superstructures.

Table S1. EXAFS fitting parameters at the Zn K-edge for various samples (S_0^2 = 0.80)

Sample	Shell	CN^a	R (Å)^b	σ^2 (Å^2)^c	ΔE_0 (eV)^d	R factor
Zn foil	Zn–Zn	6^e	2.64 ± 0.01	0.0085	4.3 ± 1.3	0.0058
	Zn–Zn	6^e	2.77 ± 0.03	0.0151		
Aga-PDA/Zn^{II}	Zn–N/O	6.2 ± 0.2	1.99 ± 0.01	0.0121	−1.0 ± 0.7	0.0051
	Zn–Zn	5.2 ± 0.6	3.14 ± 0.01	0.0186		
ZnO	Zn–O	4.3 ± 0.4	1.96 ± 0.01	0.0044	2.6 ± 1.3	0.0086
	Zn–Zn	10.3 ± 0.7	3.22 ± 0.01	0.0067		
	Zn–O1	12.9 ± 2.4	3.73 ± 0.03	0.0031		
ZnPc	Zn–N	4.4 ± 0.4	2.00 ± 0.01	0.0040	2.0 ± 2.0	0.0187
	Zn–Zn	4.9 ± 0.9	3.00 ± 0.02	0.0063		
$Zn(OH)_2$	Zn–O	6.1 ± 0.3	2.07 ± 0.01	0.0115	9.8 ± 1.1	0.0127
	Zn–Zn	7.1 ± 0.7	3.19 ± 0.01	0.0123		

$^a CN$: coordination number; $^b R$: bond distance; $^c \sigma^2$: Debye–Waller factor; $^d \Delta E_0$: inner potential correction; ^{e}Data from standard (reference) sample. R factor: goodness of fit. S_0^2 was set to 0.80, according to the experimental EXAFS fit of Zn foil reference by fixing CN as the known crystallographic value.

Table S2. Relative surface chemical composition of different samples

Samples	Chemical composition of surface (mol%)			
	C	N	O	Zn
Aga	64.74	0	35.26	0
Aga-PDA	66.72	3.89	29.39	0
Aga-PDA/Zn^{II}	58.66	4.02	30.46	6.86
HAP/Zn^{II}	61.91	4.84	32.03	1.22

Table S3. Peak areas of C 1s spectra of different superstructures

Superstructures	Relative area corresponding to different chemical bonds (%)				
	C–C/C–H	C–N	C–O	C=O	O–C=O
Aga-PDA/Zn^{II}	40.58	16.41	18.09	13.90	11.02
HAP/Zn^{II}	31.00	26.65	17.69	20.88	3.79

Table S4. Molecular docking results

Mode	Affinity (kcal mol^{-1})	rmsd l.b	rmsd u.b
1	−9.9	0.000	0.000
2	−9.1	34.846	37.887
3	−8.9	52.844	55.703
4	−8.9	39.519	42.211
5	−8.7	1.391	1.968
6	−8.6	37.964	41.528
7	−8.6	38.056	41.504
8	−8.5	37.401	40.838
9	−8.5	58.894	61.384

The table lists the binding affinities and the corresponding lower and upper bounds of the root mean square deviation (rmsd l.b. and rmsd u.b., respectively) for nine binding modes. A lower binding energy indicates a more stable interaction between the ligand and the receptor. Generally, a docking energy lower than −4.25 kcal mol^{-1} suggests some binding activity, a docking energy lower than −5.0 kcal mol^{-1} indicates good binding activity, and a docking energy lower than −7.0 kcal mol^{-1} represents strong binding activity.[9]

Table S5. Comparison of bilirubin adsorption, anticoagulant, and antimicrobial properties of different adsorbents

Adsorbent	Adsorbate	Initial concentration of bilirubin (mg L^{-1})	Adsorbent/ adsorbate (mg mL^{-1})	Adsorption capacity (mg g^{-1})	Equilibrium time (min)	Anticoagulation (APTT prolonged)	Antibacterial	Ref.
CGNPs	simulated plasma	267	20	6.6	120	/	/	[10]
Mesoporous carbon spheres	bilirubin	250	5	148.4	120	/	/	[11]
OPCM-10%	40 g L^{-1} BSA	200	1	9.8	120	/	/	[12]
NK110-Col-Hep	bilirubin<br>50 g L^{-1} BSA	200	10	37.0<br>7.0	180	~5 times	/<br>/	[13]
PS-DVB/V-Hap3	bilirubin	150	/	7.4	120	/	/	[14]
CM@PTL	bilirubin<br>50 g L^{-1} BSA	200	4	36.2<br>22.1	240	~2.3	/<br>/	[15]
IOBs	bilirubin<br>plasma	200	10	332.0<br>52.0	120	<2 times	/<br>/	[16]
PEI/MFC cryogel	bilirubin<br>40 g L^{-1} BSA	200	2	103.6<br>67.8	350	<2 times	/<br>/	[17]
Col-PEI microspheres	bilirubin<br>50 g L^{-1} BSA	200	1	125.0<br>31.5	180	2–3 times	/<br>/	[18]
Ch/GO	bilirubin	200	0.5	325.5	120	<1.5 times	/	[19]
ColAL	bilirubin<br>40 g L^{-1} BSA	200	1.67	152.2<br>102.3	240	~2 times	/<br>/	[20]
PCB-H103<br>H103 resin beads	40 g L^{-1} BSA<br>40 g L^{-1} BSA	150	3	20.8<br>3.0	360	/<br>/	/<br>/	[21]
P-2 particle	bilirubin	150	3.2	31.2	150	~1.5 times	/	[22]
iPAF-6	50 g L^{-1} BSA	200	1	192.3	40	~1 times	/	[23]
HAP/Zn^{II}	bilirubin<br>50 g L^{-1} BSA<br>simulated plasma	200	0.8	212.9<br>53.9<br>41.9	60<br>120	2.6 times	> 99.9%	This work

CGNPs: cubic gel nanoparticles; OPCM-10%: polyvinyl alcohol microspheres with 10% ordered mesoporous silica; BSA: bovine serum albumin; NK110-Col-Hep: NK110 microspheres coated with multiple alternating layers of collagen and heparin; PS-DVB/V-Hap3: inorganic–organic co-cross-linked nanocomposite network constructed from nonionic

polystyrene-divinylbenzene resin and hydroxyapatite nanoparticles functionalized with 3 wt% vinyltriethoxysilane; CM@PTL: cellulose microspheres coated with a shell of phase-transformed lysozyme; IOBs: inverse opal beads; PEI/MFC cryogel: cryogel made of polyethyleneimine reinforced with microfibrillated cellulose; Col-PEI microspheres: collagen microspheres modified with polyethyleneimine; Ch/GO: chitin combined with graphene oxide to form composite beads; ColAL: collagen-based artificial liver material made from collagen fibers modified with polyethyleneimine; PCB-H103: H103 polystyrene resin microparticles coated with a zwitterionic poly(carboxybetaine) hydrogel layer; P-2 particle: poly(glycidyl methacrylate-*co*-N-vinylpyrrolidone) particles prepared with an N-vinylpyrrolidone content of 20%; iPAF-6: cationic porous aromatic framework made with chloride ions replacing bromide through ion exchange; /: not measured. Unlike most conventional adsorbents that display efficient bilirubin removal, while failing to simultaneously address anticoagulant and antibacterial performance, the HAP/Zn^{II} superstructures effectively integrated rapid bilirubin clearance with anticoagulant and antibacterial properties, highlighting their potential for blood purification.

References

[1] R. Apweiler, A. Bairoch, C. H. Wu, et al., "UniProt: The Universal Protein Knowledgebase," *Nucleic Acids Research* **45**, (2017): D158–D169, https://10.1093/nar/gkw1099.

[2] J. Eberhardt, D. Santos-Martins, A. F. Tillack, S. Forli, "AutoDock Vina 1.2.0: New Docking Methods, Expanded Force Field, and Python Bindings," *Journal of Chemical Information and Modeling* **61**, (2021): 3891–3898, https://10.1021/acs.jcim.1c00203.

[3] O. Trott, A. J. Olson, "AutoDock Vina: Improving the Speed and Accuracy of Docking with a New Scoring Function, Efficient Optimization, and Multithreading," *Journal of Computational Chemistry* **31**, (2009): 455–461, https://10.1002/jcc.21334.

[4] D. Seeliger, B. L. de Groot, "Ligand Docking and Binding Site Analysis with PyMOL and Autodock/Vina," *Journal of Computer-Aided Molecular Design* **24**, (2010): 417–422, https://10.1007/s10822-010-9352-6.

[5] R. A. Laskowski, M. B. Swindells, "LigPlot+: Multiple Ligand–Protein Interaction Diagrams for Drug Discovery," *Journal of Chemical Information and Modeling* **51**, (2011): 2778–2786, https://10.1021/ci200227u.

[6] T. Lu, Q. Chen, "Independent Gradient Model Based on Hirshfeld Partition: A New Method for Visual Study of Interactions in Chemical Systems," *Journal of Computational Chemistry* **43**, (2022): 539–555, https://10.1002/jcc.26812.

[7] T. Lu, F. Chen, "Multiwfn: A Multifunctional Wavefunction Analyzer," *Journal of Computational Chemistry* **33**, (2012): 580–592, https://10.1002/jcc.22885.

[8] A. D. William Humphrey, and Klaus Schulten, "VMD: Visual Molecular Dynamics," *Journal of Molecular Graphics* **14**, (1996): 33–38.

[9] K. Y. Hsin, S. Ghosh, H. Kitano, "Combining Machine Learning Systems and Multiple Docking Simulation Packages to Improve Docking Prediction Reliability for Network Pharmacology," *Public Library of Science ONE* **8**, (2013): e83922, https://10.1371/journal.pone.0083922.

[10] J. Yang, Y. Yuan, J. Hou, et al., "Cyclodextrin Metal–Organic Framework Derived Nano-Cubic Gel Particles for the Efficient Removal of Bilirubin," *Separation and Purification Technology* **360**, (2025), 131071, https://10.1016/j.seppur.2024.131071.

[11] L. Guo, J. Zhang, Q. He, et al., "Preparation of Millimetre-Sized Mesoporous Carbon Spheres as an Effective Bilirubin Adsorbent and Their Blood Compatibility," *Chemical Communications* **46**, (2010): 7127–7129, https://10.1039/c0cc02060e.

[12] M. Zhang, Y. Wang, W. Zhou, et al., "Ordered Mesoporous SiO_2 as Hemoperfusion Adsorbents for Enhanced Selectivity of Bilirubin Removal from Blood," *Separation and Purification Technology* **359**, (2025), 130640, https://10.1016/j.seppur.2024.130640.

[13] W. Zhou, H. Yu, B. Zhu, et al., "Collagen and Heparin Co-Modification of NK110 Microspheres for Enhanced Bilirubin Adsorption and Improved Blood Compatibility in Hemoperfusion," *Separation and Purification Technology* **372**, (2025), 133541, https://10.1016/j.seppur.2025.133541.

[14] Y. Chai, Z. Liu, Y. Du, et al., "Hydroxyapatite Reinforced Inorganic-Organic Hybrid Nanocomposite as High-Performance Adsorbents for Bilirubin Removal In Vitro and in Pig Models," *Bioactive Materials* **6**, (2021): 4772–4785, https://10.1016/j.bioactmat.2021.05.017.

[15] C. Guo, J. Gao, C. Wang, et al., "Surface Engineering of Cellulose Microspheres Via Amyloid-Like Protein Aggregation for Bilirubin Removal," *Carbohydrate Polymers* **368**, (2025): 124145, https://10.1016/j.carbpol.2025.124145.

[16] Y. Wang, X. Zhou, X. Yang, et al., "Strategic Nanoarchitectonics of Porous Beads for Protein-Bound Toxin Removal with Self-Photodecomposition and Visual Monitoring for Hyperbilirubinemia," *Advanced Functional Materials* **35**, (2025), 2502795, https://10.1002/adfm.202502795.

[17] Y. Cui, X. Zhang, G. Chen, et al., "Microfibrillated Cellulose Reinforced Polyethyleneimine Cryogels Fabricated Via Cryo-Induced Chemical Crosslinking for Enhanced Removal of Albumin-Bound Bilirubin," *International Journal of Biological Macromolecules* **306**, (2025): 141595, https://10.1016/j.ijbiomac.2025.141595.

[18] W. Zhou, W. Hu, Q. Zhan, et al., "Novel Hemoperfusion Adsorbents Based on Collagen for Efficient Bilirubin Removal – A Thought from Yellow Skin of Patients with Hyperbilirubinemia," *International Journal of Biological Macromolecules* **253**, (2023): 127321, https://10.1016/j.ijbiomac.2023.127321.

[19] X. Song, X. Huang, Z. Li, et al., "Construction of Blood Compatible Chitin/Graphene Oxide Composite Aerogel Beads for the Adsorption of Bilirubin," *Carbohydrate Polymers* **207**, (2019): 704–712, https://10.1016/j.carbpol.2018.12.005.

[20] M. Li, X. Wang, G. Gong, et al., "Engineered Liver-Inspired Collagen Matrix as a High-Performance Hemoperfusion Adsorbent for Bilirubin Removal," *Chemical Engineering Journal* **426**, (2021), 130791, https://10.1016/j.cej.2021.130791.

[21] Q. Li, J. Yang, N. Cai, et al., "Hemocompatible Hemoadsorbent for Effective Removal of Protein-Bound Toxin in Serum," *Journal of Colloid and Interface Science* **555**, (2019): 145–156, https://10.1016/j.jcis.2019.07.045.

[22] X. Jiang, D. Zhou, X. Huang, et al., "Hexanediamine Functionalized Poly (Glycidyl methacrylate-*co*-N-vinylpyrrolidone) Particles for Bilirubin Removal," *Journal of Colloid and Interface Science* **504**, (2017): 214–222, https://10.1016/j.jcis.2017.05.039.

[23] R. Zhao, T. Ma, F. Cui, et al., "Porous Aromatic Framework with Tailored Binding Sites and Pore Sizes as a High-Performance Hemoperfusion Adsorbent for Bilirubin Removal," *Advanced Science* **7**, (2020): 2001899, https://10.1002/advs.202001899.
